# Protective Factors for Antisocial Behavior in Youth: What is the Meta-Analytic Evidence?

**DOI:** 10.1007/s10964-023-01878-4

**Published:** 2023-10-04

**Authors:** Jeanne Gubbels, Mark Assink, Claudia E. van der Put

**Affiliations:** Research Institute of Child Development and Education, University of Amsterdam, Nieuwe Achtergracht 127, 1018 WS Amsterdam, The Netherlands

**Keywords:** Antisocial behavior, Youth, Protective factors, Meta-analysis, Review

## Abstract

Although both risk and protective factors are important components of etiological theories for antisocial behavior, far less is known about protective factors and their impact. This review summarized primary studies on the impact of different protective factors for antisocial behavior in youth. In total, 305 studies reporting on 1850 potentially protective factors were included. Each extracted factor was first classified into one of 77 mutually exclusive groups of similar factors (referred to as domains), after which a three-level meta-analysis was conducted to determine the protective effect of each domain. A significant and negative effect was found for 50 domains, which were therefore designated as being truly protective. The largest impact (*r* < −0.20) was found for higher levels of conservativeness, self-transcendence, life satisfaction, involvement in romantic relationships, the capacity to reflect or mentalize, peer relationships quality, prosocial peers, prosocial values, agreeableness, school self-esteem, parental control, general resilience, and social skills. Analyses revealed that the impact of some of the 77 domains was moderated by the youth’s age (five domains) and gender (four domains) as well as the severity of antisocial behavior they exhibit (two domains), indicating that the impact of these domains differs across subgroups of antisocial youth. Given the substantial number of factors that were identified as being protective for antisocial behavior in youth, this study discusses implications for future directions, assessment strategies, and (preventive) interventions.

## Introduction

An abundance of research has been conducted to study risk and protective factors for antisocial behavior (see, for instance, Farrington et al., 2016; Hawkins et al., 1992; Loeber et al., 1998; 2008; Stouthamer-Loeber et al., 2002). Although much has been learned about the nature and impact of these factors, it is still far from clear why some individuals engage in antisocial behavior and others do not. Multiple reviews have appeared on risk factors for antisocial behavior (see, for example, Assink et al., 2015; Van Hazebroek et al., 2019; Loeber, 1990; Murray et al., 2018; Rosenfeld (2004); Vaughan et al., 2022), but far less is known about the factors that may protect against antisocial behavior and the strength of impact of these factors. This quantitative review aimed to fill this gap by synthesizing empirical studies on (potentially) protective factors for antisocial behavior in youth. Such a comprehensive synthesis of protective factors and their impact may serve to improve policies and interventions to elicit optimal behavioral outcomes.

From a clinical and preventive standpoint, it is particularly relevant to study etiological factors for antisocial behavior in the developmental stages of adolescence and youth. First, juvenile antisocial and criminal behavior is a serious societal problem with detrimental physical and mental health consequences for both victims and offenders (e.g., Burt et al., 2018; Cook et al., 2015; Piquero et al., 2007). Second, studies show that factors that predict chronic antisocial behavior and later delinquency, can already be recognized early in the adolescent’s life (Assink et al., 2015; Loeber et al., 2008). Finally, as children and adolescents grow older, problem behavior tends to become more stable, which makes it more difficult to change (Bernazzani et al., 2001; Brennan & Shaw, 2013). The life stages of adolescence and youth offer more opportunity for treating and preventing antisocial behavior than the stage of adulthood, and this review therefore studied the impact of protective factors in youth.

Antisocial behavior refers to a broad spectrum of actions that society considers as violating social norms, the law, and/or the rights of others (Calkins & Keane, 2009). This behavior can range from minor acts such as lying and bullying to more serious transgressions such as severe violence and committing assaults. It has been proposed that minor acts of antisocial behavior can evolve into more serious forms, for instance by Loeber et al. (1993) who described in his escalation model the existence of three developmental “pathways” that each describe how juveniles with certain minor problem behavior may escalate to the most serious behaviors. In her influential developmental taxonomy of antisocial behavior, Moffitt (1993) proposed two distinct types of antisocial juveniles based on the timing and duration of antisocial involvement: a large group showing antisocial behavior only during adolescence and a small group engaging in antisocial behavior over the life-course. *Adolescence-limited antisocial behavior* reflects a temporary involvement in antisocial behavior and is regarded as more or less normative in adolescence as a life phase. *Life-course persistent antisocial behavior*, on the other hand, starts at an early age with minor problem behaviors and continues through adolescence and into adulthood, escalating in more serious problem behavior.

However, since Moffitt posed her theory, evidence appeared that juveniles can desist from severe antisocial behavior during adolescence and that severe antisocial behavior emerging in adolescence is seldomly limited to this developmental stage (Fairchild et al., 2013). Also, a comparison of risk factors between life-course persistent and adolescence-limited offenders revealed differences in the amount of risk factors and not so much in the nature of risk factors (Assink et al., 2015). Many antisocial individuals struggle with drug and/or alcohol addictions, experience psychiatric problems, neuropsychological impairments, and have numerous social problems, such as unemployment, school problems, and homelessness (Assink et al., 2015; Dembo et al., 2008; Loeber & Farrington, 2000; Moffitt & Caspi, 2001). To reduce these problems, and to prevent that juveniles develop a persistent antisocial career, it is important to develop and advance existing etiological theories of antisocial behavior. To do so, researchers have paid much attention to risk factors and their impact, which may have been driven by cumulative and multiple-risk models (i.e., Loeber et al., 2008) as well as the emphasis in clinical practice on the importance of risk factors (as described by the principles of the RNR model; Andrews & Bonta, 1990; 2010). However, protective factors have not been studied so extensively even though identifying and understanding protective factors are important for advancing etiological knowledge.

Risk factors increase the probability of an undesirable outcome, such as offending, whereas protective factors reduce the probability of an undesirable outcome. The definition of protective factors is broadly discussed in the literature (Farrington et al., 2016; Klepfisz et al., 2017). Some researchers have defined a protective factor as the “mirror image” of a risk factor (e.g., White et al., 1989; Klepfisz et al., 2020), raising the question whether factors are unipolar or bipolar, that is, whether risk factors and protective factors are fundamentally different or similar in nature with a “risk side” and a “protective side” (Eisenberg et al., 2022; Stouthamer-Loeber et al., 1993). For example, the factor “school achievement” can be dichotomized into the risk factor “low school achievement” (poor grades), and the protective factor “high school achievement” (high grades; Loeber et al., 2008; Van der Put et al., 2011a). The impact of both types of protective factors were examined here. That is, this review synthesized the effects of both unipolar protective factors (such as support from a stable intimate relationship) as bipolar protective factors (such as school achievement).

Other researchers distinguish *protective factors*, that moderate the impact of risks on problem behavior (e.g., Herrenkohl et al., 2003; Rutter, 1987; 2003), from *promotive factors*, that have a direct negative effect on problem behavior (Farrington et al., 2008; Loeber et al., 2008; Sameroff et al., 1998; Stouthamer-Loeber et al., 2002). For the current review, protective factors were defined as factors that are associated with a lower probability of antisocial behavior. This definition is more in line with that of *promotive factors* (Sameroff et al., 1998) or *direct protective factors* (Lösel & Farrington, 2012) than the definition of *protective* or *buffering protective factors*, as the aim was to summarize evidence on the direct association between a (potentially protective) factor and antisocial behavior in youth, rather than the buffering effect of a factor in the presence of one or more risk factors. Consensus on terminology for factors that reduce the probability of a negative outcome has not yet been reached in the literature. Therefore, the term *protective factors* was used to refer to those factors.

Antisocial behavior can be seen as the result of complex interactions between risk and protective factors rather than a simple cause and effect relationship (e.g., Deković & Prinzie, 2008; Prinzie et al., 2008; Van der Put et al., 2011a). This view on how antisocial behavior evolves is in line with the ecological perspective on child development of Bronfenbrenner (1979, 1986). Bronfenbrenner stated that the individual child interacts with different social ecological systems surrounding the child, such as the family, peers, and the school environment (microsystem), the extended family (exosystem), and the culture, laws, and social-political conditions (macrosystem). In each of these systems, risk and protective factors can be present that both influence the risk of negative developmental outcomes, such as antisocial behavior. Bronfenbrenner assumed that factors in more proximal social systems exert more influence on the individual’s development and behavior than factors in more distal social systems.

Since Bronfenbrenner (1979, 1986) posed his theory, it has been widely acknowledged that both risk and protective factors play an important role in explaining antisocial behavior (e.g., Deković & Prinzie, 2008; Prinzie et al., 2008; Van der Put et al., 2011a) and deserve attention in clinical practice. However, far less is known about protective factors than risk factors. Even though the impact of risk factors seems to be stronger than the impact of protective factors in developmental pathways to antisocial behavior (Deković, 1999), multiple domains of competent functioning can often be identified in individuals next to the presence of different risk factors. Further, positive youth development programs (Case (2017); Benson et al. (2006); Damon, 2004) and strength-based interventions (Fortune, 2018; Kewley, 2017; Olver et al., 2018) that focus on strengthening protective factors to promote the capability to cope with at-risk situations are more and more appreciated. These interventions are not only more attractive to communities than risk-oriented interventions (Pollard et al., 1999), but policy makers acknowledge that promoting healthy behavior and preventing at-risk behavior is more cost-effective than treating antisocial behavior (Kamerman, 2000). Moreover, the effect of interventions for preventing antisocial and delinquent behavior is greatest when they are aimed at both risk and protective factors for delinquency (Andrews et al., 2006; Bonta, 2002; Farmer et al., 2002; Lipsey & Derzon, 1998).

Previous review studies have already provided an overview of protective factors for antisocial behavior (e.g., Loeber, 1990; 2008; Lösel & Bender, 2017; Lösel & Farrington, 2012; Portnoy et al., 2013). Lösel and Farrington (2012), for example, provided a brief narrative review of research findings on protective factors and found several individual, family, school, peer, and neighborhood factors that could decrease the probability of youth violence. They found that the probability of violence decreases as the number of protective factors increases (a dose–response relationship) and that family-related factors were most protective. Portnoy et al. (2013) reviewed biological protective factors for antisocial and criminal behavior, and found that higher levels of IQ and a high resting heart rate may have protective effects. However, previously conducted review studies were merely qualitative in nature, making it difficult to draw inferences on the impact of these factors and to compare their impact. So far known, the empirical literature on protective factors for antisocial behavior has never been meta-analytically synthesized, which was the aim of the current study. In a meta-analysis, studies on (the impact of) protective factors can be summarized to increase insight into whether or not factors should indeed be designated as protective factors, and the overall impact of these factors. Accordingly, more insight can be gained into the factors that are truly protective and may play a role in preventing or reducing antisocial behavior.

Further, a meta-analysis allows for the examination of heterogeneity in effect sizes by testing variables as a moderator of an overall effect. This study examined how the impact of potentially protective domains are influenced by age, gender, and the type of antisocial behavior. First, it was assumed that effects of protective factors vary for different age groups, as previous studies showed substantial age differences in the prevalence of antisocial behavior (e.g., Moffit, 1993) and underlying factors explaining antisocial behavior (Loeber, 1990; Van der Put et al., 2011a, 2011b). Specifically, Van der Put et al., 2011a found that the impact of risk and protective factors on criminal recidivism was significantly larger in younger than in older adolescents. They also found that from age 14 protective factors in the school, relationship, and attitude domains best predict recidivism. Second, gender may influence the impact of protective factors, as gender differences in the impact of factors for delinquency and antisocial behavior have been identified (Funk, 1999; Schwalbe et al., 2006; Scott & Brown, 2018; Van der Put et al., 2011b). For example, Scott and Brown found that affiliation with prosocial peers and the absence of substance abuse was significantly related to less recidivism for boys and girls, but a stronger effect was found for boys. They also found that protective factors related to education and employment predicted the absence of recidivism in males, whereas youths’ prosocial values predict the absence of recidivism in females. Third, differences may exist in the impact of protective factors across types of antisocial behaviors. A recent study comparing risk factors across delinquency types found that a high level of pro-violence attitudes was significantly associated with violent offending, whereas a low level of self-control was a significant predictor of nonviolent offending and general delinquency (Chan, 2021). Further, Liu and Miller (2020) found that parental attachment inhibited non-aggressive delinquency, but not aggressive delinquency. Therefore, the impact of protective factors was examined across type of antisocial behavior, and specifically across “less severe” antisocial behavior such as behavioral problems, externalizing behavior, and rule-breaking behavior, and “more severe” antisocial behavior such as delinquency, assault, and violence.

## Current Study

As the literature on protective factors for antisocial behavior in youth had not been summarized quantitively, this study synthesized all available empirical evidence on protective factors for all behavior in youth that violates social norms, the law, and/or the rights of others (i.e., antisocial behavior). Specifically, this study examined what factors can be designated as being truly protective against antisocial behavior in youth (Research Question 1), and what is the strength of the association between these factors and antisocial behavior in youth (Research Question 2). In answering these questions, each putative protective factor that was examined in a primary study was classified into one of many “domains” of protective factors, which is defined as a (broad) group of protective factors that are similar in nature (see also, Assink et al., 2015, 2019, and Gubbels et al., 2019 for this analytic strategy). For each of these potentially protective domains, an overall impact was estimated in a separate meta-analysis. This study also examined how the impact of protective domains is influenced by age, gender, and the type of antisocial behavior (Research Question 3). It was expect that the impact of protective domains differs across the age of youth (Hypothesis 1), across male and female youth (Hypothesis 2), and across severity of antisocial behavior (Hypothesis 3).

## Methods

### Inclusion and Exclusion Criteria

To select relevant studies, several inclusion and exclusion criteria were formulated. First, studies had to examine the impact of at least one (potential) protective factor for antisocial behavior. Antisocial behavior was broadly defined as actions that psychically or psychologically harm others, in which the perpetrator considers the well-being of others insufficiently, or in the most severe cases, violates the basic rights of others (Berger, 2003; Calkins & Keane, 2009; Stoff et al., 1997). Therefore, primary studies examining the following concepts were included: aggression, anger, behavior problems, externalizing behavior, rule breaking behavior, bullying, conduct disorder (CD), oppositional defiant disorder (ODD), delinquent behavior, various crimes, violence, and recidivism. Primary studies that measured antisocial behavior in specific settings (e.g., institutional or workplace aggression), in specific populations (e.g., visually and/or hearing-impaired children, elderly, homeless people), as well as specific forms of antisocial behavior (e.g., sexual violence, truancy, cybercrime, white collar crime, sex or labor trafficking, and terrorism) were excluded, as factors underlying these behaviors are very specific and difficult to generalize to antisocial behavior in a broader sense. Finally, studies reporting on alcohol abuse, drug abuse, and cigarette smoking were excluded as these problems were not regarded as antisocial behaviors in itself, unless studies reported on antisocial behavior resulting from alcohol and/or drug use. A *protective factor* was defined as any individual or contextual factor with the potential to reduce the probability of antisocial behavior. Studies were only included when they reported on a direct association between a (potential) protective factor and antisocial behavior in youth. Further, studies and the factors they examined were only included when primary study authors hypothesized that the factor(s) have a protective effect.

Second, studies with a prospective longitudinal research design (in which subjects were followed over time) as well as retrospective or cross-sectional studies (in which subjects were examined at a single point in time) were included. The search and selection of the studies was not restricted to longitudinal studies as studies with a retrospective cross-sectional design are much more common in the scientific literature on factors underlying antisocial behavior. Following participants over time, keeping track of antisocial behaviors, and assessing a variety of factors that may protect against antisocial behaviors are costly research activities, and thus only a limited number of longitudinal studies have appeared.

Third, studies examining samples of participants aged between 12 and 30 were included. Studies reporting on children younger than 12 were excluded as antisocial behavior manifests itself differently in (very) young children (Loeber, 1990). Moreover, factors underlying antisocial behavior in children may be different from factors explaining antisocial behavior in adolescents and young adults (Loeber, 1990; Van der Put et al., 2011a, 2011b). The maximum age was set to 30 in order to draw inferences on the impact of protective factors for antisocial behavior in specifically adolescents and young adults.

Fourth, studies had to be published in peer-reviewed scientific journals or be (part of) a dissertation that was accessible to the authors of this review. Unpublished studies were not included as these are far more difficult to locate, and because published studies have survived some form of a refereeing and editing process (Dunkin, 1996). Although often not peer-reviewed, dissertations were included, as these are subject to quality control in the form of supervising committees, and thus had an aspect of critical academic evaluation beyond the work of the authors (Merell et al., 2008).

Fifth, given that protective factors may be very different in prevalence and nature across cultural settings, only studies that were performed in Western countries (i.e., European countries, Australia, New Zealand, Canada, and the US) were included in the current meta-analyses. All primary studies had to be written in English or Dutch.

Sixth, primary studies had to report on (1) a measure of a bivariate association between a (putative) protective factor and a form of antisocial behavior (e.g., a correlation coefficient), or (2) sufficient statistical information for calculating such an association. Studies reporting adjusted effect sizes only were excluded, as combining and comparing differentially adjusted effect sizes may limit a robust estimation of the true impact of protective factors.

Finally, no effects of potentially protective factors from studies examining treatment effects were extracted, as the aim was not to perform a meta-analysis of the effectiveness of programs for antisocial behaviors, and because treatment effects may influence the impact of protective factors. Treatment motivation was also excluded from this review.

### Search Strategy

**Fig. 1:**
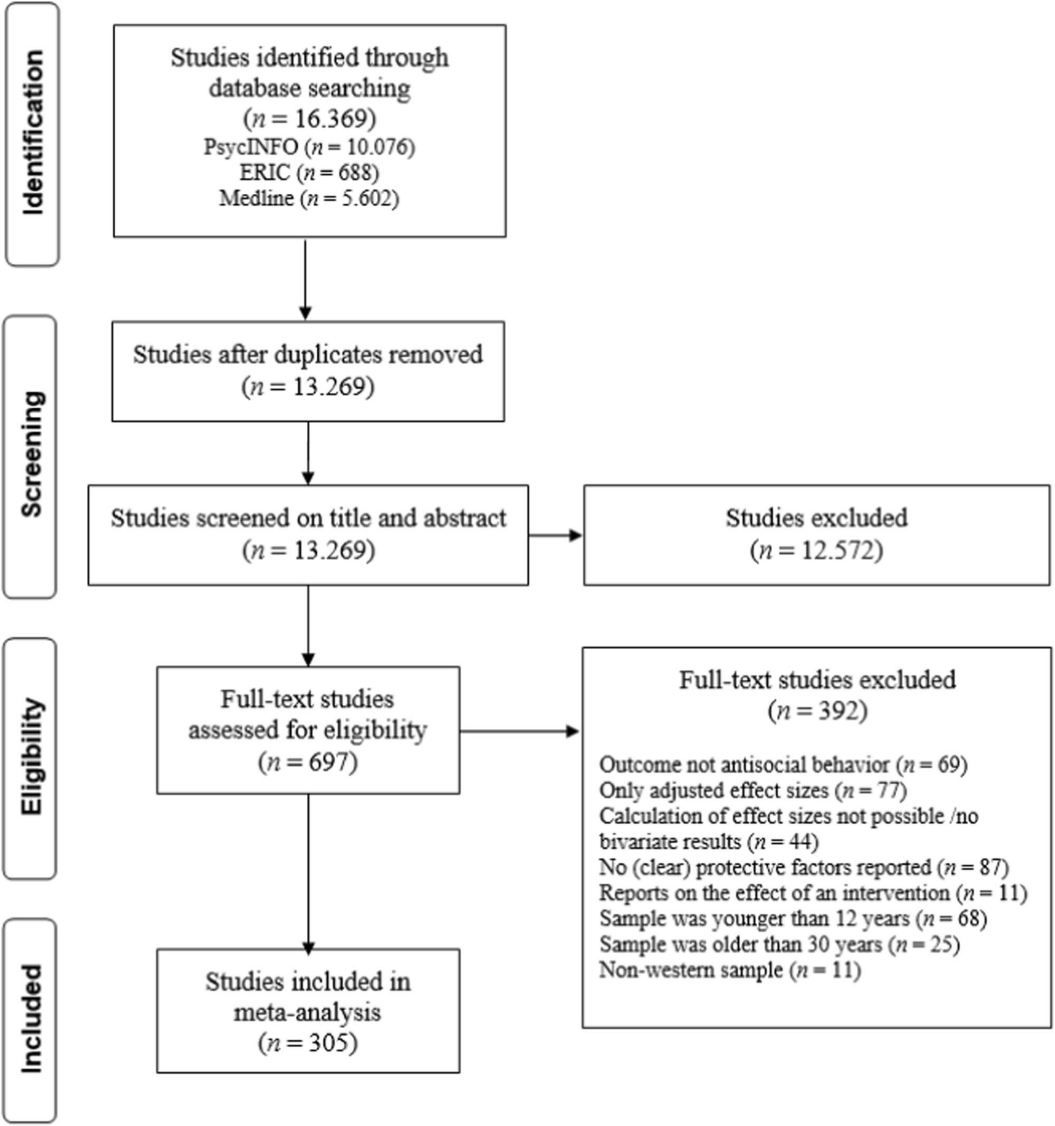
Flow Chart of Search Results

To identify relevant primary studies, an electronic search was performed in the databases PsycINFO, ERIC, and Medline. For the full search strategy of this electronic search, see Appendix S1 in the online Supporting Material. Additionally, the reference sections of all included primary studies were screened. The search strategy initially yielded 13,269 potentially relevant studies. After the exclusion of studies based on their title and/or abstract, 697 studies remained of which the full text was evaluated. Finally, 305 studies met all inclusion criteria and were included. Figure 1 presents a flow chart of the primary study search. An overview of the included studies and their characteristics are presented in Appendices S2 and S3, respectively.

### Study Coding

In coding each included primary study, a coding system was applied that was based on coding guidelines of Lipsey and Wilson (2001). The primary interest was to synthesize all available empirical evidence on variables that have been tested as protective factors for antisocial behavior of youth. However, the vast number of effect sizes that could be extracted from all included studies, made it impossible to examine each protective factor individually. For valid and intelligible analyses, each individual protective factor was therefore classified into one of multiple “domains” of protective factors, which can be defined as a category of protective factors that are (more or less) similar in nature. In this way, the procedure of previous meta-analyses that examined “risk domains” for different outcomes was followed (e.g., Assink et al., 2019; Gubbels et al., 2019).

The ecological model of Bronfenbrenner (1979, 1986) served as a guidance for the categorization of all (putative) protective factors into (putative) protective domains. As Bronfenbrenner assumed that factors in more proximal systems exert more influence on development and behavior than factors in more distal systems, the protective factors were categorized into individual-related domains and domains of factors present in the microsystems directly surrounding the individual. Specifically, the protective domains created based on all extracted (potential) protective factors were related to (1) characteristics of the individual; (2) characteristics of (the relationship with) parents and/or the family; (3) characteristics of (the relationship with) significant others outside the family (i.e., peers, romantic partners, important adults); (4) characteristics of the community or neighborhood in which an individual resides; and (5) school characteristics. For each extracted factor, it was first determined whether the factor was related to the individual, the family, others outside the family, the community, or the school. Next, a factor was further classified into more specific protective domains. This procedure followed the strategy of previous reviews on risk factors, for instance for victimization of child sexual abuse (Assink et al., 2019) and school absenteeism and dropout (Gubbels et al., 2019). All protective domains, and examples of protective factors classified in each of these domains, are listed in Table 1. In the end, (potentially) protective factors for antisocial behavior were classified into one of 77 mutually exclusive protective domains, of which 31 were related to individual characteristics, 22 to family characteristics, 8 to non-family characteristics, 7 to community characteristics, and 9 to school characteristics.

**Table 1:** Overview of Potentially Protective Domains with Examples of Factors Classified in each Domain

Protective domains (#77)^a^	Examples of protective factors classified in each domain
*Individual domains (#31)*	
Agreeableness	Having behavioral characteristics that are perceived as kind, sympathetic, cooperative, warm, and considerate; Being generous; Individual’s altruism; Being optimistic.
Autonomy or independence	Being independent in activities of daily living; Individual sophistication and maturity; One’s need for autonomy.
Capacity to reflect/mentalize	Individual’s capacity to mentalize; The ability to take another perspective; Being able to voluntarily inhibit, activate, or change attention and behavior in response to the environment:
Concentration skills	Being able to focus one’s attention on a problem or task; Low levels of ADHD symptoms; Individual’s ability to shift attention between different objects or levels of focus
Conscientiousness	Being more conscientious (i.e., valuing competence, order, dutifulness, achievement striving, self-discipline, and deliberation).
Conservativeness	Valuing tradition, conformity, and security.
Coping skills	Individual is able to effectively cope with stressful life events; Having problem-solving coping skills (i.e., research solutions to problems, generate multiple approaches to a problem, use rational decision making, and evaluate decision on the outcome); Using active or problem-focused coping skills (i.e., psychological or behavioral coping efforts that are characterized by an attempt to use one’s own resources to deal with a problem situation); Individual uses support seeking coping
Emotional intelligence	Having high levels of emotional intelligence; Individual tends to observe and think about his/her feelings and mood; The understanding of one’s emotional states.
Empathy	Feelings of empathy; Individual’s other-oriented feelings of compassion and concern
Engagement in positive leisure activities	Being engaged in extracurricular activities, organized sport activities, recreational activities, or community based organizes activities.
Ethnic socialization	Feeling more connected with one’s culture, race, or ethnicity; Individual identifies with culture, race, or ethnicity; High levels of acculturation.
Executive functions	Individual has good executive functions, including inhibition (the ability to inhibit or control impulsive responses and create responses by using attention and reasoning), planning (the ability to create a plan or a roadmap to reach a goal), and decision-making (choosing between options that have some probability of success).
Financial stability	Having skills for getting a job; Individual has feelings of financial responsibility; Owning or renting a house or accommodation; Individual has an income from a full- or part-time job; Being able to financially manage a household; Being employed or attending education.
Hopefulness	Being hopeful; Hoping for the best; Knowing things will be okay
Intelligence	Having a high (verbal and/or non-verbal) IQ; Individual has good verbal skills or high verbal intelligence; Having good cognitive abilities; Having high levels of knowledge and skills.
Life satisfaction	Being satisfied whit own life (on several dimensions, including leisure, finances, health, etc.); Individual’s well-being (Cantril’s ladder); Sense of belonging; Happiness;
Mindfulness	Mindfulness in terms of 5 dimensions: observing (the ability to perceive and recognize stimuli in one’s environment and inner experiences), describing (the ability to characterize their thoughts and emotions), act with awareness (the ability to make thoughtful and calculated decisions rather than automaticity of behavior without conscious thought), nonjudging (the ability to affirm one’s emotions and thoughts while not evaluating them as right or wrong), and nonreactivity (the ability to not react to emotionally inducing stimuli).
Morality	Having moral fortitude; Having a moral character
Physical attractiveness	Having an attractive physical appearance; Individual has an athletic physical appearance.
Physical health	Good quality of sleep; High levels of exercise or physical activity; High levels of healthy nutrition; Low levels of stress; Individual’s ability to deal with stress (i.e., stress reactivity);
Positive future aspirations	Having positive future expectations (e.g., graduating from college, finding a good job, to be happy, etc.); Individual see him/herself fulfilling life-goals; Envisioning life to be meaningful.
Prosocial values or behaviors	Individual’s negative attitude towards delinquency or aggression; Believing that physical aggression to solve a conflict is never appropriate; Showing caring behaviors; Individual shows behaviors that facilitate positive interpersonal interaction; Believing that responsible law abiding behavior apply to you.
Religiousness	Being (more) religious; Being spiritual; Individual attends religious/spiritual services; Individual has religious/spiritual believes/orientation; Religion/spirituality is important in individual’s life.
Resilience	Being capable to adapt to changing environments and life circumstances; Individual’s ability to use trust, autonomy, and initiative in handling life stressors; The ability to cope with psychologically stressful situations
Responsibility	Making responsible choices in life
Self-control	Higher levels of emotion regulation; Individual’s activation control (i.e., the capacity to perform an action when there is a tendency to avoid it); Individual’s attention control (the capacity to focus attention as well as to shift attention when desired); Individual’s inhibitory control (the capacity to plan and to suppress inappropriate responses); The ability to resist or avoid high-risk behaviors in the face of temptation.
Self-disclosure	Being more open (i.e., individual reveals personal and private information about himself as he communicates with or relates to others).
Self-esteem	Individual has high levels of self-esteem, confidence, self-compassion, self-worth, and self-efficacy; Liking yourself the way you are; Feelinf loved and wanted; Individual believes in him/herself.
Self-transcendence	Valuing the welfare of others; Individual values universalism and benevolence.
Social skills	High levels of social competence; The ability to communicate and cooperate with others; Good social-emotional functioning
Trustworthiness	Being trustworthy, integer, honest, or accountable.
*Family domains (#22)*	
Cultural socialization	Parental engagement in race/culture/ethnicity related behaviors; Frequency and range of familial behaviors and communications to children around issues of race, culture or ethnicity
Family cohesion	Commitment and support from family members; Family support; Family connectedness; Familism; Involvement in the family; Family attachment; Quality of family interaction
Family organization	Hours spent on housework or sibling care; There are roles in the family; The family emphasizes clear organization.
Family safety	Parental emotional security; Parental emotional validation; Family stability; Family expressiveness (i.e., the extent to which family members are encouraged to express their feelings directly)
Family SES	Family’s socioeconomical status; Parental education; Parental income;
Family structure	Small family size; Living with a mother and a father; Parents are married
Father involvement	Father’s presence; Father in household; Father’s involvement
Mother involvement	Maternal presence; Maternal involvement; Mother in household
Parental control	High levels of parental control or supervision in the family; Parental limits on child’ behaviors and inquiry about child’s activities and whereabouts.
Parental discipline and management	Parents are persistent in day to day discipline; Consistent parental discipline; Low levels of harsh parenting
Parental emotion regulation	Parental emotion regulation
Parental expectations of child	Parental expectations for school completion; Parental school expectation
Parental involvement	Parents spend time with their children (mother and father, or not specified); Family involvement; Parental presence; Parents are engaged in meaningful activities with child
Parental mental health	Emotional stability of the mother; Low levels of parental stress
Parental prosocial values/behaviors	Parents’ prosocial reactions to various types of misconduct; Well-socialized mother; Parental prosocial norms about violence
Parental religiousness	Importance of religion for parents; Parents attending religious services; Moral-religious emphasis in family
Positive parenting	Positive parenting practices; Nurturant parenting; Proactive parenting
Quality of family-communication	The quality of communication within the family; Conversation orientation deployed by parents
Quality of relationship with father	Closeness to father; Bond with father; Father’s social and emotional support; Paternal attachment; Paternal warmth; Supportive relationship with father
Quality of relationship with mother	Attachment to mother; Maternal caring; Maternal acceptance; Mother’s social and emotional support; Closeness to mother; Maternal warmth and sensitivity; Maternal bonding
Quality of relationship with parents	Good relationship with parents (mother and father, or not specified); Parental warmth and acceptance; Quality of bonds with parents; Connectedness with parents; Parental support; Closeness to caregiver
Quality of relationship with siblings	Sibling closeness; Good relationship with sister or brother
*Non-family domains (#8)*	
Having a romantic relationship	Involvement in a romantic relationship; Satisfaction with romantic relationship; Individuals is living with a partner; Instrumental and emotional support provided romantic partner; Partner’s spirituality
Peer attachment	Friends’ warmth; Acceptance from peers; Closeness to peers; Attachment to peers
Peer support	Friend support; Support from close friends; Peer role models; Friend caring; Emotional support from peers
Peer’s academic achievement	Friend’s academic achievement (based on Grade Point Average, GPA)
Popularity	Peer-nominated popularity; Peer social acceptance
Prosocial peers	Individual’s perceptions of peer norms about antisocial behavior; Peer emotion regulation; Low levels of peer delinquency; Peer disapproval of antisocial behavior; Low levels of peer substance use
Quality of relationship with peers	Good relationship with friends; Friendship quality; Good communication with friends
Relationship with adults	Quality of support by other adults than parents; Number of close adults in life; Social support from nonparental adults; Nonparental adult role models; Parole officers’ support; Popularity with adults; Connection to a caring adult
*Community domains (#7)*	
Collective efficacy	Neighborhood collective efficacy (i.e., neighbors agree on what is acceptable behavior and reinforce it in each other); Informal social control (i.e., neighbors’ willingness to intervene in scenarios such as “a fight breaking out in front of the house”)
Community involvement	Involvement in community; Opportunities for prosocial involvement; Recognition for prosocial involvement; Civic engagement; Neighborliness
Neighborhood attachment	High level of attachment to one’s neighborhood; Neighborhood connectedness (i.e., caring about neighborhood, how much individuals liked their neighborhood, and the extent to which people in their neighborhoods cared about them); Neighborhood satisfaction
Neighborhood cohesion	High level of neighborhood trust and cooperation; Looking out for each other; Willingness of help neighbors; Social neighborhood cohesion
Neighborhood quality	Positive influences from neighborhood; Good neighborhood organization; Neighborhood derivation from crime and substance abuse; The quality of schools and the types of businesses; Neighborhood greenness; Urbanization of neighborhood; Prosocial neighborhood opportunities
Neighborhood safety	High levels of neighborhood safety (i.e., safe to leave windows and doors open during the day, go out alone at night, walk alone in daylight at a park, and talk to strangers in the community, etc.); Local law enforcement; No drugs and gun available in neighborhood
Social support	Social support in general; Practical social support; Large social network; Number of positive and material supporters; Environmental resources; Access to social support
*School domains (#9)*^b^	
Positive attitude towards school	Positive orientation toward school; School satisfaction
Positive class or school climate	Positive school climate; Order and organization in classroom; Rules in classroom are clear; School safety; Perceived school multiculturalism
School achievement	High grade-point average; High grades (e.g., in reading or math); School performance; Number of hours spent on homework; Academic competence and importance
School attendance	Months of education; School or college attendance
School involvement	Degree of student attentiveness, interest and participation in class activities;
School self-esteem	Feeling intelligent and competent in academic pursuit; Academic self-efficacy
Student-teacher relationship	The amount of help, trust and friendship the teacher offers to students; Support from teacher; Teacher emotional and social support; Closeness to teacher.
Positive attitude towards school	Positive orientation toward school; School satisfaction
Positive class or school climate	Positive school climate; Order and organization in classroom; Rules in classroom are clear; School safety; Perceived school multiculturalism

^a^Except otherwise indicated, the labels of the protective domains have a positive direction (i.e., “Higher levels of…”)

^b^Most school domains apply to school-attending youth

For descriptive purposes, several study, sample and outcome characteristics were coded. It was decided to conduct moderator analyses for two sample characteristics and one outcome characteristic, which were the average age of the sampled participants (at time of the antisocial behavior measurement), the percentage of males in primary study samples, and the type of antisocial behavior that was categorized into less severe antisocial behavior, such as behavioral problems, externalizing behavior, and rule-breaking behavior, versus more severe antisocial behavior, such as delinquency, assault, and violence. Previous studies have revealed substantial age, gender, and behavioral sub-type differences in the (impact of) underlying factors explaining antisocial behavior (Funk, 1999; Loeber, 1990; Liu & Miller, 2020; Schwalbe et al., 2006; Van der Put et al., 2011a, 2011b). Therefore, specifically these three variables were considered important to test as a moderator of the overall impact of protective domains. In coding studies for meta-analytic research, it is common practice to retrieve a large amount of information from primary studies (see for instance, Cooper et al. (2019); Lipsey and Wilson (2001)), after which the moderating effect of a variety of study, sample, and research design descriptors is tested. However, since the problem of multiple testing often dealt with in primary studies (see, for instance, Tabachnik & Fidell, 2013) is equally present in a meta-analysis, it was decided to code the variables that seemed most relevant in the present review. Further, for sufficient statistical power in the moderator analyses, the three variables were only tested as a moderator when these variables could be coded for at least five studies (per category) within a protective domain.

In coding all included studies, two coding rounds were completed. First, 20 studies (reporting on 249 effect sizes) that were eligible for inclusion were randomly selected and coded by the first author and an independent assistant researcher. Next, the independent codings were compared and percentages of agreement were calculated. A perfect agreement (100%) was found for the percentage of males in the sample and the double-coded effect sizes. The agreement for the average age of participants in study samples was 95%, and the agreement for the number of extracted effect sizes was 90%. All discrepancies in the non-matching codings were discussed by the two coders until full consensus was reached. In the second coding round, the first author coded the remaining 284 studies. The second and third authors were consulted regularly to discuss complex coding issues. These issues were resolved by discussion so that all authors fully agreed on all final coding decisions. Finally, the classification of each extracted potentially protective factor into a single protective domain was discussed by all study authors, so that all authors agreed on each classification.

### Calculation of Effect Sizes

As common effect size for the impact of protective factors, the correlation coefficient (*r*) was calculated for each extracted (potential) protective factor. The correlation coefficient was chosen as this effect size is a common measure for capturing the impact of risk factors in quantitative syntheses on risk factors for different outcomes (see, for example, Assink et al., 2019; Gubbels et al., 2019; Mulder et al., 2018; Wissink et al., 2023). The correlations were directly obtained from the included studies, or calculated using information reported in primary studies (such as proportions, means and standard deviations, odds-ratio’s, or *F* or *t* values). In these calculations, the formulas of Ferguson (1966), Rosenthal (1994), and Lipsey and Wilson (2001) were used. It was essential that each correlation coefficient reflected the association between a (putative) protective factor and antisocial behavior of youth in the proper direction. Therefore, when higher levels of a protective factor (e.g., higher levels of empathy, family cohesion, or school achievement) was associated with lower levels of antisocial behavior, a negative sign was given to an *r* value. A positive sign was given to *r* values when higher levels of protective factors were associated with higher levels of antisocial behavior. After all correlation coefficients were obtained, the *r* values were transformed into Fishers *z* values, as correlations are non-normally distributed (see, for instance, Lipsey & Wilson, 2001). After the analyses were performed, the Fisher’s *z* values were transformed back into correlations for ease of interpretability. The guidelines of Funder and Ozer (2019) were used to interpret the magnitude of the effect sizes, with effect sizes of *r* ≥ −0.30 considered as large, *r* ≥ −0.20 as medium, *r* ≥ −0.10 as small, and *r* ≥ −0.05 as very small.

### Statistical Analyses

The analytic strategy was based on previous reviews in which the impact of (putative) risk factors for persistent delinquent behavior (Assink et al., 2015), victimization of child sexual abuse (Assink et al., 2019), and school absenteeism and dropout (Gubbels et al., 2019) were classified and synthesized. Central to this strategy was the application of a multilevel approach to meta-analysis. Specifically, because most primary studies that were eligible for inclusion reported on more than one (potential) protective factor for antisocial behavior, a traditional random effects (two-level) model was extended to a three-level random effects model (Cheung, 2014; Houben et al., 2015; Van den Noortgate et al., 2013; 2014). A major advantage of this three-level approach to meta-analysis is that all relevant effects reported in each primary study can be included, implying that all relevant information is preserved. As a result, no information is lost and (moderator) effects can be estimated more precisely and with maximum power in the statistical analyses (Assink & Wibbelink, 2016). In a three-level random effects meta-analytic model, three sources of variance were taken into account to deal with effect size dependency: sampling variance of the observed effect sizes (Level 1), variance between effect sizes extracted from the same study (Level 2), and variance between studies (Level 3). The overall impact of each protective domain was estimated in an intercept-only model, in which the intercept represents the estimated overall impact. If variation in effect sizes extracted from the same study (i.e., level 2 variance) and/or variation in effect sizes extracted from different studies (i.e., level 3 variance) was significant within a protective domain (i.e., there was effect size heterogeneity within a protective domain), the three-level intercept-only model was extended with the coded variables mean age, percentage of males in the sample, and subtype of antisocial behavior to determine whether these variables can significantly explain variance in effect sizes and thus moderate the impact of a protective domain. These moderator analyses were performed separately for age, percentage, and behavioral subtype and only performed when these variables were based on at least five studies (per category).

In the statistical environment R (version 3.5.1; R Core Team, 2015), the function “rma.mv” of the metafor-package (Viechtbauer, 2010) was used to conduct the statistical analyses. The R syntaxes were written so that the three sources of variance were modeled (Assink & Wibbelink, 2016). In testing individual regression coefficients and calculating corresponding confidence intervals, a *t*-distribution was used (Knapp & Hartung, 2003). To determine the significance of the level 2 and level 3 variance, the full model was compared to a model excluding one of these variance parameters in two separate log-likelihood ratio tests. If significant level-2 and/or level-3 variance was detected, the distribution of effect sizes was considered to be heterogeneous. This indicated that effect sizes could not be treated as estimates of one common effect size, and that moderator analyses could be performed. All model parameters were estimated using the restricted maximum likelihood estimation method. As the variables mean age and percentage of males in the sample are both continuous, these variables were centered around their mean prior to conducting the moderator analyses. The log-likelihood-ratio-tests were performed one-tailed and all other tests were performed two-tailed. A *p* value < 0.05 was considered statistically significant. For brevity, only the significant results of the moderator analyses were reported. The insignificant results are available upon request from the first author.

### Assessment of Bias

Despite an extensive search for primary studies that tested variables as protective factors for antisocial behavior and the large number of studies that could be included, it is possible that relevant studies were missed due to limitations in the current search strategy or different forms of bias, such as publication bias or subjective reporting bias. In examining whether (a form of) bias was present in the effect sizes analyzed, two analyses were conducted. First, the funnel-plot-based trim and fill method was conducted (Duval & Tweedie 2000a, 2000b) in which effect sizes are plotted against their standard errors. In case of an asymmetrical distribution of effect sizes (i.e., an asymmetrical funnel plot), the trim-and fill method restores symmetry of the distribution by imputing effect size estimates from “missing” studies. Effect sizes can be imputed either in the left or right side of the funnel plot, depending on whether below average or above average effect sizes are underrepresented in the data. Second, a three-level Egger’s test was conducted in which effect sizes were regressed on standard errors in a 3-level meta-analytic model. In this test that accounts for effect size dependency, a significant slope is an indication of bias. These bias assessment analyses were also performed in the R environment (Version 3.5.1; R Core Team 2015) with the functions “trimfill” and “rma.mv” of the metafor package (Viechtbauer, 2010).

## Results

In total, *k* = 305 studies published between 1995 and 2023 were included (*N* = 494,638 participants). The average sample size was 1228 (*SD* = 4,060.84) and ranged from *n* = 27 to *n* = 105,573. A total of *k* = 275 articles published in a peer-reviewed scientific journals and *k* = 30 dissertations were included. The average percentage of males across the samples of these studies was 52.7%, and the mean age of the participants when the antisocial behavior was measured was 16.31 years (*SD* = 3.30). According to the classification of antisocial behavior into severe and less severe forms, more studies reported on less severe forms of antisocial behavior (*k* = 195; e.g., behavioral problems, externalizing behavior, rule-breaking behavior) than on more severe forms (*k* = 141; e.g., delinquency, assault, violence). The included studies were conducted in North-America (i.e., USA or Canada; *k* = 232), Europe (*k* = 56), New-Zealand/Australia (*k* = 4), and in a combination of these continents (*k* = 13). In total, 1,850 effect sizes could be retrieved from all primary studies, each reflecting the effect of a putative protective factor for antisocial behavior. The mean number of extracted effect sizes per study was 6.08 (*SD* = 5.89).

### Overall Impact of Protective Domains

Table 2 presents an estimated overall effect for each of the 77 (potentially) protective domains for antisocial behavior in descending order of effect size magnitude, and separately for individual-, family-, non-family-, community, and school related domains. The overall effects of 50 domains were significant and in a negative direction (including 24 individual domains, 12 family domains, 5 non-family domains, 3 community domains, and 6 school domains), implying that these domains can be regarded as true protective domains for antisocial behavior of youth. The effect of these protective domains ranged from very small (i.e., *r* = −0.042 for “family structure”) to large (i.e., *r* = −0.396 for “conservativeness”). According to the guidelines of Funder and Ozer (2019), significant medium (*r* ≥ −0.20) and large impact (*r* ≥ −0.30) was found for 13 protective domains (indicated in Table 2 with “^b^” for large effects and “^c^” for medium effects), which are (decreasing in impact): “conservativeness”, “self-transcendence”, “life satisfaction”, “having a romantic relationship”, “capacity to reflect or mentalize”, “quality of relationship with peers”, “prosocial peers”, “prosocial values or behaviors”, “agreeableness”, “school self-esteem”, “parental control”, “resilience”, and “social skills”. Further, multiple protective domains were identified with a significant small and very small overall effect (indicated in Table 2 with “^d^” and “^e^”, respectively).

**Table 2:** Overall Impact of 77 Potentially Protective Domains

Domain of protective factors^a^	# Studies	# ES	Mean *z* (SE)	95% CI	Sig. mean *z* (*p*)	Mean *r*	% Var. at level 1	Level 2 variance	% Var. at level 2	Level 3 variance	% Var. at level 3
**Individual domains (#31)**											
Conservativeness	1	6	−0.419 (0.025)	(−0.482, −0.355)	<0.001^***^	−0.396^b^	87.1	0.000	12.9	0.000	0.0
Self-transcendence	1	6	−0.404 (0.032)	(−0.488, −0.321)	<0.001^***^	−0.383^b^	51.2	0.003	48.8	0.000	0.0
Trustworthiness	2	2	−0.328 (0.085)	(−1.406, 0.751)	0.161	−0.317	11.2	0.006	44.4	0.006	44.4
Life satisfaction	5	10	−0.286 (0.062)	(−0.426, −0.147)	0.001^**^	−0.278^c^	15.3	0.000	0.0	0.017^**^	84.7
Capacity to reflect/mentalize	5	11	−0.253 (0.032)	(−0.325, −0.181)	<0.001^***^	−0.248^c^	33.6	0.007^***^	66.4	0.000	0.0
Prosocial values or behaviors	25	55	−0.229 (0.031)	(−0.290, −0.167)	<0.001^***^	−0.225^c^	3.3	0.004^***^	16.0	0.019^***^	80.7
Hopefulness	2	2	−0.226 (0.027)	(−0.566, 0.114)	0.075^+^	−0.222	100	0.000	0.0	0.000	0.0
Agreeableness	5	10	−0.217 (0.070)	(−0.376, −0.059)	0.013^*^	−0.214^c^	6.7	0.008^***^	31.5	0.016^+^	61.8
Resilience	13	44	−0.205 (0,067)	(−0.340, −0.071)	0.004^**^	−0.202^c^	1.3	0.008^***^	13.6	0.048^***^	85.0
Social skills	14	24	−0.203 (0.040)	(−0.286, −0.120)	<0.001^***^	−0.200^c^	5.6	0.029^***^	86.6	0.003	7.8
Positive future aspirations	12	17	−0.199 (0.022)	(−0.245, −0.152)	<0.001^***^	−0.196^d^	13.9	0.005^***^	79.0	0.000	7.2
Morality	3	3	−0.196 (0.041)	(−0.373, −0.019)	0.041^*^	−0.194^d^	37.0	0.001	31.5	0.001	31.5
Financial stability	4	4	−0.194 (0.115)	(−0.561, 0.172)	0.190	−0.192	2.9	0.026	48.6	0.026	48.6
Responsibility	1	2	−0.192 (0.025)	(−0.507, 0.123)	0.082^+^	−0.190	74.7	0.000	25.3	0.000	0.0
Self-control	29	65	−0.187 (0.023)	(−0.232, −0.141)	<0.001^***^	−0.185^d^	5.8	0.009^***^	47.7	0.008^***^	46.5
Self-disclosure	3	7	−0.180 (0.038)	(−0.273, −0.087)	0.003^**^	−0.178^d^	48.0	0.002	24.3	0.002	27.7
Emotional intelligence	6	22	−0.176 (0.068)	(−0.318, −0.035)	0.017^*^	−0.174^d^	9.7	0.002^*^	7.4	0.020^***^	82.9
Empathy	9	25	−0.158 (0.051)	(−0.262, −0.053)	0.005^**^	−0.157^d^	4.0	0.022^***^	62.2	0.012^*^	33.8
*Shyness*	1	1	−0.146 (0.048)	(−0.233, −0.058)	0.001^**^	−0.145^d^	-	-	-	-	-
*Low levels of testosterone*	1	1	−0.141 (0.061)	(−0.260, −0.022)	0.020^*^	−0.140^d^	-	-	-	-	-
Coping skills	16	59	−0.137 (0.033)	(−0.203, −0.070)	<0.001^***^	−0.136^d^	12.8	0.001^+^	3.9	0.015^***^	83.3
Self-esteem	29	58	−0.128 (0.026)	(−0.179, −0.077)	<0.001^***^	−0.127^d^	6.7	0.004^***^	22.9	0.013^***^	70.4
Conscientiousness	3	7	−0.124 (0.039)	(−0.219, −0.029)	0.019^*^	−0.123^d^	51.9	0.000	5.8	0.003	42.3
Concentration skills	5	15	−0.121 (0.095)	(−0.324, 0.082)	0.223	−0.120	3.6	0.025^***^	44.0	0.030	52.5
Intelligence	14	26	−0.112 (0.032)	(−0.178, −0.045)	0.002^**^	−0.112^d^	9.7	0.001	4.0	0.012^***^	86.3
*Persistence*	1	1	−0.105 (0.031)	(−0.166, −0.043)	<0.001^***^	−0.105^d^	-	-	-	-	-
Engagement in positive leisure activities	23	68	−0.097 (0.030)	(−0.157, −0.037)	0.002^**^	−0.097^e^	7.7	0.001^*^	5.7	0.019^***^	86.6
Religiousness	31	67	−0.093 (0.019)	(−0.130, −0.055)	<0.001^***^	−0.093^e^	4.5	0.004^***^	37.4	0.006^***^	58.1
Executive functions	2	17	−0.092 (0.025)	(−0.144, −0.040)	0.002^**^	−0.092^e^	45.8	0.000	7.7	0.001^+^	46.5
Mindfulness	1	20	−0.090 (0.019)	(−0.130, −0.051)	<0.001^***^	−0.090^e^	37.7	0.004^***^	62.3	0.000	0.0
Autonomy or independence	3	16	−0.087 (0,146)	(−0.397, 0.223)	0.559	−0.087	6.2	0.000	0.0	0.062^***^	93.8
Ethnic socialization	19	66	−0.076 (0.036)	(−0.148, −0.004)	0.040^*^	−0.076^e^	9.7	0.010^***^	32.7	0.017^***^	57.6
Physical health	8	16	−0.062 (0.037)	(−0.142, 0.017)	0.117	−0.062	9.3	0.004^*^	29.7	0.008^*^	60.9
Physical attractiveness	4	5	−0.052 (0.007)	(−0.072, −0.033)	0.002^**^	−0.052^e^	100.0	0.000	0.0	0.000	0.0
**Family domains (#22)**											
*Having an older mother*	1	1	−0.290 (0.045)	(−0.377, 0.202)	<0.001^***^	−0.282^c^	-	-	-	-	-
Parental control	36	94	−0.207 (0.024)	(−0.253, −0.160)	<0.001^***^	−0.204^c^	4.2	0.004^***^	18.0	0.016^***^	77.8
Family organization	5	11	−0.190 (0.030)	(−0.256, −0.124)	<0.001^***^	−0.188^d^	16.2	0.008^***^	83.8	0.000	0.0
Family cohesion	43	105	−0.179 (0.018)	(−0.214, −0.144)	<0.001^***^	−0.177^d^	5.9	0.010^***^	59.8	0.006^***^	34.3
Quality of relationship with mother	43	105	−0.179 (0.018)	(−0.214, −0.144)	<0.001^***^	−0.177^d^	5.9	0.010^***^	59.8	0.006^***^	34.3
Quality of family-communication	4	13	−0.177 (0.023)	(−0.228, −0.127)	<0.001^***^	−0.175^d^	19.6	0.005^***^	80.4	0.000	0.0
Quality of relationship with parents	67	156	−0.163 (0.014)	(−0.191, −0.135)	<0.001^***^	−0.162^d^	4.9	0.005^***^	34.5	0.009^***^	59.6
Parental involvement	20	29	−0.151 (0.023)	(−0.199, −0.104)	<0.001^***^	−0.150^d^	5.5	0.013^***^	89.8	0.001	4.8
Quality of relationship with siblings	2	4	−0.142 (0.098)	(−0.454, 0.169)	0.242	−0.141	6.8	0.031	87.6	0.002	5.5
Family safety	6	20	−0.140 (0.021)	(−0.183, −0.097)	<0.001^***^	−0.139^d^	68.3	0.000	0.0	0.001	31.7
Family SES	3	3	−0.120 (0.064)	(−0.397, 0.157)	0.204	−0.119	11.7	0.005	44.2	0.005	44.2
Father involvement	3	7	−0.116 (0.036)	(−0.206, −0.027)	0.019^*^	−0.115^d^	19.7	0.000	0.0	0.004^*^	80.3
Parental prosocial values/behaviors	6	15	−0.104 (0.077)	(−0.269, 0.061)	0.197	−0.104	8.5	0.004^+^	12.0	0.030^*^	79.5
Parental emotion regulation	2	3	−0.101 (0.043)	(−0.286, 0.085)	0.144	−0.101	87.9	0.000	0.0	0.001	12.1
Quality of relationship with father	15	22	−0.100 (0.024)	(−0.149, −0.051)	<0.001^***^	−0.100^d^	11.8	0.000	0.0	0.006^**^	88.2
Mother involvement	5	10	−0.094 (0.040)	(−0.184, −0.004)	0.043^*^	−0.094^e^	12.9	0.003	27.9	0.005	59.1
Parental expectations of child	2	2	−0.093 (0.015)	(−0.284, 0.099)	0.102	−0.093	64.8	0.000	17.6	0.000	17.6
Parental mental health	2	10	−0.082 (0.042)	(−0.176, 0.013)	0.081^+^	−0.082	30.6	0.012^**^	69.4	0.000	0.0
Parental discipline and management	11	16	−0.078 (0.044)	(−0.171, 0.015)	0.095^+^	−0.078	2.6	0.003^***^	12.5	0.018^*^	84.9
Positive parenting	7	12	−0.043 (0.057)	(−0.170, 0.083)	0.464	−0.043	14.5	0.000	0.0	0.018^***^	85.6
Family structure	3	7	−0.042 (0.012)	(−0.072, −0.012)	0.014^*^	−0.042^e^	56.4	0.000	0.0	0.000	43.6
Parental religiousness	2	6	−0.040 (0.038)	(−0.137, 0.058)	0.340	−0.040	41.4	0.005^*^	58.5	0.000	0.0
Cultural socialization	4	13	−0.003 (0.047)	(−0.105, 0.099)	0.956	−0.003	43.3	0.000	0.0	0.007^+^	56.7
**Non-family domains (#8)**											
Having a romantic relationship	4	10	−0.260 (0.085)	(−0.453, −0.068)	0.014^*^	−0.254^c^	10.6	0.065^***^	89.4	0.000	0.0
Quality of relationship with peers	5	16	−0.250 (0.075)	(−0.411, −0.090)	0.005^**^	−0.245^c^	15.1	0.000	0.0	0.026^***^	84.9
Prosocial peers	15	25	−0.244 (0.040)	(−0.327, −0.160)	<0.001^***^	−0.239^c^	4.6	0.004^***^	14.9	0.019^**^	80.5
Peer’s academic achievement	1	2	−0.172 (0.020)	(−0.423, 0.080)	0.073^+^	−0.170	100.0	0.000	0.0	0.000	0.0
Relationship with adults	18	32	−0.156 (0.022)	(−0.202, −0.110)	<0.001^***^	−0.155^d^	15.4	0.006^***^	56.1	0.003	28.5
Peer support	18	31	−0.077 (0.036)	(−0.152, −0.003)	0.043^*^	−0.077^e^	4.1	0.004^***^	15.1	0.019^***^	80.8
Peer attachment	12	21	−0.072 (0.041)	(−0.157, 0.013)	0.093^+^	−0.072	5.4	0.003^***^	15.8	0.015^**^	78.8
Popularity	2	7	−0.054 (0.024)	(−0.114, 0.005)	0.068^+^	−0.054	100	0.000	0.0	0.000	0.0
*Peer religiousness*	1	1	−0.030 (0.007)	(−0.043, −0.017)	<0.001^***^	−0.030	-	-	-	-	-
**Community domains (#7)**											
Social support	17	23	−0.135 (0.033)	(−0.203, −0.067)	<0.001^***^	−0.134^d^	5.1	0.000	0.0	0.015^+^	94.9
Neighborhood quality	7	9	−0.109 (0.042)	(−0.206, −0.012)	0.033^*^	−0.109^d^	11.3	0.000	0.0	0.010^*^	88.7
Collective efficacy	6	10	−0.084 (0.030)	(−0.151, −0.017)	0.019^*^	−0.084^e^	9.8	0.000	2.6	0.004^*^	87.6
Neighborhood cohesion	6	6	−0.065 (0.069)	(−0.242, 0.113)	0.393	−0.065	1.0	0.014	49.5	0.014	49.5
Community involvement	3	5	−0.046 (0.053)	(−0.194, 0.103)	0.442	−0.046	4.6	0.007^***^	64.6	0.003	30.8
Neighborhood attachment	8	10	−0.034 (0.039)	(−0.123, 0.055)	0.412	−0.034	5.8	0.003^**^	27.6	0.008	66.7
Neighborhood safety	6	10	0.004 (0.020)	(−0.042, 0.050	0.850	0.004	6.9	0.003^***^	89.9	0.000	3.2
**School domains (#9)**											
School self-esteem	5	8	−0.212 (0.041)	(−0.310, −0.114)	0.001^**^	−0.209^c^	28.7	0.009^+^	69.3	0.000^*^	2.0
Positive class or school climate	6	22	−0.182 (0.030)	(−0.245, −0.120)	<0.001^***^	−0.180^d^	13.8	0.000	1.0	0.004^***^	85.2
School achievement	33	67	−0.178 (0.021)	(−0.220, −0.136)	<0.001^***^	−0.176^d^	3.1	0.005^***^	33.6	0.010^**^	63.3
School involvement	42	76	−0.160 (0.020)	(−0.200, −0.120)	<0.001^***^	−0.159^d^	2.8	0.005^***^	26.1	0.012^***^	71.1
School attendance	5	9	−0.160 (0.079)	(−0.343, 0.023)	0.079^+^	−0.159	5.9	0.003^+^	10.2	0.028^*^	83.9
Positive attitude towards school	6	13	−0.157 (0.048)	(−0.261, −0.052)	0.007^**^	−0.156^d^	10.9	0.002	10.3	0.012^+^	78.8
Student-teacher relationship	14	36	−0.153 (0.030)	(−0.214, −0.092)	<0.001^***^	−0.152^d^	6.2	0.003^***^	26.9	0.008^***^	66.8
Educational aspirations	2	4	−0.113 (0.044)	(−0.251, 0.026)	0.081^+^	−0.113	15.0	0.006^**^	85.0	0.000	0.0
Classmate support	6	13	−0.037 (0.060)	(−0.167, 0.094)	0.553	−0.037	7.3	0.001	4.4	0.019^**^	88.3

Individual factors that could not be classified into one of the domains, are presented in *italics*.

*# studies* number of studies, *# ES* number of effect sizes, *SE* standard error, *CI* confidence interval, *Sig* significance, *Mean* *z* mean effect size (*z*), *Mean* *r* the correlation coefficient corresponding to the mean effect size *z*, *% Var* percentage of variance explained, *Level 2 variance* variance between effect sizes from the same study, *Level 3 variance* variance between studies

^+^*p* < 0.10; *p < 0.05; **p < 0.01; ***p < 0.001

^a^Except otherwise indicated, the labels of the protective domains have a positive direction (i.e., “Higher levels of…”)

^b^Significant large effect (*r* ≤ −0.30; Funder & Ozer, 2019)

^c^Significant medium effect (*r* ≤ −0.20; Funder & Ozer, 2019)

^d^Significant small effect (*r* ≤ −0.10; Funder & Ozer, 2019)

^e^Significant very small effect (*r* ≤ −0.05; Funder & Ozer, 2019)

For 27 domains, the estimated overall effect did not significantly deviate from zero implying that these domains cannot be regarded as *protective* domains given the present results. Of these 27 domains, nine had a trend significant overall effect (i.e., 0.050 < *p* < 0.100) and 26 an overall effect in the negative direction. Table 2 also shows the impact of five individual protective factors (presented in italics) that could not be classified in any of the created protective domains, due to their unique nature. The correlations of these factors were all significant according to the primary studies these factors were extracted from, and medium to very small in size implying that these variables could be identified as a protective factor for antisocial behavior.

### Assessment of Bias

Table 3 presents the results of the bias assessment analyses. There was no indication of bias in 18 estimated protective domain effects (i.e., 0 out of 2 methods indicated bias), some indication of bias in 47 protective domain effects (i.e., 1 out of 2 methods indicated bias), and substantial indication of bias in 13 protective domain effects (i.e., 2 out of 2 methods indicated bias). These results show indications of bias in most of the estimated protective domains. For brevity, the funnel plots produced by the trim-and-fill analyses are not presented here, but are available upon request from the first author.

**Table 3:** Results of Two Methods for the Assessment of Bias in the Estimated Mean Impact of Potentially Protective Domains

Domain of protective factors^a^	*r*	Trim-and-fill analysis	Three-level Egger’s regression test	No. of methods indicating bias (out of 2)
**Individual domains (#31)**				
Conservativeness	−0.396	-	β_1_ = 6.776, *p* = 0.273	0
Self-transcendence	−0.383	-	β_1_ = 15.121, *p* = 0.008^**^	1
Trustworthiness	−0.317	Overestimation (1 ES missing)	β_1_ = −18.182, *p* = 0.003^**^	2
Life satisfaction	−0.278	Underestimation (2 ES missing)	β_1_ = −0.503, *p* = 0.766	1
Capacity to reflect/mentalize	−0.248	Overestimation (1 ES missing)	β_1_ = −0.216, *p* = 0.896	1
Prosocial values or behaviors	−0.225	Underestimation (6 ES missing)	β_1_ = −0.708, *p* = 0.384	1
Hopefulness	−0.222	Overestimation (1 ES missing)	β_1_ = −0.420, *p* = 0.801	1
Agreeableness	−0.214	-	β_1_ = 1.521, *p* = 0.593	0
Resilience	−0.202	Underestimation (9 ES missing)	β_1_ = −0.745, *p* = 0.650	1
Social skills	−0.200	Underestimation (7 ES missing)	β_1_ = 0.140, *p* = 0.919	1
Positive future aspirations	−0.196	-	β_1_ = −1.011, *p* = 0.373	0
Morality	−0.194	Underestimation (1 ES missing)	β_1_ = 0.699, *p* = 0.688	1
Financial stability	−0.192	Underestimation (1 ES missing)	β_1_ = −19.260, *p* = 0.003^**^	2
Responsibility	−0.190	Underestimation (1 ES missing)	NA	1
Self-control	−0.185	Underestimation (15 ES missing)	β_1_ = −0.086, *p* = 0.914	1
Self-disclosure	−0.178	-	β_1_ = 2.229, *p* = 0.839	0
Emotional intelligence	−0.174	Underestimation (1 ES missing)	β_1_ = −0.778, *p* = 0.588	1
Empathy	−0.157	Overestimation (2 ES missing)	β_1_ = 1.827, *p* = 0.454	1
Coping skills	−0.145	Overestimation (15 ES missing)	β_1_ = 1.487, *p* = 0.112	1
Self-esteem	−0.140	Overestimation (9 ES missing)	β_1_ = 0.286, *p* = 0.725	1
Conscientiousness	−0.136	-	β_1_ = −12.121, *p* = 0.016^*^	1
Concentration skills	−0.127	-	β_1_ = −1.528, *p* = 0.801	0
Intelligence	−0.123	Underestimation (3 ES missing)	β_1_ = −0.749, *p* = 0.368	1
Engagement in positive leisure activities	−0.120	Underestimation (10 ES missing)	β_1_ = −1.206, *p* = 0.258	1
Religiousness	−0.112	Underestimation (9 ES missing)	β_1_ = −0.434, *p* = 0.519	1
Executive functions	−0.097	Underestimation (5 ES missing)	β_1_ = 10.079, *p* = 0.321	1
Mindfulness	−0.093	-	NA	0
Autonomy or independence	−0.093	Underestimation (1 ES missing)	β_1_ = −5.623, *p* = <0.001^***^	2
Ethnic socialization	−0.092	Underestimation (9 ES missing)	β_1_ = −1.494, *p* = 0.212	1
Physical health	−0.090	-	β_1_ = −0.050, *p* = 0.978	0
Physical attractiveness	−0.087	Overestimation (1 ES missing)	β_1_ = 0.131, *p* = 0.857	1
**Family domains (#22)**				
Parental control	−0.204	Overestimation (24 ES missing)	β_1_ = −2.011, *p* = 0.042^*^	2
Family organization	−0.188	-	β_1_ = −0.190, *p* = 0.925	0
Family cohesion	−0.177	Underestimation (14 ES missing)	β_1_ = 0.602, *p* = 0.405	1
Quality of relationship with mother	−0.177	Underestimation (4 ES missing)	β_1_ = −1.839, *p* = 0.015^*^	2
Quality of family-communication	−0.175	Overestimation (1 ES missing)	β_1_ = −0.905, *p* = 0.255	1
Quality of relationship with parents	−0.162	Underestimation (23 ES missing)	β_1_ = −0.594, *p* = 0.229	1
Parental involvement	−0.150	-	β_1_ = −1.029, *p* = 0.470	0
Quality of relationship with siblings	−0.141	Underestimation (1 ES missing)	β_1_ = 31.809, *p* = 0.345	1
Family safety	−0.139	Overestimation (5 ES missing)	β_1_ = −1.309, *p* = 0.078^+^	1
Family SES	−0.119	-	β_1_ = −4.075, *p* = 0.018^*^	1
Father involvement	−0.115	Overestimation (1 ES missing)	β_1_ = −4.558, *p* = 0.003^**^	2
Parental prosocial values/behaviors	−0.104	-	β_1_ = −1.651, *p* = 0.651	0
Parental emotion regulation	−0.101	-	β_1_ = −20.786, *p* = 0.338	0
Quality of relationship with father	−0.100	Overestimation (3 ES missing)	β_1_ = −1.638, *p* = 0.041^*^	2
Mother involvement	−0.094	Underestimation (1 ES missing)	β_1_ = −2.670, *p* = <0.001^***^	2
Parental expectations of child	−0.093	Underestimation (1 ES missing)	β_1_ = 2.351, *p* = 0.214	1
Parental mental health	−0.082	Overestimation (2 ES missing)	β_1_ = 2.189, *p* = 0.639	1
Parental discipline and management	−0.078	Overestimation (2 ES missing)	β_1_ = −1.836, *p* = 0.412	1
Positive parenting	−0.043	-	β_1_ = −0.961, *p* = 0.575	0
Family structure	−0.042	Overestimation (2 ES missing)	β_1_ = −1.792, *p* = 0.465	1
Parental religiousness	−0.040	Underestimation (2 ES missing)	β_1_ = 1.863, *p* = 0.399	1
Cultural socialization	−0.003	Underestimation (4 ES missing)	β_1_ = 5.260, *p* = 0.009^**^	2
**Non-family domains (#8)**				
Having a romantic relationship	−0.254	Overestimation (1 ES missing)	β_1_ = 11.637, *p* = 0.384	1
Quality of relationship with peers	−0.245	Underestimation (3 ES missing)	β_1_ = 11.329, *p* = 0.014^*^	2
Prosocial peers	−0.239	Underestimation (6 ES missing)	β_1_ = 0.201, *p* = 0.869	1
Peer’s academic achievement	−0.170	Overestimation (1 ES missing)	NA	1
Relationship with adults	−0.155	Underestimation (4 ES missing)	β_1_ = −0.949, *p* = 0.146	1
Peer attachment	−0.077	Underestimation (1 ES missing)	β_1_ = −1.301, *p* = 0.297	1
Peer support	−0.072	-	β_1_ = 0.026, *p* = 0.984	0
Popularity	−0.054	-	β_1_ = −0.652, *p* = 0.745	0
**Community domains (#7)**				
Social support	−0.134	Underestimation (3 ES missing)	β_1_ = −0.159, *p* = 0.879	1
Neighborhood quality	−0.109	Underestimation (1 ES missing)	β_1_ = 1.742, *p* = 0.356	1
Collective efficacy	−0.084	Underestimation (1 ES missing)	β_1_ = −5.067, *p* = 0.015^*^	2
Neighborhood cohesion	−0.065	Underestimation (2 ES missing)	β_1_ = −0.048, *p* = 0.987	1
Community involvement	−0.046	Underestimation (1 ES missing)	β_1_ = −0.335, *p* = 0.972	1
Neighborhood attachment	−0.034	-	β_1_ = −0.979, *p* = 0.567	0
Neighborhood safety	0.004	Overestimation (2 ES missing)	β_1_ = −2.340, *p* = 0.191	1
**School domains (#9)**				
School self-esteem	−0.209	-	β_1_ = −1.275, *p* = 0.490	0
Positive class or school climate	−0.180	Overestimation (3 ES missing)	β_1_ = −0.464, *p* = 0.736	1
School achievement	−0.176	-	β_1_ = −1.234, *p* = 0.092^+^	0
School involvement	−0.159	Underestimation (11 ES missing)	β_1_ = −0.023, *p* = 0.975	1
School attendance	−0.159	-	β_1_ = −3.458, *p* = 0.298	0
Positive attitude towards school	−0.156	Underestimation (3 ES missing)	β_1_ = 3.505, *p* = 0.008^**^	2
Student-teacher relationship	−0.152	Overestimation (9 ES missing)	β_1_ = −1.312, *p* = 0.277	1
Educational aspirations	−0.113	Overestimation (1 ES missing)	β_1_ = 62.146, *p* = <0.001^***^	2
Classmate support	−0.037	Overestimation (3 ES missing)	β_1_ = −2.508, *p* = 0.419	1

Dashes indicate that trimming and filling of effect sizes were not necessary according to the trim-and-fill algorithm

*r* Mean effect size (Pearson’s correlation; see also Table 2), *Overestimation* Effect sizes were imputed to the right of the mean effect, implying that small negative (or even slightly positive) effect sizes were underrepresented and that the mean effect may be an overestimation of the true effect, *Underestimation* Effect sizes were imputed to the left of the mean effect, implying that below large negative effect sizes were underrepresented and that the mean effect may be an underestimation of the true effect, *NA* Not available, as only two effect sizes (originated from one study) were classified in the corresponding protective domain

^+^*p* < 0.10; *p < 0.05; **p < 0.01; ***p < 0.001

^a^Except otherwise indicated, the labels of the protective domains have a positive direction (i.e., “Higher levels of…”)

### Moderator Analyses

Table 2 shows the results of the likelihood-ratio tests that were performed to examine heterogeneity in effect sizes within each protective domain. Significant within-study (level 2) and/or between-study (level 3) variance was found in 53 domains. In these domains, moderator analyses could be performed to examine the potential moderating effect of age, gender, and severity of antisocial behavior on the impact of individual protective domains. However, as these variables were only tested as a moderator when they were based on at least five studies (see the Method section), moderator analyses were performed for 49 protective domains.

Table 4 presents the results for the (significant) moderators. A significant moderating effect of age was found in five protective domains: The impact of the domains “empathy”, “parental control”, and “family cohesion” decreased as the age of the participants in samples increased, whereas the impact of the domain “emotional intelligence” and “quality of relationship with peers” increased as the age of the participants increased. For the percentage of males in primary study samples, a significant moderating effect was found in four protective domains: The impact of the domains “concentration skills”, “family cohesion”, and “peer support” decreased as the percentage of males in samples increased, whereas the impact of the “classmate support” domain increased as the percentage of males increased. Finally, for the severity of antisocial behavior, a significant moderating effect was found in the “coping skills” and “engagement in positive leisure activities” domains. A larger impact of these domains was found on severe antisocial behavior compared to less severe antisocial behavior (*r* = −0.113 versus *r* = −0.187 for “coping skills”; *r* = −0.058 versus *r* = −0.117 for “engagement in positive leisure activities”). For brevity, insignificant results are not reported in Table 4 and available upon request from the first author.

**Table 4:** Results of Significant Moderator Analyses

Moderator variables	# Studies	# ES	Intercept (95% CI)/ Mean *z* (95% CI)	β_1_ (95% CI)	*F* (df1, df2)^a^	*p*^b^	Level 2 variance	Level 3 variance
***Individual domains***								
Concentration skills								
Percentage males	5	15	−0.183 (−0.275, −0.091)^***^	0.952 (0.269, 1.636)^*^	9.061 (1, 13)	0.010^*^	0.024^***^	0.000
Emotional intelligence								
Age	6	22	−0.113 (−0.241, 0.015)^+^	−0.038 (−0.074, −0.003)^*^	5.102 (1, 20)	0.035^*^	0.001^+^	0.012^*^
Empathy								
Age	9	25	−0.164 (−0.234, −0.094)^***^	0.022 (0.006, 0.038)^*^	7.731 (1, 23)	0.011^*^	0.026^***^	0.000
Coping skills								
Severity of antisocial behavior					7.838 (1, 57)	0.007^**^	0.000	0.011
Less severe (RC)	12	38	−0.113 (−0.175, −0.050)^***^					
More severe	7	21	−0.187 (−0.256, −0.117)^***^	−0.074 (−0.127, −0.021)^**^				
Engagement in positive leisure activities	23	68			9.078 (1,66)	0.004^**^	0.001^*^	0.018^***^
Severity of antisocial behavior								
Less severe (RC)	11	24	−0.058 (−0.122, 0.006)^+^					
More severe	18	44	−0.117 (−0.178, −0.057)^***^	−0.059 (−0.098, −0.020)^**^				
***Family domains***								
Parental control								
Age	36	94	−0.209 (−0.255, −0.163)^***^	0.029 (0.013, 0.045)^***^	13.158 (1, 92)	<0.001^***^	0.003^***^	0.016^***^
Family cohesion								
Percentage males	42	104	−0.179 (−0.214, −0.143)^***^	0.105 (0.007, 0.204)^*^	4.513 (1, 102)	0.036^*^	0.010^***^	0.006^**^
Age	43	105	−0.194 (−0.229, −0.159)^***^	0.021 (0.009, 0.032)^***^	12.851 (1, 103)	<0.001^***^	0.008^***^	0.006^***^
***Non-family domains***								
Quality of relationship with peers								
Age	5	16	−0.162 (−0.301, −0.023)^*^	−0.588 (−1.147, −0.029)^*^	5.097 (1, 14)	0.040^*^	0.000	0.011^*^
Peer support								
Percentage males	17	30	−0.065 (−0.126, −0.004)^*^	0.522 (0.191, 0.853)^**^	10.409 (1, 28)	0.003^**^	0.003^***^	0.011^**^
***School domains***								
Classmate support								
Percentage males	6	13	−0.034 (−0.183, 0.115)	−0.091 (−0.161, −0.021)^*^	8.266 (1, 11)	0.015^*^	0.000	0.026^***^

*# Studies* number of studies, *# ES* number of effect sizes, *Mea*n *z* mean effect size (z), *CI* confidence interval, *β*_*1*_ estimated regression coefficient, *Level 2 variance* variance between effect sizes from the same study, *Level 3 variance* variance between studies; *RC* reference category

*p < 0.05; **p < 0.01; ***p < 0.001

^a^Omnibus test of all regression coefficients in the model

^b^*p* value of the omnibus test

## Discussion

A great amount of literature has reported on factors that are or may be protective against antisocial behavior in young people, but a comprehensive overview of factors that are truly protective against antisocial behavior was not yet available. This review provides such an overview by summarizing the results of primary studies on the impact of (potentially) protective factors on antisocial behavior of youth. Another aim was to examine whether and how age, gender, and type of antisocial behavior affect the impact of protective factors. In pursuing these aims, individual factors were first classified into one of 77 protective domains after which an overall effect for each of these domains was estimated and the potential moderating effects of age, percentage of males, and type of antisocial behavior on each overall effect were examined.

### Overall Impact of Protective Domains

Overall, a significant negative—and thus protecting—effect was found of 50 protective domains on antisocial behavior, with the largest impact for higher levels of conservativeness and the smallest impact for higher levels of family structure. Further, bias assessment strategy showed no indications of bias in the estimated impact of 18 protective domains, whereas the results showed some or strong indications of bias in the estimated impact of 59 domains. This bias may not be ascribed to publication bias alone. The trim and fill analyses showed that the estimated mean effects of 34 protective domains may be underestimations of the true effects, and 22 estimated mean effects may be overestimations of the true effects (see Table 3). These results imply that the mean effect of a substantial number of protective domains might have been affected by bias, which calls for a cautious interpretation of these domain effects.

The results revealed that multiple individual-, family-, extrafamilial-, community- and school-related factors are protective against antisocial behavior, which is in line with Bronfenbrenner’s ecological model (1979, 1986) in which he conceptualized that child development and behavior is affected by risk and protective factors in different social ecological systems surrounding the child. Moreover, protective domains related to individual characteristics (e.g., higher levels of conservativeness, self-transcendence, life satisfaction, the capacity to reflect or mentalize, and prosocial values) had significant and large or medium effects on antisocial behavior. This is in line with the assumption of Bronfenbrenner that factors in systems more proximal to the child exert more influence on development and behavior than factors in more distal systems. The current results revealed that the somewhat more distal factors show less impact than most proximal factors, but the impact is still substantial. For example, various extrafamilial- and school-related domains were found with a medium impact: having a romantic relationship, a better quality of peer relationships, having prosocial peers, and a higher level of school self-esteem.

In light of the guidelines of Funder and Ozer (2019) for interpreting the magnitude of effect sizes, a significant and large positive effect was identified for higher levels of conservativeness—meaning that an individual values tradition, conformity, and security—and higher levels of self-transcendence—meaning that an individual values universalism, benevolence, and the welfare of others. Conservativeness and self-transcendence are both personal values, which presumably underlie attitudes and behavior and are both central to an individual’s self-concepts (Vecchione et al., 2016). In line with the current findings, previous research suggests that being self-transcendent and conservative both act as protective factors for violent behavior (Knafo et al., 2008), bullying (Knafo, 2003; Menesini et al., 2013), aggression (Benish-Weisman & McDonald, 2015; Benish-Weisman, 2015), and antisocial behavior (Aquilar et al., 2018; Bacchini et al., 2015). Whether personal values are changeable is debated in the literature, and is an important question for clinical practice (Barnao, 2022). A key assumption shared by multiple theorists is that personal values exhibit substantial stability across situations and over time (e.g., Hitlin & Piliavin, 2004; Inglehart, 1977; Rokeach, 1973; Schwartz, 1992). Results of a recent review revealed moderate to high rank-order stabilities of personal values (Schuster et al., 2019) and thus underline this viewpoint. However, findings from this review also suggest that personal values can be changed in interventions involving cognitive justification of value importance, which is promising given the relatively strong negative associations found between the personal values conservativeness and self-transcendence, and antisocial behavior.

For 27 domains no significant effect was found, implying that insufficient evidence was obtained for designating these 27 domains as true protective domains. Many of these domains were related to ecological systems more distal to the individual, including characteristics of the community, school, and extra-familial relationships. This seems partly in line with the ecological model of Bronfenbrenner (1979, 1986) in the sense that the impact of factors decrease as factors are more distal to the child. Contrary to Bronfenbrenner’s reasoning no significant mean impact of these distal domains was found, but this may be explained by statistical power issues. Similarly, there were some individual- and family-related domains of which the effect was not significant, which may be due to insufficient statistical power.

In comparing the current findings with previous review studies on risk factors for antisocial behavior, similarities were in the magnitude of the overall effects. More specifically, a meta-analytic review on risk factors for persistent delinquent behavior (Assink et al., 2015) also revealed risk domains with small to large magnitudes. Furthermore, findings from this study showed that more distal risk factors (i.e., factors related to the neighborhood) yielded smaller effects compared to more proximal factors. In comparing the current results to another meta-analysis on risk factors for juvenile recidivism (Cottle et al., 2001), similarities in the impact of the risk and protective end of bipolar factors were found (e.g., delinquent peers vs. prosocial peers). Although these studies examined risk factors for criminal behavior rather than antisocial behavior, the similarities in the magnitude of impact may imply that risk and protective factors play an equal role in the etiology of antisocial behavior. This was also suggested by another study (Van der Put et al., 2011a), in which no significant difference was found between the impact of the protective and risk end of many bipolar factors (factors with a risk or a protective impact reflecting the ends of the same continuum) on criminal recidivism during adolescence.

### Moderating Effect of Age, Gender, and Type of Antisocial Behavior

An interesting question is whether and how the impact of protective domains is influenced by age and gender of young people showing antisocial behavior, and by the type of antisocial behavior they exhibit. As for age, it was found that higher levels of parental control and family cohesion are stronger protective factors for antisocial behavior in younger than in older individuals. In contrast, support from peers is a more protective factor for older than for younger individuals. These findings are in line with previous research on the effect of risk factors for delinquency and recidivism, revealing that the relative importance of family factors decreases as adolescents grow older, whereas the relative importance of school and friends increases (Loeber et al., 2008; Van der Put et al., 2011b; 2012). The current findings suggest that this may also be the case for certain protective factors.

As for the influence of gender on domain impact, differences were found in impact between males and females for several protective domains. For example, having good concentration skills and a high level of family cohesion seem to be stronger protective factors for antisocial behavior in females than in males. In a study on sex differences in risk factors for reoffending, it was found that girl-specific risk factors can particularly be identified within the family and that these factors are the most important predictors for reoffending (Van der Put et al., 2014). This echoes the current findings that stress the importance of family cohesion in protecting females against antisocial behaviors. Finally, a high level of classmate support was found to be a stronger protective factor for antisocial behavior in males than in females. This is in line with previous research on adolescents’ susceptibility to peer influence on delinquency, revealing that the effect of classmates’ delinquency was stronger for males (Müller et al., 2017).

Finally, it was found that engaging in active or problem-focused coping skills and engaging in positive leisure activities had stronger protective effects on more severe types of antisocial behavior (e.g., delinquency, assault, violence) compared to less severe types (e.g., behavioral problems, externalizing behavior, rule-breaking behavior). In general, active coping is consistently related to more adaptive outcomes, whereas avoidant coping is consistently related to more maladaptive outcomes (Bartek et al., 1993; Clarke, 2006; Compas et al., 2001; So et al., 2018). However, different studies have come to varied conclusions about how coping strategies affect aggressive or delinquent behaviors in youth. In some studies, avoidant coping is protective and associated with lower levels of behavioral arousal or delinquency in youth (Dempsey et al., 2000; Rosario et al., 2003), whereas other researchers found that avoidant coping is a vulnerability factor that leads to more aggression (Rosario et al., 2003; Scarpa & Haden, 2006).

In line with current findings for engaging in positive leisure activities, which often takes place at school or in the community, previous research suggest that school and neighborhood influences are stronger for more serious patterns of antisocial behavior (Conell et al., 2011). They suggest that this is consistent with theory and research proposing that socialization influences outside the family have a stronger association with more intense patterns of antisocial behavior involvement during adolescence (Ayers et al., 1999; Catalano & Hawkins, 1996).

It should be noted that for most protective domains no significant moderating effect was found for age, gender, nor the type of antisocial behavior, suggesting that most protective factors seem to have similar effects on antisocial behaviors across age, gender, and antisocial behaviors. This is not in line with previous research that found differences across age, gender, and behavioral sub-types in the impact of factors explaining antisocial behavior (e.g., Liu & Miller, 2020; Loeber, 1990; Scott & Brown, 2018; Van der Put et al., 2011a, 2011b). However, for many protective domains no moderator analyses were preformed, as variables were only tested as a moderator when they were based on at least five studies (per category of a variable).

### Limitations

This study has a number of important limitations, which were also identified in previous meta-analytic reviews on risk factors for various outcomes (e.g., Assink et al., 2019; Gubbels et al., 2019). First, despite an extensive search procedure, it cannot be assured that the sample of included studies is representative of all studies on (putative) protective factors for antisocial behavior. A large amount of literature is available on the effect of protective factors for antisocial behavior or delinquency, and therefore it is possible that primary studies were missed. However, given the current extensive data set (a total of 305 studies and 1,850 effect sizes), it may be assumed that the included studies were representative of all primary studies available on protective factors for antisocial behavior of juveniles and emerging adults. Further, the study inclusion was restricted to published studies and dissertations, and therefore there was a risk for overestimating domain effects due to publication bias. The bias assessment strategy indeed indicated that bias may have been present in the estimated effects of multiple domains. However, trim-and-fill analyses showed that for most domains an underestimation rather than an overestimation of the impact might have been the problem (see Table 3). Therefore, publication bias does not seem to be a substantial problem in the current review. An important reason for this may be that many included effect sizes were not central to the research questions that were addressed in primary studies, which reduced the risk of publication bias in the current review.

Second, conclusions about causality between protective factors and antisocial behavior cannot be drawn, because of the nonexperimental nature of the included studies. In addition, in extracting effects of (putative) protective factors from primary studies, there was a focus on antecedents of antisocial behavior, but as many included studies were retrospective in nature, it cannot be assured that all factors classified into the protective domains were true antecedents rather than outcomes. Further, the aim was to estimate mean effects of individual domains of protective factors, but it can be expected that an accumulation of factors is particularly predictive of antisocial behavior (see also Loeber et al., 1993; Stouthamer-Loeber et al., 2002). Another limitation is that current results do not allow for drawing strong conclusions about the relative importance of individual protective domains, as domains can be intercorrelated and may be mediated or moderated by other (protective or risk) domains. Bronfenbrenner (1979; 1986), for example, suggested that combinations of and interactions between protective domains across levels of a socio-ecological system may be particularly predictive of antisocial behavior. Examining interactions between multiple protective (and risk) factors is therefore an important challenge for future research.

Third, only age, percentage of males in samples of primary studies, and the type of antisocial behavior were examined as potential moderators of protective domain effects. After all, performing a large number of moderator analyses is not only impractical, but also statistically unwise, as insufficient data and capitalization on chance pose important problems. Further, it was decided to only perform moderator analyses for variables that could be coded for at least five studies. Most coded variables did not meet this criterion, as quite some protective domains consisted of a small number of studies and effect sizes. This study was therefore unable to elaborate on potential differences in domain impact across study designs or outcome characteristics. Relatedly, the influence of study quality indicators on domain impact were not tested because of insufficient data and/or a lack of variability within domains. The inclusion criteria did state that only primary studies that were subjected to some form of quality control were eligible for inclusion, but this could not prevent that studies of low quality were included. As a form of quality assessment, this review applied two techniques for assessing the risk of bias. The results revealed that some or substantial bias might be present in most domain effects although it should be taken into account that the performance of any bias assessment technique can be questioned when heterogeneous effect sizes are synthesized in three-level meta-analysis (e.g., Assink et al., 2019). The bias assessment results impede drawing firm conclusions on the true impact of the domains that were examined, and may particularly reveal that more rigorous primary research with samples of sufficient size are needed to better capture the true impact of protective factors for antisocial behavior in youth. After all, most of the examined protective domains were based on a rather small number of effect sizes and/or studies, and thus there is a risk for error and bias in the estimated impact of these domains. Therefore, the current results call for future high quality studies on protective factors for antisocial behavior in youth. A final note on the bias assessment is that effect sizes that were designated as “missing” in the trim-and-fill analysis might have been categorized in another domain, as there might be some overlap in content across domains (e.g., the “resilience” and “coping skills” domains) even though the aim was to classify protective factors of similar nature into mutually exclusive domains.

### Implications of the Study

The results have several implications. First, the findings contribute to the fundamental knowledge of the etiology (i.e., the study of causation or origination) of antisocial behavior, as they give insight into underlying factors associated with antisocial behavior in youth. Based on earlier research, it was already known that antisocial behavior are caused by multiple child, parent, family, peer, and school-related risk and protective factors (Howell, 2003; Loeber et al.,^1998^; ^2008^; Stouthamer-Loeber et al., 2002). This study adds knowledge about what variables can be identified as true protective factors (i.e., factors with a significant and negative effect on antisocial behavior), and which factors decrease the probability of antisocial behavior in juveniles and emerging adults the most.

The protective factors with a significant effect on antisocial behavior as identified in this review may contribute to the predictive value of assessment instruments. Adequate assessment strategies are essential in determining which individuals may develop antisocial behavior and would thus benefit from a (preventive) intervention. Current results indicate that many protective factors could reduce the probability of antisocial behavior in youth. Therefore, adding protective factors to risk assessment instrument, which now overwhelmingly focus on risk factors, could be considered. In literature it was noted that, theoretically, assessments that measure protective factors as well as risk factors could reduce false-positive bias because protective factors may buffer against the overvaluing of risk factors (DeMatteo et al., 2005). Such assessments would encourage clinicians to consider violent offenders as individuals with positive features rather than considering them as a mere sum of risk factors. This may strengthen the therapeutic relationship and improve assessment accuracy (Barnao et al., 2016; de Vries Robbé et al., 2013). Furthermore, currently available strengths-based instruments show good reliability and predictive validity. For instance, the Protective Factors for Reducing Juvenile Reoffending instrument (PFRJR; Barnes-Lee, 2020), which is a strengths-based measure designed to help juvenile justice practitioners understand the role of protective factors in potentially predicting and reducing juvenile recidivism, significantly predicts recidivism (Barnes-Lee & Campbell, 2020).

Further, as the results suggest that various factors can protect against antisocial behavior in youth, interventions for reducing or preventing antisocial behavior may benefit from strengthening protective factors. This strength-based approach is central to the Good Lives Model (GLM; Ward, 2010; Ward & Gannon, 2006; Ward et al., 2007), which is a model for offender rehabilitation in which an individual is hypothesized to commit criminal offences, as the capabilities to realize valued outcomes in personally fulfilling and socially acceptable ways are lacking. GLM prescribes that the focus should be on building people’s capabilities and strengths to reduce their risk of reoffending. Relative to a risk approach, applying this strengths-based approach should lead to fairer evaluations and more personalized treatment plans, and provide more motivating perspectives (Klepfisz et al., 2017; Ward & Fortune, 2014). Results from a systematic review suggest that GLM-consistent interventions are at least as effective as standard relapse prevention programs, but they enhance participants’ motivation to change and engagement in treatment (Mallion et al., 2020).

Notwithstanding the results of this review, the question remains how protective factors exert their influence on antisocial behavior across risk groups in which different numbers and combinations of risk and protective factors are present. In general, current results confirm the existence of a broad range of promotive factors in different ecological systems and imply that these factors can help reduce the probability of antisocial behavior. Consequently, the results suggest that promotive factors deserve attention in risk assessment strategies and (preventive) interventions for antisocial behavior. However, caution should be taken in drawing definitive inferences on the role of promotive factors. On the one hand, there is a body of research stating that “bad is stronger than good” (Baumeister et al., 2001) implying that risk factors exert more influence than protective factors. On the other, there is empirical support for a moderating effect of protective factors on the association between risk factors and antisocial behavior (e.g., Stoddard et al., 2012; 2013). A comprehensive understanding of the relative importance of risk and protective factors requires more future primary research in which accumulations of different risk and protective factors as well as their interactions are tested across groups with different risk levels. Such research can help improve the understanding of what risk factors, promotive factors, or a combination of both should be addressed under what circumstances in clinical practice. Also, assessing protective factors in risk assessment instruments seems promising, but thorough longitudinal validation of these instruments is required to validate the predictive value of protective factors over and above the predictive value of risk factors.

The results of this review do confirm that a broad and multifactorial perspective is needed in intervention and assessment strategies, as various individual, family, extrafamilial, and school factors were identified as being protective. Further, to effectively prevent or reduce antisocial behavior, it seems wise that clinical practitioners have an eye for the differences in impact of domains. Relative strong effects for protective domains related to characteristics of the individual were found (i.e., higher levels of conservativeness, self-transcendence, life satisfaction, the capacity to reflect or mentalize, prosocial values, agreeableness, general resilience, and social skills), suggesting that these factors deserve a relatively strong focus in interventions.

Finally, previous research noted that developmental and life-course theories of offending generally focus on main effects of risk factors and tend to ignore protective factors (Farrington et al., 2016). The current findings strengthen the idea that such theories can profit from incorporating protective factors, as over 40 individual, family, extrafamilial, community, and school domains were found with a significant protective effect on antisocial behavior.

## Conclusion

Both risk and protective factors are essential elements in etiological theories for antisocial behavior in youth, but compared to risk factors far less is known about protective factors and their impact. This meta-analytic review summarized results of primary research, so that insight is gained into factors that can be regarded as true protective factors for antisocial behavior in youth and into the strength of their impact. The results reveal that out of 77 examined factor domains (i.e., groups of protective factors that are more or less similar in nature), 50 domains can be designated as being truly protective, as their impact on antisocial behavior seemed to be significant and negative in direction. The most substantial effects were found for protective domains that refer to characteristics of the individual (e.g., high levels of conservativeness and high levels of self-transcendence), peers (e.g., better quality of peer relationships and having prosocial peers), and the school environment (e.g., positive class or school climate). Further, the results of the moderator analyses showed that the impact of only some factor domains is influenced by age, gender, or severity of antisocial behavior. The results suggest that a broad range of promotive factors should be considered in etiology of antisocial behavior, risk assessment strategies, and (preventive) interventions for antisocial behavior although future research is warranted on how risk and protective factors interact across youth with different risk levels for antisocial behavior.

### Supplementary Information

Supporting Information
