# Supplementary material for: Protective Factors for Antisocial Behavior in Youth: What is the Meta-Analytic Evidence?
Source: J Youth Adolesc. 2023 Oct 4;53(2):233–57. doi: 10.1007/s10964-023-01878-4 (PMC10764587; doi:10.1007/s10964-023-01878-4)
Supplement: Supplementary file 1 — Supporting Information [file 10964_2023_1878_MOESM1_ESM.docx]

**Protective Factors for Antisocial Behavior in Youth: What is the Meta-Analytic Evidence?**

*Journal of Youth and Adolescence*

Jeanne Gubbels^a^, Mark Assink^a^, Claudia E. van der Put^a^,

University of Amsterdam

^a^ Research Institute of Child Development and Education, University of Amsterdam, Nieuwe Achtergracht 127, 1018 WS, Amsterdam, the Netherlands.

Correspondence concerning this article should be addressed to Jeanne Gubbels: [J.Gubbels@uva.nl](mailto:J.Gubbels@uva.nl)

**Content**

Appendix S1 Search Syntax p. S2

Appendix S2 References to Studies Included in this Meta-Analysis p. S6

Appendix S3 Characteristics of Included Studies p. S57

Appendix S1

Search Syntax

The searches below yielded the following results: PsycINFO (10.076 references), ERIC (688 references), and MEDLINE (5.602 references).

**PsycINFO (Ovid, 1806 to April 20^th^, 2023)**

1. Aggressive Behavior/ or Aggressiveness/ or Antisocial Behavior/ or Arson/ or Child Abuse/ or Child Neglect/ or Crime/ or Criminal Behavior/ or Domestic Violence/ or Harassment/ or Homicide/ or Intimate Partner Violence/ or Kidnapping/ or Stalking/ or Recidivism/ or Shoplifting/ or Theft/ or Vandalism/ or Violence/ or Violent Crime/ or (aggressi* or antisocial behavio* or anti-social behavio* or arson* or assault or burglary or child abuse or child neglect or criminal behav* or drug* crim* or drug offense or drug offending or drunk driving or firesetting or (fire adj2 setting) or harassment or homicide* or larceny or partner abus* or manslaughter* or murder* or property* crim* or property offen* or recidiv* or rearrest* or reoffen* or re-offen* or reincarcerat* or re-incarcerat* or reconvict* or re-convict* or robbery or shoplift* or stalk* or theft* or traffic offen* or vandalism or violen*).ti,ab,id. or (aggress* or antisocial or conduct disorder* or ((defiant or disruptive or dysfunctional* or maladaptiv* or problem*) adj3 (behavio* or disorder*)) or externali* or misbehavio* or misconduct).ti,ab,id.
2. Protective Factors/ or "Resilience (Psychological)"/ or (protective factor* or protective effect* or promotive factor* or promotive effect* or resilien* factor* or resilien* effect* or buffer* factor* or buffer* effect* or compensatory factor* or compensatory effect* or resource factor* or resource effect* or beneficial factor* or beneficial effect* or positive outcome* or risk assess*).ti,ab,id.
3. Quantitative Methods/ or (followup study or longitudinal study or meta*analys*s or quantitative study).md. or (SEM or structural equation* model* or CFA or confirmatory factor analys*s or path model* or path analys*s or factor model* or factor analys*s or cross-sectional or longitudinal or quantitat* or ancova or anova or cohen*s D or control condition* or control group* or effect size* or inventor* or regression* or measurement* or mediator* or moderator* or odds ratio or questionnair* or scale* or instrument* or tool* or correlat*).ti,ab,id,tm.
4. 1 AND 2 AND 3

Key: / = subject heading, ti = title, ab = abstract, id = key concepts (other keywords added by PsycINFO indexers to supplement the subject headings), tm = tests & measures.

**ERIC (Ovid, 1965 to April 2023)**

1. Aggression/ or Antisocial Behavior/ or Child Abuse/ or Child Neglect/ or Crime/ or Family Violence/ or Homicide/ or Recidivism/ or Vandalism/ or Violence/ or (aggressi* or antisocial behavio* or anti-social behavio* or arson* or assault or burglary or child abuse or child neglect or criminal behav* or drug* crim* or drug offense or drug offending or drunk driving or firesetting or (fire adj2 setting) or harassment or homicide* or larceny or partner abus* or manslaughter* or murder* or property* crim* or property offen* or recidiv* or rearrest* or reoffen* or re-offen* or reincarcerat* or re-incarcerat* or reconvict* or re-convict* or robbery or shoplift* or stalk* or theft* or traffic offen* or vandalism or violen*).ti,ab,id. or (aggress* or antisocial or conduct disorder* or ((defiant or disruptive or dysfunctional* or maladaptiv* or problem*) adj3 (behavio* or disorder*)) or externali* or misbehavio* or misconduct).ti,ab,id.
2. Protective Factors/ or "Resilience (Psychological)"/ or (protective factor* or protective effect* or promotive factor* or promotive effect* or resilien* factor* or resilien* effect* or buffer* factor* or buffer* effect* or compensatory factor* or compensatory effect* or resource factor* or resource effect* or beneficial factor* or beneficial effect* or positive outcome* or risk assess*).ti,ab,id.
3. Quantitative Methods/ or (followup study or longitudinal study or meta*analys*s or quantitative study) or (SEM or structural equation* model* or CFA or confirmatory factor analys*s or path model* or path analys*s or factor model* or factor analys*s or cross-sectional or longitudinal or quantitat* or ancova or anova or cohen*s D or control condition* or control group* or effect size* or inventor* or regression* or measurement* or mediator* or moderator* or odds ratio or questionnair* or scale* or instrument* or tool* or correlat*).ti,ab,id.
4. 1 AND 2 AND 3
5. Limit 4 to peer review

**MEDLINE (Ovid MEDLINE[R] and Epub Ahead of Print, In-Process & Other Non-Indexed Citations and Daily 1946 to April 20th, 2023)**

1. Aggressive Behavior/ or Aggressiveness/ or Antisocial Behavior/ or Arson/ or Child Abuse/ or Child Neglect/ or Crime/ or Criminal Behavior/ or Domestic Violence/ or Harassment/ or Homicide/ or Intimate Partner Violence/ or Kidnapping/ or Stalking/ or Recidivism/ or Shoplifting/ or Theft/ or Vandalism/ or Violence/ or Violent Crime/ or (aggressi* or antisocial behavio* or anti-social behavio* or arson* or assault or burglary or child abuse or child neglect or criminal behav* or drug* crim* or drug offense or drug offending or drunk driving or firesetting or (fire adj2 setting) or harassment or homicide* or larceny or partner abus* or manslaughter* or murder* or property* crim* or property offen* or recidiv* or rearrest* or reoffen* or re-offen* or reincarcerat* or re-incarcerat* or reconvict* or re-convict* or robbery or shoplift* or stalk* or theft* or traffic offen* or vandalism or violen*).ti,ab,id. or (aggress* or antisocial or conduct disorder* or ((defiant or disruptive or dysfunctional* or maladaptiv* or problem*) adj3 (behavio* or disorder*)) or externali* or misbehavio* or misconduct).ti,ab,kf.
2. Protective Factors/ or "Resilience (Psychological)"/ or (protective factor* or protective effect* or promotive factor* or promotive effect* or resilien* factor* or resilien* effect* or buffer* factor* or buffer* effect* or compensatory factor* or compensatory effect* or resource factor* or resource effect* or beneficial factor* or beneficial effect* or positive outcome* or risk assess*).ti,ab,kf.
3. Quantitative Methods/ or (followup study or longitudinal study or meta*analys*s or quantitative study) or (SEM or structural equation* model* or CFA or confirmatory factor analys*s or path model* or path analys*s or factor model* or factor analys*s or cross-sectional or longitudinal or quantitat* or ancova or anova or cohen*s D or control condition* or control group* or effect size* or inventor* or regression* or measurement* or mediator* or moderator* or odds ratio or questionnair* or scale* or instrument* or tool* or correlat*).ti,ab,kf.
4. 1 AND 2 AND 3

Key: / = medical subject heading (MeSH), ti = title, ab = abstract, kf = author supplied keywords

Appendix S2

References to Studies Included in this Meta-Analysis

Aceves, M. J., & Cookston, J. T. (2007). Violent victimization, aggression, and parent-adolescent relations: Quality parenting as a buffer for violently victimized youth. *Journal of Youth and Adolescence*, *36*(5), 635–647. <https://doi.org/10.1007/s10964-006-9131-9>

Al-Yagon, M. (2015). Externalizing and Internalizing Behaviors Among Adolescents with Learning Disabilities: Contribution of Adolescents’ Attachment to Mothers and Negative Affect. *Journal of Child and Family Studies*, *24*(5), 1343–1357. <https://doi.org/10.1007/s10826-014-9942-3>

Allbaugh, L. J. (2018). *Female adolescent trauma survivors and their parents: Change in quality of bond as a predictor of later vulnerability or resilience*. [Doctoral dissertation, Miami University]. OhioLINK. <https://etd.ohiolink.edu/apexprod/rws_etd/send_file/send?accession=miami1499861051275347&disposition=inline>

Álvarez-García, D., González-Castro, P., Núñez, J. C., Rodríguez, C., & Cerezo, R. (2019). Impact of Family and Friends on Antisocial Adolescent Behavior: The Mediating Role of Impulsivity and Empathy. *Frontiers in Psychology*, *10*, 1–9. <https://doi.org/10.3389/fpsyg.2019.02071>

Anderson, C. A., Suzuki, K., Swing, E. L., Groves, C. L., Gentile, D. A., Prot, S., Lam, C. P., Sakamoto, A., Horiuchi, Y., Krahé, B., Jelic, M., Liuqing, W., Toma, R., Warburton, W. A., Zhang, X. M., Tajima, S., Qing, F., & Petrescu, P. (2017). Media Violence and Other Aggression Risk Factors in Seven Nations. *Personality and Social Psychology Bulletin*, *43*(7), 986–998. <https://doi.org/10.1177/0146167217703064>

Anderson, S., Donlan, A. E., McDermott, E. R., & Zaff, J. F. (2015). Ecology matters: Neighborhood differences in the protective role of self-control and social support for adolescent antisocial behavior. *American Journal of Orthopsychiatry*, *85*(6), 536–549. <https://doi.org/10.1037/ORT0000124>

Appleyard, K., Egeland, B., & Sroufe, L. A. (2007). Direct social support for young high risk children: Relations with behavioral and emotional outcomes across time. *Journal of Abnormal Child Psychology, 35*(3), 443–457. https://doi.org/10.1007/s10802-007-9102-y

Apter, A., Gothelf, D., Offer, R., Ratzoni, G., Orbach, I., Tyano, S., & Pfeffer, C. R. (1997). Suicidal adolescents and ego defense mechanisms. *Journal of the American Academy of Child and Adolescent Psychiatry*, *36*(11), 1520–1527. <https://doi.org/10.1016/s0890-8567(09)66560-6>

Aquilar, S., Bacchini, D., & Affuso, G. (2018). Three-year cross-lagged relationships among adolescents’ antisocial behavior, personal values, and judgment of wrongness. *Social Development*, *27*(2), 381–400. <https://doi.org/10.1111/sode.12279>

Aronowitz, T., & Morrison-Beedy, D. (2004). Resilience to risk-taking behaviors in impoverished African American girls: The role of mother-daughter connectedness. *Research in Nursing and Health*, *27*(1), 29–39. <https://doi.org/10.1002/nur.20004>

Aspy, C. B., Oman, R. F., Vesely, S. K., McLeroy, K., Rodine, S., & Marshall, L. (2004). Adolescent violence: The protective effects of youth assets*. Journal of Counseling & development, 82*(3), 268-276. <https://doi.org/10.1002/j.1556-6678.2004.tb00310.x>

Bacchini, D., Miranda, M. C., & Affuso, G. (2011). Effects of parental monitoring and exposure to community violence on antisocial behavior and anxiety/depression among adolescents. *Journal of Interpersonal Violence*, *26*(2), 269–292. <https://doi.org/10.1177/0886260510362879>

Bacon, A. M., Burak, H., & Rann, J. (2014). Sex differences in the relationship between sensation seeking, trait emotional intelligence and delinquent behaviour. *Journal of Forensic Psychiatry and Psychology*, *25*(6), 673–683. <https://doi.org/10.1080/14789949.2014.943796>

Baier, D., Krieg, Y., Hong, J. S., & Kliem, S. (2021). Who beats their partner, and who beats their schoolmates? A comparison of teen dating and school physical violence perpetration in Lower Saxony, Germany. *New directions for child and adolescent development, 178*, 79–93. <https://doi.org/10.1002/cad.20439>

Bakker, M. P., Ormel, J., Verhulst, F. C., & Oldehinkel, A. J. (2011). Adolescent family adversity and mental health problems: The role of adaptive self-regulation capacities. The TRAILS study. *Journal of Abnormal Child Psychology*, *39*(3), 341–350. <https://doi.org/10.1007/s10802-010-9470-6>

Banerjee, M. (2012). *Testing the moderating effects of racial socialization on the relationship between community violence exposure and academic/psychosocial outcomes in African American young adults* (Publication No. 3525940) [Doctoral dissertation, Michigan State University ]. ProQuest Dissertations and Theses Global.

Barger, J., Vitale, P., Gaughan, J. P., & Feldman-Winter, L. (2017). Measuring Resilience in the Adolescent Population: A Succinct Tool for Outpatient Adolescent Health. *Journal of Pediatrics*, *189*, 201-206.e3. <https://doi.org/10.1016/j.jpeds.2017.06.030>

Barnes, G. M., Hoffman, J. H., Welte, J. W., Farrell, M. P., & Dintcheff, B. A. (2007). Adolescents’ time use: Effects on substance use, delinquency and sexual activity. *Journal of Youth and Adolescence*, *36*(5), 697–710. <https://doi.org/10.1007/s10964-006-9075-0>

Barnes-Lee, A. R., & Campbell, C. A. (2020). Protective factors for reducing juvenile reoffending: an examination of incremental and differential predictive validity. *Criminal Justice and Behavior, 47*(11), 1390-1408. <https://doi.org/10.1177/0093854820952115>

Barry, C. T., Loflin, D. C., & Doucette, H. (2015). Adolescent self-compassion: Associations with narcissism, self-esteem, aggression, and internalizing symptoms in at-risk males. *Personality and Individual Differences*, *77*, 118–123. <https://doi.org/10.1016/j.paid.2014.12.036>

Barry, C. T., Lui, J. H. L., & Anderson, A. C. (2017). Adolescent Narcissism, Aggression, and Prosocial Behavior: The Relevance of Socially Desirable Responding. *Journal of Personality Assessment*, *99*(1), 46–55. <https://doi.org/10.1080/00223891.2016.1193812>

Bates, E. A., Archer, J., & Graham-Kevan, N. (2017). Do the same risk and protective factors influence aggression toward partners and same-sex others? *Aggressive Behavior*, *43*(2), 163–175. <https://doi.org/10.1002/ab.21672>

Beam, M. R., Gil-Rivas, V., Greenberger, E., & Chen, C. (2002). Adolescent problem behavior and depressed mood: Risk and protection within and across social contexts. *Journal of Youth and Adolescence*, *31*(5), 343–357. <https://doi.org/10.1023/A:1015676524482>

Beaver, K. M., Nedelec, J. L., Wilde, M., Lippoff, C., & Jackson, D. (2011). Examining the association between MAOA genotype and incarceration, anger and hostility: The moderating influences of risk and protective factors. *Journal of Research in Personality*, *45*(3), 279–284. <https://doi.org/10.1016/j.jrp.2011.02.007>

Beckmann, L. (2021). Does parental warmth buffer the relationship between parent-to-child physical and verbal aggression and adolescent behavioural and emotional adjustment? *Journal of Family Studies, 27*(3), 366-387. <https://doi.org/10.1080/13229400.2019.1616602>

Beckmann, L., Bergmann, M. C., Fischer, F., & Mößle, T. (2017). Risk and protective factors of child-to-parent violence: A comparison between physical and verbal aggression. *Journal of Interpersonal Violence, 36*(3-4), NP1309-1334NP. <https://doi.org/10.1177/0886260517746129>

Bernat, D. H., Oakes, J. M., Pettingell, S. L., & Resnick, M. (2012). Risk and direct protective factors for youth violence: Results from the national longitudinal study of adolescent health. *American Journal of Preventive Medicine*, *43*, S57–S66. <https://doi.org/10.1016/j.amepre.2012.04.023>

Blazei, R. W., Iacono, W. G., & McGue, M. (2008). Father-child transmission of antisocial behavior: The moderating role of father’s presence in the home. *Journal of the American Academy of Child and Adolescent Psychiatry*, *47*(4), 406–415. <https://doi.org/10.1097/CHI.0b013e3181642979>

Blum, J., Ireland, M., & Blum, R. W. (2003). Gender differences in juvenile violence: A report from Add Health. *Journal of Adolescent Health*, *32*(3), 234–240. <https://doi.org/10.1016/S1054-139X(02)00448-2>

Boccio, C. I. M. (2018). *Examining Positive Life Outcomes in the Face of Antisocial Traits*. (Publication No. 10817501) [Doctoral dissertation, The Florida State University]. ProQuest Dissertations and Theses Global.

Bondü, R., & Elsner, B. (2015). Justice sensitivity in childhood and adolescence. *Social Development*, *24*(2), 420–441. <https://doi.org/10.1111/sode.12098>

Botcheva, L. B., Shirley Feldman, S., & Herbert Leiderman, P. (2002). Can stability in school processes offset the negative effects of sociopolitical upheaval on adolescents’ adaptation? *Youth and Society*, *34*(1), 55–88. <https://doi.org/10.1177/0044118X02034001003>

Boyas, J. F., Kim, Y. J., Sharpe, T. L., Moore, D. J., & Prince-Stehley, K. (2017). An ecological path model of use of violence among African American adolescents. *Child and Youth Services*, *38*(1), 24–52. <https://doi.org/10.1080/0145935X.2016.1204537>

Brieant, A., Holmes, C. J., Maciejewski, D., Lee, J., Deater-Deckard, K., King-Casas, B., & Kim-Spoon, J. (2018). Positive and Negative Affect and Adolescent Adjustment: Moderation Effects of Prefrontal Functioning. *Journal of Research on Adolescence*, *28*(1), 40–55. <https://doi.org/10.1111/jora.12339>

Brody, G. H., Yu, T., Chen, E., Miller, G. E., Kogan, S. M., & Beach, S. R. H. (2013). Is Resilience Only Skin Deep? Rural African Americans’ Socioeconomic Status-Related Risk and Competence in Preadolescence and Psychological Adjustment and Allostatic Load at Age 19. *Psychological Science*, *24*(7), 1285–1293. <https://doi.org/10.1177/0956797612471954>

Brookmeyer, K. A., Fanti, K. A., & Henrich, C. C. (2006). Schools, parents, and youth violence: A multilevel, ecological analysis. *Journal of Clinical Child and Adolescent Psychology*, *35*(4), 504–514. <https://doi.org/10.1207/s15374424jccp3504_2>

Browning, S., & Erickson, P. (2012). Neighbourhood Variation in the Link between Alcohol Use and Violence among Canadian Adolescents  . *Canadian Journal of Criminology and Criminal Justice*, *54*(2), 169–201. <https://doi.org/10.3138/cjccj.2009.e.37>

Bruett, L. D. (2015). *Longitudinal predictors of subjective wellbeing and adaptive functioning in early adulthood: Internalizing and externalizing behaviors, emotion regulation, parenting, and substance use* [Doctoral dissertation, Temple University ]. <https://scholarshare.temple.edu/handle/20.500.12613/872>

Buote, D. (2006). *The power of connection: The relation between attachment and resiliency in a sample of high risk adolescents* [Doctoral dissertation, The University of British Columbia]. <https://open.library.ubc.ca/media/download/pdf/831/1.0054364/1>

Burnside, E. J. (2015). *Features of the organized youth activity setting that protect against exposure to community violence*. [Doctoral dissertation, University of Illinois at Chicago]. <https://indigo.uic.edu/articles/thesis/Features_of_the_Organized_Youth_Activity_Setting_that_Protect_Against_Exposure_to_Community_Violence/10849721/1>

Burt, C. H., Simons, R. L., & Gibbons, F. X. (2012). Racial Discrimination, Ethnic-Racial Socialization, and Crime: A Micro-sociological Model of Risk and Resilience. *American Sociological Review*, *77*(4), 648–677. <https://doi.org/10.1177/0003122412448648>

Caiozzo, C. N., Houston, J., & Grych, J. (2016). Predicting aggression in late adolescent romantic relationships: A short-term longitudinal study. *Journal of Adolescence*, *53*, 237–248. <https://doi.org/10.1016/j.adolescence.2016.10.012>

Caldwell, C. H., Kohn-Wood, L. P., Schmeelk-Cone, K. H., Chavous, T. M., & Zimmerman, M. A. (2004). Racial discrimination and racial identity as risk or protective factors for violent behaviors in African American young adults. *American Journal of Community Psychology*, *33*(1–2), 91–105. <https://doi.org/10.1023/B:AJCP.0000014321.02367.dd>

Caldwell, L. L., & Smith, E. A. (2013). Leisure as a context for youth development and delinquency prevention. *Pathways and Crime Prevention: Theory, Policy and Practice*, *39*(3), 271–297. <https://doi.org/10.4324/9781843926481>

Calvete, E., Orue, I., & Sampedro, A. (2017). Does the acting with awareness trait of mindfulness buffer the predictive association between stressors and psychological symptoms in adolescents? *Personality and Individual Differences*, *105*, 158–163. <https://doi.org/10.1016/j.paid.2016.09.055>

Calzada, E. J., Roche, K. M., White, R. M. B., Partovi, R., & Little, T. D. (2020). Family strengths and Latinx youth externalizing behavior: Modifying impacts of an adverse immigration environment. *Journal of Latinx Psychology*, *8*(4), 332–348. <https://doi.org/10.1037/lat0000162>

Campos, J., Barbosa-Ducharne, M., Dias, P., Rodrigues, S., Martins, A. C., & Leal, M. (2019). Emotional and Behavioral Problems and Psychosocial Skills in Adolescents in Residential Care. *Child and Adolescent Social Work Journal*, *36*(3), 237–246. <https://doi.org/10.1007/s10560-018-0594-9>

Carlson, M., Oshri, A., & Kwon, J. (2015). Child maltreatment and risk behaviors: The roles of callous/unemotional traits and conscientiousness. *Child Abuse and Neglect*, *50*, 234–243. <https://doi.org/10.1016/j.chiabu.2015.07.003>

Cavanaugh, A. M., Stein, G. L., Supple, A. J., Gonzalez, L. M., & Kiang, L. (2018). Protective and Promotive Effects of Latino Early Adolescents’ Cultural Assets Against Multiple Types of Discrimination. *Journal of Research on Adolescence*, *28*(2), 310–326. <https://doi.org/10.1111/jora.12331>

Champion, H., Foley, K. L., Sigmon-Smith, K., Sutfin, E. L., & DuRant, R. H. (2008). Contextual factors and health risk behaviors associated with date fighting among high school students. *Women & Health, 47*(3), 1-22. <https://doi.org/10.1080/03630240802132286>

Chapman, M. L. (2018). *Trauma and resilience among maltreated youth: The role of social support in promoting academic and social-emotional well-being* [Doctoral dissertation, Michigan State University]. <https://d.lib.msu.edu/etd/6686/datastream/OBJ/download/Trauma_and_Resilience_Among_Maltreated_Youth___The_Role_of_Social_Support_in_Promoting_Academic_and_Social-Emotional_Well-Being.pdf>

Chappel, A. M. (2013). *A longitudinal investigation of stress, complete mental health, and social support among high school students* [Doctoral dissertation, University of South Florida ]. <http://ovidsp.ovid.com/ovidweb.cgi?T=JS&PAGE=reference&D=psyc10&NEWS=N&AN=2013-99240-110>

Chen, P., Voisin, D. R., & Jacobson, K. C. (2016). Community Violence Exposure and Adolescent Delinquency: Examining a Spectrum of Promotive Factors. *Youth and Society*, *48*(1), 33–57. <https://doi.org/10.1177/0044118X13475827>

Chinchilla, M. A., & Kosson, D. S. (2016). Psychopathic Traits Moderate Relationships Between Parental Warmth and Adolescent Antisocial and Other High-Risk Behaviors. *Criminal Justice and Behavior*, *43*(6), 722–738. https://doi.org/10.1177/0093854815617216

Choi, Y., Karachi, T. W., Gillmore, M. R., & Catalano, R. F. (2005). Applicability of the social development model to urban ethnic minority youth: Examining the relationship between external constraints, family socialization, and problem behaviors. *Journal of Research on Adolescence*, *15*(4), 505–534. https://doi.org/10.1111/j.1532-7795.2005.00109.x

Chuang, S. S., Lamb, M. E., & Hwang, C. P. (2006). Personality development from childhood to adolescence: A longitudinal study of ego-control and ego-resiliency in Sweden. *International Journal of Behavioral Development*, *30*(4), 338–343. https://doi.org/10.1177/0165025406072795

Chung, H. L., & Docherty, M. (2011). The protective function of neighborhood social ties on psychological health. *American Journal of Health Behavior*, *35*(6), 785–796. https://doi.org/10.5993/AJHB.35.6.14

Copeland-Linder, N., Lambert, S. F., Chen, Y. F., & Ialongo, N. S. (2011). Contextual Stress and Health Risk Behaviors Among African American Adolescents. *Journal of Youth and Adolescence*, *40*(2), 158–173. https://doi.org/10.1007/s10964-010-9520-y

Copeland-Linder, N., Lambert, S. F., & Ialongo, N. S. (2010). Community violence, protective factors, and adolescent mental health: A profile analysis. *Journal of Clinical Child and Adolescent Psychology*, *39*(2), 176–186. https://doi.org/10.1080/15374410903532601

Coyne, S. M., Padilla-Walker, L. M., Day, R. D., Harper, J., & Stockdale, L. (2014). A friend request from dear old dad: Associations between parent-child social networking and adolescent outcomes. *Cyberpsychology, Behavior, and Social Networking*, *17*(1), 8–13. https://doi.org/10.1089/cyber.2012.0623

Craig, J. M., Baglivio, M. T., Wolff, K. T., Piquero, A. R., & Epps, N. (2017). Do Social Bonds Buffer the Impact of Adverse Childhood Experiences on Reoffending? *Youth Violence and Juvenile Justice*, *15*(1), 3–20. https://doi.org/10.1177/1541204016630033

Criss, M. M., Smith, A. M., Morris, A. S., Liu, C., & Hubbard, R. L. (2017). Parents and peers as protective factors among adolescents exposed to neighborhood risk. *Journal of Applied Developmental Psychology*, *53*(November), 127–138. https://doi.org/10.1016/j.appdev.2017.10.004

Cropp, C., Alexandrowicz, R. W., & Taubner, S. (2019). Reflective functioning in an adolescent community sample. *Mental Health and Prevention*, *14*(May). <https://doi.org/10.1016/j.mph.2019.200156>

Culyba, A. J., Ginsburg, K. R., Fein, J. A., Branas, C. C., Richmond, T. S., & Wiebe, D. J. (2016). Protective effects of adolescent-adult connection on male youth in urban environments. *Journal of Adolescent Health*, *58*(2), 237–240. https://doi.org/10.1016/j.jadohealth.2015.10.247

Cunningham, M., Francois, S., Rodriguez, G., & Lee, X. W. (2018). Resilience and Coping: An Example in African American Adolescents. *Research in Human Development*, *15*(3–4), 317–331. https://doi.org/10.1080/15427609.2018.1502547

Curran, P. J., & Chassin, L. (1996). A longitudinal study of parenting as a protective factor for children of alcoholics. *Journal of Studies on Alcohol*, *57*(3), 305–313. https://doi.org/10.15288/jsa.1996.57.305

Cutrín, O., Gómez-Fraguela, J. A., & Sobral, J. (2017). Two Faces of Parental Support: Risk and Protection for Antisocial Youth Depending on Parental Knowledge. *Journal of Child and Family Studies*, *26*(1), 296–305. https://doi.org/10.1007/s10826-016-0559-6

Davis, S. B. (2017). *Sense of belonging, emotion regulation, perceived social support and mental health among college students* [Doctoral dissertation, Norfolk State University]. <https://digitalcommons.odu.edu/cgi/viewcontent.cgi?article=1053&context=psychology_etds>

de la Barrera, U., Schoeps, K., Gil-Gómez, J. A., & Montoya-Castilla, I. (2019). Predicting adolescent adjustment and well-being: The interplay between socio-emotional and personal factors. *International Journal of Environmental Research and Public Health*, *16*(23). https://doi.org/10.3390/ijerph16234650

de la Rubia, J., & Morales, H. (2012). Risk and Protective Factors of Dissocial Behavior in a Probability Sample. *The Spanish Journal of Psychology, 15*(2), 736-747. <https://doi.org/10.5209/rev_SJOP.2012.v15.n2.38885>

de Vries, S. L. A., Hoeve, M., Stams, G. J. J. M., & Asscher, J. J. (2016). Adolescent-Parent Attachment and Externalizing Behavior: The Mediating Role of Individual and Social Factors. *Journal of Abnormal Child Psychology*, *44*(2), 283–294. https://doi.org/10.1007/s10802-015-9999-5

Deane, K., Richards, M., Mozley, M., Scott, D., Rice, C., & Garbarino, J. (2018). Posttraumatic Stress, Family Functioning, and Externalizing in Adolescents Exposed to Violence: A Moderated Mediation Model. *Journal of Clinical Child and Adolescent Psychology*, *47*(sup1), S176–S189. https://doi.org/10.1080/15374416.2016.1197836

DeGruy, J., Kjellstrand, J. M., Briggs, H. E., & Brennan, E. M. (2012). Racial Respect and Racial Socialization as Protective Factors for African American Male Youth. *Journal of Black Psychology*, *38*(4), 395–420. https://doi.org/10.1177/0095798411429744

DeSantis King, A. L., Huebner, S., Suldo, S. M., & Valois, R. F. (2006). An ecological view of school satisfaction in adolescence: Linkages between social support and behavior problems. *Applied Research in Quality of Life*, *1*(3–4), 279–295. <https://doi.org/10.1007/s11482-007-9021-7>

DuPont-Reyes, M., Fry, D., Rickert, V., & Davidson, L. L. (2015). Adolescent Relationship Violence and Acculturation Among NYC Latinos. *Maternal and Child Health Journal*, *19*(7), 1543–1552. <https://doi.org/10.1007/s10995-014-1659-9>

El-Sheikh, M., Shimizu, M., Philbrook, L. E., Erath, S. A., & Buckhalt, J. A. (2020). Sleep and development in adolescence in the context of socioeconomic disadvantage. *Journal of Sdolescence, 83*, 1-11. <https://doi.org/10.1016/j.adolescence.2020.06.006>

Ernst, J. L. (2000). *Genetic and environmental influences of maternal psychosocial and antisocial tendencies on the development, stability, and continuity of problem behaviors in adoptees from the Texas Adoption Project: A life course investigation of risk, resilience, and vulnerability* [Doctoral dissertation, University of Texas]. <https://repositories.lib.utexas.edu/handle/2152/3446>

Estévez López, E., Pérez, S. M., Ochoa, G. M., & Ruiz, D. M. (2008). Adolescent aggression: Effects of gender and family and school environments. *Journal of Adolescence*, *31*(4), 433–450. https://doi.org/10.1016/j.adolescence.2007.09.007

Farruggia, S. P., & Germo, G. R. (2015). Problem behavior among older youth in foster care: Examining gender. *Children and Youth Services Review*, *48*, 20–30. https://doi.org/10.1016/j.childyouth.2014.11.005

Fernández-González, L., Calvete, E., Orue, I., & Echezarraga, A. (2018). The role of emotional intelligence in the maintenance of adolescent dating violence perpetration. *Personality and Individual Differences*, *127*(September 2017), 68–73. https://doi.org/10.1016/j.paid.2018.01.038

Fitzpatrick, S., & Bussey, K. (2014). The role of perceived friendship self-efficacy as a protective factor against the negative effects of social victimization. *Social Development*, *23*(1), 41–60. https://doi.org/10.1111/sode.12032

Flouri, E., & Mavroveli, S. (2013). Adverse life events and emotional and behavioural problems in adolescence: The role of coping and emotion regulation. *Stress and Health*, *29*(5), 360–368. https://doi.org/10.1002/smi.2478

Flouri, E., Mavroveli, S., & Panourgia, C. (2013). The role of general cognitive ability in moderating the relation of adverse life events to emotional and behavioural problems. *British Journal of Psychology*, *104*(1), 130–139. https://doi.org/10.1111/j.2044-8295.2012.02106.x

Folger, S. F., & Wright, M. O. D. (2013). Altering Risk Following Child Maltreatment: Family and Friend Support as Protective Factors. *Journal of Family Violence*, *28*(4), 325–337. https://doi.org/10.1007/s10896-013-9510-4

Forster, M., Grigsby, T. J., Soto, D. W., Sussman, S. Y., & Unger, J. B. (2017). Perceived discrimination, cultural identity development, and intimate partner violence among a sample of hispanic young adults. *Cultural Diversity and Ethnic Minority Psychology*, *23*(4), 576–582. https://doi.org/10.1037/cdp0000154

Froidevaux, N. M., Metcalf, S., Pettit, C., Penner, F., Sharp, C., & Borelli, J. L. (2020). The Link Between Adversity and Dating Violence Among Adolescents Hospitalized for Psychiatric Treatment: Parental Emotion Validation as a Candidate Protective Factor. *Journal of Interpersonal Violence*. https://doi.org/10.1177/0886260520926323

Fung, J. J., & Lau, A. S. (2009). Punitive discipline and child behavior problems in Chinese-American immigrant families: The moderating effects of indigenous child-rearing ideologies. *International Journal of Behavioral Development*, *33*(6), 520–530. https://doi.org/10.1177/0165025409343749

Garaigordobil, M. (2015). Predictor variables of happiness and its connection with risk and protective factors for health. *Frontiers in Psychology*, *6*(August), 1–10. https://doi.org/10.3389/fpsyg.2015.01176

Gardner, T. W., Dishion, T. J., & Connell, A. M. (2008). Adolescent self-regulation as resilience: Resistance to antisocial behavior within the deviant peer context. *Journal of Abnormal Child Psychology*, *36*(2), 273–284. https://doi.org/10.1007/s10802-007-9176-6

Garrido, E. F., & Taussig, H. N. (2013). Do parenting practices and prosocial peers moderate the association between intimate partner violence exposure and teen dating violence? *Psychology of Violence*, *3*(4), 354–366. https://doi.org/10.1037/a0034036

Gartstein, M., Seamon, E., & Dishion, T. J. (2015). Geospatial Ecology of Adolescent Problem Behavior: Contributions of Community Factors and Parental Monitoring. Journal of community psychology, 42(3), 299–315. <https://doi.org/10.1002/jcop.21611>

Gerard, J. M., & Buehler, C. (2004). Cumulative environmental risk and youth problem behavior. *Journal of Marriage and Family*, *66*(3), 702–720. https://doi.org/10.1111/j.0022-2445.2004.00048.x

Germãn, M., Gonzales, N. A., & Dumka, L. (2009). Familism values as a protective factor for mexican-origin adolescents exposed to deviant peers. *Journal of Early Adolescence*, *29*(1), 16–42. https://doi.org/10.1177/0272431608324475

Gillis, B. T., & El-Sheikh, M. (2019). Sleep and adjustment in adolescence: physical activity as a moderator of risk. *Sleep Health*, *5*(3), 266–272. https://doi.org/10.1016/j.sleh.2019.02.001

Gómez-Leal, R., Megías-Robles, A., Gutiérrez-Cobo, M. J., Cabello, R., & Fernández-Berrocal, P. (2020). Personal Risk and Protective Factors Involved in Aggressive Behavior. *Journal of Interpersonal Violence*. https://doi.org/10.1177/0886260520926322

Goodrum, N. M., Chan, W. Y., & Latzman, R. D. (2015). Gang involvement among immigrant and refugee youth: A developmental ecological systems approach. *International Journal of Developmental Sciences*, *9*(3–4), 125–134. https://doi.org/10.3233/DEV-150164

Governale, A. (2019). *The role of multiple dimensions of organized activity participation, impulsivity and parental monitoring on externalizing behavior among low-income, urban adolescents* [Doctoral dissertation, Loyola University Chicago]. <https://ecommons.luc.edu/luc_diss/2958/>

Gresham, B., Orihuela, C. A., & Mrug, S. (2021). Moderating effects of coping style on externalizing behaviors and substance use in urban adolescents exposed to community violence. *International Journal of Developmental Science, 15*(3-4), 61-73. <https://doi.org/10.3233/DEV-210306>

Grych, J. H., Raynor, S. R., & Fosco, G. M. (2004). Family processes that shape the impact of interparental conflict on adolescents. *Development and Psychopathology*, *16*(3), 649–665. https://doi.org/10.1017/S0954579404004717

Guerrero, A. P. S., Hishinuma, E. S., Andrade, N. N., Nishimura, S. T., & Cunanan, V. L. (2006). Correlations among socioeconomic and family factors and academic, behavioral, and emotional difficulties in Filipino adolescents in Hawai’i. *International Journal of Social Psychiatry*, *52*(4), 343–359. https://doi.org/10.1177/0020764006065146

Gutiérrez-Cobo, M. J., Megías, A., Gómez-Leal, R., Cabello, R., & Fernández-Berrocal, P. (2018). The role of emotional intelligence and negative affect as protective and risk factors of aggressive behavior: A moderated mediation model. *Aggressive Behavior*, *44*(6), 638–646. https://doi.org/10.1002/ab.21788

Hall, C. W. (2006). Self-reported aggression and the perception of anger in facial expression photos. *Journal of Psychology: Interdisciplinary and Applied*, *140*(3), 255–267. https://doi.org/10.3200/JRLP.140.3.255-267

Hallfors, D., Cho, H., Brodish, P. H., Flewelling, R., & Khatapoush, S. (2006). Identifying high school students “at risk” for substance use and other behavioral problems: Implications for prevention. *Substance Use and Misuse*, *41*(1), 1–15. https://doi.org/10.1080/10826080500318509

Hanniball, K. B., Viljoen, J. L., Shaffer, C. S., Bhatt, G., Tweed, R., Aknin, L. B., ... & Dooley, S. (2021). The role of life satisfaction in predicting youth violence and offending: a prospective examination. *Journal of Interpersonal Violence, 36*(11-12), 5501-5529. <https://doi.org/10.1177/0886260518805103>

Hardaway, C. R., McLoyd, V. C., & Wood, D. (2012). Exposure to Violence and Socioemotional Adjustment in Low-Income Youth: An Examination of Protective Factors. *American Journal of Community Psychology*, *49*(1–2), 112–126. https://doi.org/10.1007/s10464-011-9440-3

Hardaway, C. R., Sterrett-Hong, E., Larkby, C. A., & Cornelius, M. D. (2016). Family Resources as Protective Factors for Low-Income Youth Exposed to Community Violence. *Journal of Youth and Adolescence*, *45*(7), 1309–1322. https://doi.org/10.1007/s10964-015-0410-1

Hart, D., Burock, D., London, B., Atkins, R., & Bonilla-Santiago, G. (2005). The relation of personality types to physiological, behavioural, and cognitive processes. *European Journal of Personality*, *19*(5), 391–407. https://doi.org/10.1002/per.547

Hemphill, S. A., Smith, R., Toumbourou, J. W., Herrenkohl, T. I., Catalano, R. F., McMorris, B. J., & Romaniuk, H. (2009). Modifiable determinants of youth violence in Australia and the United States: A longitudinal study. *Australian and New Zealand Journal of Criminology*, *42*(3), 289–309. https://doi.org/10.1375/acri.42.3.289

Henrich, C. C., Brookmeyer, K. A., & Shahar, G. (2005). Weapon violence in adolescence: Parent and school connectedness as protective factors. *Journal of Adolescent Health*, *37*(4), 306–312. https://doi.org/10.1016/j.jadohealth.2005.03.022

Henry, B., Caspi, A., Moffitt, T. E., Harrington, H. L., & Silva, P. A. (1999). Staying in school protects boys with poor self-regulation in childhood from later crime: A longitudinal study. *International Journal of Behavioral Development*, *23*(4), 1049–1073. https://doi.org/10.1080/016502599383667

Henry, D. B., Tolan, P. H., Gorman-Smith, D., & Schoeny, M. E. (2012). Risk and direct protective factors for youth violence: Results from the centers for disease control and prevention’s multisite violence prevention project. *American Journal of Preventive Medicine*, *43*, S67–S75. https://doi.org/10.1016/j.amepre.2012.04.025

Henry, J. S., Lambert, S. F., & Bynum, M. S. (2015). The protective role of maternal racial socialization for African American adolescents exposed to community violence. *Journal of Family Psychology*, *29*(4), 548–557. https://doi.org/10.1037/fam0000135

Herrenkohl, T. I., Hill, K. G., Chung, I., Guo, J., Abbott, R. D., & Hawkins, J. D. (2003). violent behavior in adolescence : A prospective study of aggressive children. *Social Work Research*, 179–191.

Herrenkohl, T. I., Tajima, E. A., Whitney, S. D., & Huang, B. (2005). Protection against antisocial behavior in children exposed to physically abusive discipline. *Journal of Adolescent Health*, *36*(6), 457–465. https://doi.org/10.1016/j.jadohealth.2003.09.025

Herrenkohl, T. I., Catalano, R. F., Hemphill, S. A., & Toumbourou, J. W. (2009). Longitudinal examination of physical and relational aggression as precursors to later problem behaviors in adolescents. *Violence and victims, 24*(1), 3–19. <https://doi.org/10.1891/0886-6708.24.1.3>

Hesse, C., Shorey, R. C., Brem, M. J., Stuart, G. L., & Cornelius, T. L. (2019). A Short-Term Longitudinal Investigation of the Relationship Between Trait Mindfulness and Female-Perpetrated Dating Violence. *Journal of Interpersonal Violence*. https://doi.org/10.1177/0886260519879264

Hill, J. M., Blokland, A. A. J., & van der Geest, V. R. (2018). Risk factors for self-reported delinquency in emerging adulthood. *European Journal of Criminology*, *15*(5), 544–566. https://doi.org/10.1177/1477370817749495

Hinnant, J. B., & Forman-Alberti, A. B. (2019). Deviant Peer Behavior and Adolescent Delinquency: Protective Effects of Inhibitory Control, Planning, or Decision Making? *Journal of Research on Adolescence*, *29*(3), 682–695. https://doi.org/10.1111/jora.12405

Hishinuma, E. S., Johnson, R. C., Kim, S. P., Nishimura, S. T., Makini, G. K., Andrade, N. N., Yates, A., Goebert, D. A., Mark, G. Y., Mayeda, D. T., & Revilla, L. A. (2005). Prevalence and correlates of misconduct among ethnically diverse adolescents of Native Hawaiian/part-hawaiian and non-hawaiian ancestry. *International Journal of Social Psychiatry*, *51*(3), 242–258. https://doi.org/10.1177/0020764005057371

Hishinuma, E. S., Umemoto, K. N., Nguyen, T. G., Chang, J. Y., & Bautista, R. P. M. (2012). Epidemiology of mixed martial arts and youth violence in an ethnically diverse sample. *Violence and Victims*, *27*(1), 43–69. https://doi.org/10.1891/0886-6708.27.1.43

Hofer, C., Eisenberg, N., & Reiser, M. (2010). The role of socialization, effortful control, and ego resiliency in French adolescents’ social functioning. *Journal of Research on Adolescence*, *20*(3), 555–582. https://doi.org/10.1111/j.1532-7795.2010.00650.x

Hope, M. O., & Buhs, E. (2020). Victimization and religious engagement: Links to school attachment and subsequent adjustment outcomes. *Psychology of Religion and Spirituality*, *12*(3), 334–344. https://doi.org/10.1037/rel0000232

Houtepen, J. A. B. M., Sijtsema, J. J., Klimstra, T. A., van der Lem, R., & Bogaerts, S. (2019). Loosening the Reins or Tightening Them? Complex Relationships Between Parenting, Effortful Control, and Adolescent Psychopathology. *Child and Youth Care Forum*, *48*(1), 127–145. https://doi.org/10.1007/s10566-018-9477-7

Huntington, J. S. (2014). Impact of exposure to violence on urban youth: A biopsychosocial perspective of aggression. *Dissertation Abstracts International: Section B: The Sciences and Engineering*, *75*(2-PG-). http://search.ebscohost.com/login.aspx?direct=true&db=psyh&AN=2014-99160-057&site=ehost-live NS  -

Ibabe, I. (2016). Academic failure and child-to-parent violence: Family protective factors. *Frontiers in Psychology*, *7*(OCT), 1–11. https://doi.org/10.3389/fpsyg.2016.01538

Ibabe, I., Jaureguizar, J., & Bentler, P. M. (2013). Protective factors for adolescent violence against authority. *Spanish Journal of Psychology*, *16*(2013), 1–13. https://doi.org/10.1017/sjp.2013.72

Ivanova, M. Y., & Israel, A. C. (2006). Family stability as a protective factor against psychopathology for urban children receiving psychological services. *Journal of Clinical Child and Adolescent Psychology*, *35*(4), 564–570. https://doi.org/10.1207/s15374424jccp3504_7

Jäggi, L., Kliewer, W., & Serpell, Z. (2020). Schooling while incarcerated as a turning point for serious juvenile and young adult offenders. *Journal of Adolescence, 78*, 9-23. <https://doi.org/10.1016/j.adolescence.2019.11.002>

Janssen, H. J., Weerman, F. M., & Eichelsheim, V. I. (2017). Parenting as a Protective Factor against Criminogenic Settings? Interaction Effects between Three Aspects of Parenting and Unstructured Socializing in Disordered Areas. *Journal of Research in Crime and Delinquency*, *54*(2), 181–207. https://doi.org/10.1177/0022427816664561

Jennings, W. G., Higgins, G. E., Tewksbury, R., Gover, A. R., & Piquero, A. R. (2010). A longitudinal assessment of the victim-offender overlap. *Journal of Interpersonal Violence*, *25*(12), 2147–2174. https://doi.org/10.1177/0886260509354888

Jessor, Richard and Van Den Bos, Jill and Vanderryn, Judith and Costa, Frances M. and Turbin, M. S. (1995). *Protective factors in adolescent problem behavior: Moderator effects and developmental change*.

Jiménez, T. I., Moreno-Ruiz, D., Estévez, E., Callejas-Jerónimo, J. E., López-Crespo, G., & Valdivia-Salas, S. (2021). Academic competence, teacher–student relationship, and violence and victimisation in adolescents: The classroom climate as a mediator. *International Journal of Environmental Research and Public Health, 18*(3), 1163. <https://doi.org/10.3390/ijerph18031163>

Johns, S., Song, E. J., Lee, J. M., Hong, J. S., Alexander, N. B., & Voisin, D. R. (2021). Caregivers’ Mental Illness and Adolescent Delinquency Among African Americans in Chicago’s Southside: Exploring Potential Protective Factors. *Child and Adolescent Social Work Journal, 38*, 621-630. <https://doi.org/10.1007/s10560-020-00685-1>

Johnson, S. A. (2005). *The relationship of parenting with adolescent problem behaviors and healthy development: An application of a motivational model of development* [Doctoral dissertation, Portland State University]. [*https://pdxscholar.library.pdx.edu/open_access_etds/4265/*](https://pdxscholar.library.pdx.edu/open_access_etds/4265/)

Jolliffe, D., Farrington, D. P., Loeber, R., & Pardini, D. (2016). Protective factors for violence: Results from the Pittsburgh Youth Study. *Journal of Criminal Justice*, *45*, 32–40. https://doi.org/10.1016/j.jcrimjus.2016.02.007

Jones, N. J. (2011). *Merging theoretical frameworks to inform risk assessment for the young female offender* [Doctoral dissertation, Carleton University]. <https://curve.carleton.ca/system/files/etd/6acb828d-7c13-4493-b2ef-0d03ca67301c/etd_pdf/82710f5be28b212bfb24695fa1bc893e/jones-mergingtheoreticalframeworkstoinformriskassessment.pdf>

Jung, J., & Schröder-Abé, M. (2019). Prosocial behavior as a protective factor against peers’ acceptance of aggression in the development of aggressive behavior in childhood and adolescence. *Journal of Adolescence*, *74*(June), 146–153. https://doi.org/10.1016/j.adolescence.2019.06.002

Kalaitzaki, A. E. (2019). The Pathway from Family Violence to Dating Violence in College Students’ Relationships: A Multivariate Model. *Journal of Aggression, Maltreatment and Trauma*, *28*(3), 313–333. https://doi.org/10.1080/10926771.2018.1515137

Kapetanovic, S., & Skoog, T. (2021). The Role of the Family's Emotional Climate in the Links between Parent-Adolescent Communication and Adolescent Psychosocial Functioning. *Research on Child and Adolescent Psychopathology, 49*(2), 141–154. <https://doi.org/10.1007/s10802-020-00705-9>

Kaufmann, D. R. (2008). *Resilience, resources, and protective processes in the context of low youth-parent bonding* (Publication No. 3314683) [Doctoral dissertation, University of Rochester]. ProQuest Dissertations Publishing.

Kaufmann, D. R., Wyman, P. A., Forbes-Jones, E. L., & Barry, J. (2007). Prosocial involvement and antisocial peer affiliations as predictors of behavior problems in urban adolescents: Main effects and moderating effects. *Journal of Community Psychology*, *35*(4), 417–434. https://doi.org/10.1002/jcop.20156

Kauten, R., Barry, C. T., & Leachman, L. (2013). Do perceived social stress and resilience influence the effects of psychopathy-linked narcissism and CU traits on adolescent aggression? *Aggressive Behavior*, *39*(5), 381–390. https://doi.org/10.1002/ab.21483

Kendler, K. S., Lönn, S. L., Lichtenstein, P., Sundquist, J., & Sundquist, K. (2016). Psychological strength assessed in late adolescence and risk for criminal behavior: A Swedish prospective cohort and twin analysis. *Psychological Medicine*, *46*(1), 63–72. https://doi.org/10.1017/S0033291715001403

Khafi, T. Y., Yates, T. M., & Luthar, S. S. (2014). Ethnic differences in the developmental significance of parentification. *Family Process*, *53*(2), 267–287. https://doi.org/10.1111/famp.12072

Khurana, A., Bleakley, A., Ellithorpe, M. E., Hennessy, M., Jamieson, P. E., & Weitz, I. (2019). Media violence exposure and aggression in adolescents: A risk and resilience perspective. *Aggressive Behavior*, *45*(1), 70–81. https://doi.org/10.1002/ab.21798

Kim-Ju, G. M., Goodman, Z. T., & Her, S. (2018). Community violence exposure and internalizing and externalizing behaviors among Hmong Americans. *Asian American Journal of Psychology, 9*(2), 87–97. https://doi.org/10.1037/aap0000089

Kincaid, C. Y. (2012). *Risk & resilience among African American single mother families: A closer look at parenting & adolescent outcomes* [Doctoral dissertation, University of North Carolina]. <https://cdr.lib.unc.edu/concern/dissertations/w6634415g?locale=en>

Kingsbury, M., Clayborne, Z., Colman, I., & Kirkbride, J. B. (2020). The protective effect of neighbourhood social cohesion on adolescent mental health following stressful life events. *Psychological Medicine*, *50*(8), 1292–1299. https://doi.org/10.1017/S0033291719001235

Kokko, K., Tremblay, R. E., Lacourse, E., Nagin, D. S., & Vitaro, F. (2006). Trajectories of prosocial behavior and physical aggression in middle childhood: Links to adolescent school dropout and physical violence. *Journal of Research on Adolescence*, *16*(3), 403–428. https://doi.org/10.1111/j.1532-7795.2006.00500.x

Korol, L., & Bevelander, P. (2021). Ethnic Harassment and the Protective Effect of Positive Parenting on Immigrant Youths’ Antisocial Behavior. *Child & Youth Care Forum,* *50*, 805–826

Kosterman, R., Hawkins, J. D., Abbott, R. D., Hill, K. G., Herrenkohl, T. I., & Catalano, R. F. (2005). Measures of positive adult behavior and their relationship to crime and substance use. *Prevention Science*, *6*(1), 21–33. https://doi.org/10.1007/s11121-005-1250-0

Kramer-Kuhn, A. M., & Farrell, A. D. (2016). The Promotive and Protective Effects of Family Factors in the Context of Peer and Community Risks for Aggression. *Journal of Youth and Adolescence*, *45*(4), 793–811. https://doi.org/10.1007/s10964-016-0438-x

Kuhn, M. A. (2019). *Attachment and internalizing and externalizing problems in adolescence: Exploring the mediating role of physiological self-regulation capacity* [Doctoral dissertation, Seattle Pacific University]. [*https://digitalcommons.spu.edu/cgi/viewcontent.cgi?article=1041&context=cpy_etd*](https://digitalcommons.spu.edu/cgi/viewcontent.cgi?article=1041&context=cpy_etd)

Kuruczova, D., Klanova, J., Jarkovsky, J., Pikhart, H., & Bienertova-Vasku, J. (2020). Socioeconomic characteristics, family structure and trajectories of children’s psychosocial problems in a period of social transition. *PLoS ONE*, *15*(6), 1–16. https://doi.org/10.1371/journal.pone.0234074

Lagasse, L. L., Conradt, E., Karalunas, S. L., Dansereau, L. M., Butner, J. E., Shankaran, S., Bada, H., Bauer, C. R., Whitaker, T. M., & Lester, B. M. (2016). Transactional relations between caregiving stress, executive functioning, and problem behavior from early childhood to early adolescence. *Development and Psychopathology*, *28*(3), 743–756. https://doi.org/10.1017/S0954579416000286

Laird, R. D., Marks, L. D., & Marrero, M. D. (2011). Religiosity, self-control, and antisocial behavior: Religiosity as a promotive and protective factor. *Journal of Applied Developmental Psychology*, *32*(2), 78–85. https://doi.org/10.1016/j.appdev.2010.12.003

Lansford, J. E., Malone, P. S., Stevens, K. I., Dodge, K. A., Bates, J. E., & Pettit, G. S. (2006). Developmental trajectories of externalizing and internalizing behaviors: Factors underlying resilience in physically abused children. *Development and Psychopathology*, *18*(1), 35–55. https://doi.org/10.1017/S0954579406060032

Lantagne, A., & Furman, W. (2019). More Than the Sum of Two Partners: A Dyadic Perspective on Young Adult Physical Dating Aggression. *Psychology of Violence*, *10*(4), 379–389. https://doi.org/10.1037/vio0000267

Le, T. N., & Johansen, S. (2011). The Relationship Between School Multiculturalism and Interpersonal Violence: An Exploratory Study. *Journal of School Health*, *81*(11), 688–695. https://doi.org/10.1111/J.1746-1561.2011.00645.X

Leadbeater, B. J., Banister, E. M., Ellis, W. E., & Yeung, R. (2008). Victimization and relational aggression in adolescent romantic relationships: The influence of parental and peer behaviors, and individual adjustment. *Journal of Youth and Adolescence*, *37*(3), 359–372. https://doi.org/10.1007/s10964-007-9269-0

Legault, L., Anawati, M., & Flynn, R. (2006). Factors favoring psychological resilience among fostered young people. *Children and Youth Services Review*, *28*(9), 1024–1038. https://doi.org/10.1016/j.childyouth.2005.10.006

Linver, M. R., Roth, J. L., & Brooks-Gunn, J. (2009). Patterns of Adolescents’ Participation in Organized Activities: Are Sports Best When Combined With Other Activities? *Developmental Psychology*, *45*(2), 354–367. https://doi.org/10.1037/a0014133

Liu, J., Mustanski, B., Dick, D., Bolland, J., & Kertes, D. A. (2017). Risk and protective factors for comorbid internalizing and externalizing problems among economically disadvantaged African American youth. *Development and psychopathology, 29*(3), 1043–1056. <https://doi.org/10.1017/S0954579416001012>

Liu, L., & Miller, S. L. (2020). Protective factors against juvenile delinquency: Exploring gender with a nationally representative sample of youth. *Social Science Research*, *86*, 102376. <https://doi.org/10.1016/j.ssresearch.2019.102376>

Livingston, J. A., Eiden, R. D., Lessard, J., Casey, M., Henrie, J., & Leonard, K. E. (2018). Etiology of Teen Dating Violence among Adolescent Children of Alcoholics. *Journal of Youth and Adolescence*, *47*(3), 515–533. https://doi.org/10.1007/s10964-017-0730-4

Lloyd, K. M. (2021). *Changing with the Times? A Generational Comparison of the Effects Parental Social Ties on Crime and Drug Use During Emerging Adulthood* [Doctoral dissertation]*.* The Florida State University.

Longobardi, C., Prino, L. E., Marengo, D., & Settanni, M. (2016). Student-teacher relationships as a protective factor for school adjustment during the transition from middle to high school. *Frontiers in Psychology*, *7*, 1–9. https://doi.org/10.3389/fpsyg.2016.01988

Loukas, A., Prelow, H. M., Suizzo, M. A., & Allua, S. (2008). Mothering and peer associations mediate cumulative risk effects for latino youth. *Journal of Marriage and Family*, *70*(1), 76–85. https://doi.org/10.1111/j.1741-3737.2007.00462.x

Low, S., Polanin, J. R., & Espelage, D. L. (2013). The Role of Social Networks in Physical and Relational Aggression Among Young Adolescents. *Journal of Youth and Adolescence*, *42*(7), 1078–1089. https://doi.org/10.1007/s10964-013-9933-5

Lyons, M. D., Otis, K. L., Scott Huebner, E., & Hills, K. J. (2014). Life satisfaction and maladaptive behaviors in early adolescents. *School Psychology Quarterly*, *29*(4), 553–566. https://doi.org/10.1037/spq0000061

Machell, K. A., Disabato, D. J., & Kashdan, T. B. (2016). Buffering the Negative Impact of Poverty on Youth: The Power of Purpose in Life. *Social Indicators Research*, *126*(2), 845–861. https://doi.org/10.1007/s11205-015-0917-6

Mahoney, J. L., & Vest, A. E. (2012). The over-scheduling hypothesis revisited: Intensity of organized activity participation during adolescence and young adult outcomes. *Journal of Research on Adolescence*, *22*(3), 409–418. https://doi.org/10.1111/j.1532-7795.2012.00808.x

Manzi, C., Ferrari, L., Rosnati, R., & Benet-Martinez, V. (2014). Bicultural Identity Integration of Transracial Adolescent Adoptees: Antecedents and Outcomes. *Journal of Cross-Cultural Psychology*, *45*(6), 888–904. https://doi.org/10.1177/0022022114530495

Marsiglia, F. F., Parsai, M., & Stephen, K. (2009). Effects of familism and family cohesion on poblem behaviors among adolescents in Mexican immigrant families in the Southwest United States. *Journal of Ethnic and Cultural Diversity in Social Work*, *18*(3), 203–220. https://doi.org/10.1080/15313200903070965

Marston, E. G. (2011). *Rejection sensitivity in adolescence: Peer-related precursors and links with problem behaviors in early adulthood*.

Mattson, S. A., Sigel, E., & Mercado, M. C. (2020). Risk and Protective Factors Associated with Youth Firearm Access, Possession or Carrying. *American Journal of Criminal Justice, 45*(5), 844–864. <https://doi.org/10.1007/s12103-020-09521-9>

Mauer, V. A., Waterman, E. A., Edwards, K. M., & Banyard, V. L. (2022). Adolescents' Relationships With Important Adults: Exploring This Novel Protective Factor Against Interpersonal Violence Victimization and Perpetration. *Journal of Interpersonal Violence, 37*, 19-20. <https://doi.org/10.1177/08862605211031252>

McGee, Z. (2015). Risk, Protective Factors, and Symptomatology Among Urban Adolescents: Findings of a Research Study on Youth Violence and Victimization. *Journal of Offender Rehabilitation*, *54*(6), 429–444. https://doi.org/10.1080/10509674.2015.1056902

McGee, Z. T. (2003). Community Violence and Adolescent Development. *Journal of Contemporary Criminal Justice*, *19*(3), 293–314. https://doi.org/10.1177/1043986203254527

McKelvey, L. M., Conners-Burrow, N. A., Mesman, G. R., Pemberton, J. R., & Casey, P. H. (2015). Promoting Adolescent Behavioral Adjustment in Violent Neighborhoods: Supportive Families Can Make a Difference! *Journal of Clinical Child and Adolescent Psychology*, *44*(1), 157–168. https://doi.org/10.1080/15374416.2014.895939

McMahon, S. D., Todd, N. R., Martinez, A., Coker, C., Sheu, C. F., Washburn, J., & Shah, S. (2013). Aggressive and Prosocial Behavior: Community Violence, Cognitive, and Behavioral Predictors Among Urban African American Youth. *American Journal of Community Psychology*, *51*(3–4), 407–421. https://doi.org/10.1007/s10464-012-9560-4

Meltzer, H. I., Dogra, N., Vostanis, P., & Ford, T. (2011). Religiosity and the mental health of adolescents in Great Britain. *Mental Health, Religion and Culture*, *14*(7), 703–713. https://doi.org/10.1080/13674676.2010.515567

Mihalec-Adkins, B. P., & Cooley, M. E. (2020). Examining individual-level academic risk and protective factors for foster youth: School engagement, behaviors, self-esteem, and social skills. *Child and Family Social Work*, *25*(2), 256–266. https://doi.org/10.1111/cfs.12681

Mikami, A. Y., & Hinshaw, S. P. (2006). Resilient adolescent adjustment among girls: Buffers of childhood peer rejection and attention-deficit/hyperactivity disorder. *Journal of Abnormal Child Psychology*, *34*(6), 825–839. https://doi.org/10.1007/s10802-006-9062-7

Mirza, F. Y. (2015). *Understanding american muslim youth of arab and south asian ancestries: An exploratory study on the factors related to risk behaviors among child immigrants and children of immigrants* [Doctoral dissertation, University of Maryland]. <https://archive.hshsl.umaryland.edu/handle/10713/4408>

Mitchell, D. B., Szczerepa, A., & Hauser-Cram, P. (2016). Spilling over: Partner parenting stress as a predictor of family cohesion in parents of adolescents with developmental disabilities. *Research in Developmental Disabilities*, *49–50*, 258–267. https://doi.org/10.1016/j.ridd.2015.12.007

Mohammad, E. T., Shapiro, E. R., Wainwright, L. D., & Carter, A. S. (2014). Impacts of Family and Community Violence Exposure on Child Coping and Mental Health. *Journal of Abnormal Child Psychology*, *43*(2), 203–215. https://doi.org/10.1007/s10802-014-9889-2

Monahan, J., Steadman, H. J., Appelbaum, P. S., Robbins, P. C., Mulvey, E. P., Silver, E., Roth, L. H., & Grisso, T. (2000). Developing a clinically useful actuarial tool for assessing violence risk. *British Journal of Psychiatry*, *176*(APR.), 312–319. https://doi.org/10.1192/bjp.176.4.312

Morimoto, Y., & Sharma, A. (2004). Long-term outcomes of verbal aggression: The role of protective factors. *Journal of Emotional Abuse*, *4*(2), 71–99. https://doi.org/10.1300/J135v04n02_04

Moyer, V. S. (2008). *The role of user motivations in moderating the relation between video game playing and children’s adjustment* [Doctoral dissertation, Bowling Green State University]. <https://etd.ohiolink.edu/apexprod/rws_olink/r/1501/10?clear=10&p10_accession_num=bgsu1210548781>

Munir, A., & Malik, J. A. (2020). Mediating role of religious orientation and moral character for the relationship between parent and peer attachment and delinquency. *Cogent Psychology*, *7*(1), 1–15. https://doi.org/10.1080/23311908.2020.1761042

Neaverson, A., Murray, A. L., Ribeaud, D., & Eisner, M. (2020). A Longitudinal Examination of the Role of Self-Control in the Relation between Corporal Punishment Exposure and Adolescent Aggression. *Journal of Youth and Adolescence*, *49*(6), 1245–1259. https://doi.org/10.1007/s10964-020-01215-z

Okeem, C. (2018). *A Theoretical Exploration of Psychological Grit in the Context of Criminology* [Doctoral dissertation, The University of Texas]*.* <https://utd-ir.tdl.org/bitstream/handle/10735.1/5902/ETD-5608-012-OKEEM-7924.59.pdf?sequence=5&isAllowed=y>

Oliva, A., Jiménez, J. M., & Parra, Á. (2009). Protective effect of supportive family relationships and the influence of stressful life events on adolescent adjustment. *Anxiety, Stress and Coping*, *22*(2), 137–152. https://doi.org/10.1080/10615800802082296

Oliver, B. R., & Hodgins, S. (2013). Understanding violence in girls with substance misuse problems. In Andershed, A.-K (Ed.), *Girls at risk: Swedish longitudinal research on adjustment* (pp. 79–104). Springer Science.

Oshri, A., Rogosch, F. A., & Cicchetti, D. (2013). Child Maltreatment and Mediating Influences of Childhood Personality Types on the Development of Adolescent Psychopathology. *Journal of Clinical Child and Adolescent Psychology*, *42*(3), 287–301. https://doi.org/10.1080/15374416.2012.715366

Oudekerk, B. A., Chauhan, P., & Reppucci, N. D. (2012). Risk and Protective Factors for Educational Achievement Among Serious Offending Girls. *Journal of Research on Adolescence*, *22*(2), 333–349. https://doi.org/10.1111/j.1532-7795.2011.00771.x

Pardini, D. A., Loeber, R., Farrington, D. P., & Stouthamer-Loeber, M. (2012). Identifying direct protective factors for nonviolence. *American Journal of Preventive Medicine*, *43*, S28–S40. https://doi.org/10.1016/j.amepre.2012.04.024

Pastor, Y. (2020). Psychosocial determinants of depression and maladaptive behaviour in adolescence: two tested models. *Journal of Child and Adolescent Mental Health*, *32*(1), 11–22. https://doi.org/10.2989/17280583.2019.1698430

Paterson, J., Prah, P., Tautolo, E. S., & Iusitini, L. (2022). Pacific Islands Families Study: Patterns of Intimate Partner Violence Among Pacific Mothers. *Journal of Interpersonal Violence, 37*(5-6), 2598–2614. <https://doi.org/10.1177/0886260520938510>

Paysnick, A. A., & Burt, K. B. (2015). Moderating Effects of Coping on Associations between Autonomic Arousal and Adolescent Internalizing and Externalizing Problems. *Journal of Clinical Child and Adolescent Psychology*, *44*(5), 846–858. https://doi.org/10.1080/15374416.2014.891224

Pearce, M. J., Jones, S. M., Schwab-Stone, M. E., & Ruchkin, V. (2003). The Protective Effects of Religiousness and Parent Involvement on the Development of Conduct Problems among Youth Exposed to Violence. *Child Development*, *74*(6), 1682–1696. https://doi.org/10.1046/j.1467-8624.2003.00631.x

Perryman, T. L. (2016). *Examining the associations between racial socialization and violence initiation: Implications for urban, Black young adults* [Doctoral dissertation, Ohio State University]. https://etd.ohiolink.edu/apexprod/rws_olink/r/1501/10?clear=10&p10_accession_num=osu1468496537

Petit, W. E., Knee, C. R., Hadden, B. W., & Rodriguez, L. M. (2017). Self-determination theory and intimate partner violence: An APIM model of need fulfillment and IPV. *Motivation Science*, *3*(2), 119–132. https://doi.org/10.1037/mot0000054

Pierre, C. L. (2016). *Community violence exposure among urban African American males: Understanding the “buffering effect” of adaptive social support coping on psychosocial outcomes* [Doctoral dissertation, Loyola University Chicago]. <https://ecommons.luc.edu/luc_diss/2143/>

Pierre, C. L., Burnside, A., & Gaylord-Harden, N. K. (2020). A Longitudinal Examination of Community Violence Exposure, School Belongingness, and Mental Health Among African-American Adolescent Males. *School Mental Health*, *12*(2), 388–399. https://doi.org/10.1007/s12310-020-09359-w

Powell, T. W., Latimore, A., Fuentes, L. V. M., Marshall, B. D., Mendelson, T., & Tandon, S. D. (2021). Maternal Support and Physical Dating Violence Perpetration among Disconnected Young Women. *Journal of Child and Family Studies, 30*, 611-619. <https://doi.org/10.1007/s10826-020-01878-z>

Preece, K. K. (2015). *Risk and Resiliency Factors at School Entry: Relations to Academic and Behavioral Outcomes in Early Adolescence* [Doctoral dissertation, University of South Florida]. <https://digitalcommons.usf.edu/cgi/viewcontent.cgi?article=7212&context=etd>

Prelow, H. M., Bowman, M. A., & Weaver, S. R. (2007). Predictors of psychosocial well-being in Urban African American and European American youth: The role of ecological factors. *Journal of Youth and Adolescence*, *36*(4), 543–553. https://doi.org/10.1007/s10964-006-9038-5

Prince, D. M., Vidal, S., Okpych, N., & Connell, C. M. (2019). Effects of individual risk and state housing factors on adverse outcomes in a national sample of youth transitioning out of foster care. *Journal of Adolescence*, *74*(May), 33–44. https://doi.org/10.1016/j.adolescence.2019.05.004

Puckett, T. (2011). *The influence of risk and protective factors on health-compromising behaviors among incarcerated juveniles* [Doctoral dissertation, University of Akron]*.* https://etd.ohiolink.edu/apexprod/rws_etd/send_file/send?accession=akron1279594086&disposition=inline

Rapp-Paglicci, L., Stewart, C., & Rowe, W. (2011). Can a self-regulation skills and cultural arts program promote positive outcomes in mental health symptoms and academic achievement for at-risk youth? *Journal of Social Service Research*, *37*(3), 309–319. https://doi.org/10.1080/01488376.2011.564067

Reardon, K. W., Herzhoff, K., & Tackett, J. L. (2016). Adolescent Personality as Risk and Resiliency in the Testosterone–Externalizing Association. *Journal of Research on Adolescence*, *26*(3), 390–402. https://doi.org/10.1111/jora.12198

Reingle, J. M., Jennings, W. G., & Komro, K. A. (2013). A case-control study of risk and protective factors for incarceration among urban youth. *Journal of Adolescent Health*, *53*(4), 471–477. https://doi.org/10.1016/j.jadohealth.2013.05.008

Resnick, M. D., Bearman, P. S., Blum, R. W., Bauman, K. E., Harris, K. M., Jones, J., Tabor, J., Beuhring, T., Sieving, R. E., Shew, M., Ireland, M., Bearinger, L. H., & Udry, J. R. (1997). Protecting adolescents from harm. Findings from the National Longitudinal Study on Adolescent Health. *JAMA, 278*(10), 823–832. <https://doi.org/10.1001/jama.278.10.823>

Richardson, P. A., Sala-Hamrick, K., Simon, V., & Barnett, D. (2019). Secure Base Representations among Inner-City Adolescents. *Journal of Child and Family Studies*, *28*(3), 784–795. https://doi.org/10.1007/s10826-018-01312-5

Rious, J. B., & Cunningham, M. (2018). Altruism as a buffer for antisocial behavior for African American adolescents exposed to community violence. *Journal of Community Psychology*, *46*(2), 224–237. https://doi.org/10.1002/jcop.21936

Roberts, D. M. (2017). *Resilience in Physically Maltreated Adolescents : Interpersonally Related Protective Factors and Gender Differences* [Doctoral dissertation, the George Washington University]. <https://www.proquest.com/docview/1881314883?pq-origsite=gscholar&fromopenview=true>

Roberts, R. E., Roberts, C. R., & Chan, W. (2009). One-year incidence of psychiatric disorders and associated risk factors among adolescents in the community. *Journal of Child Psychology and Psychiatry and Allied Disciplines*, *50*(4), 405–415. https://doi.org/10.1111/j.1469-7610.2008.01969.x

Romont, L. A. (2012). *Resiliency with pre-adjudicated females* [Doctoral dissertation, University of Northern Colorado]. <https://digscholarship.unco.edu/cgi/viewcontent.cgi?article=1241&context=dissertations>

Rosky, J. W. (2010). *Examining the relationship between risk, protection, self-control and resilience* [Doctoral dissertation].

Ross, K. M., Walsh, C. S., O'Connor, K. E., & Sullivan, T. N. (2023). Ecological promotive and protective factors deterring gun carriage for young adults living in communities with high rates of community violence. *Journal of Community Psychology, 51*(3), 1164-1180. <https://doi.org/10.1002/jcop.23008>

Salas-Wright, C. P., Tirmazi, T., Lombe, M., & Nebbitt, V. E. (2015a). Religiosity and antisocial behavior: Evidence from young African American women in public housing communities. *Social Work Research*, *39*(2), 82–93. https://doi.org/10.1093/swr/svv010

Salas-Wright, C. P., Vaughn, M. G., & Maynard, B. R. (2015b). Profiles of Religiosity and Their Association With Risk Behavior Among Emerging Adults in the United States. *Emerging Adulthood*, *3*(2), 67–84. https://doi.org/10.1177/2167696814539327

Salzinger, S., Feldman, R. S., Rosario, M., & Ng-Mak, D. S. (2011). Role of parent and peer relationships and individual characteristics in middle school children’s behavioral outcomes in the face of community violence. *Journal of Research on Adolescence*, *21*(2), 395–407. https://doi.org/10.1111/j.1532-7795.2010.00677.x

Sasser, J., Duprey, E. B., & Oshri, A. (2019). A longitudinal investigation of protective factors for bereaved maltreated youth. *Child Abuse and Neglect*, *96*, 104135. https://doi.org/10.1016/j.chiabu.2019.104135

Scarpa, A., & Haden, S. C. (2006). Community violence victimization and aggressive behavior: The moderating effects of coping and social support. Aggressive Behavior: *Official Journal of the International Society for Research on Aggression, 32*(5), 502-515.

Schmidt, J. A., Shumow, L., & Kackar, H. Z. (2012). Associations of Participation in Service Activities with Academic, Behavioral, and Civic Outcomes of Adolescents at Varying Risk Levels. *Journal of Youth and Adolescence*, *41*(7), 932–947. https://doi.org/10.1007/s10964-011-9694-y

Schnurr, M. P. (2009). *Precursors to adolescents’ dating violence perpetration and healthy romantic relationships* [Doctoral dissertation, Iowa State University]. <https://dr.lib.iastate.edu/server/api/core/bitstreams/eece0878-bdcd-49b4-a264-b387da0605a6/content>

Schriber, R. A., Rogers, C. R., Ferrer, E., Conger, R. D., Robins, R. W., Hastings, P. D., & Guyer, A. E. (2018). Do Hostile School Environments Promote Social Deviance by Shaping Neural Responses to Social Exclusion? *Journal of Research on Adolescence*, *28*(1), 103–120. https://doi.org/10.1111/jora.12340

Scott, T., Brown, S. l., & Skilling, T. A. (2019). Predictive and Convergent Validity of the Youth Assessment and Screening Instrument in a Sample of Male and Female Justice-Involved Youth. *Criminal Justice and Behavior*, *46*(6), 811–831. https://doi.org/10.1177/0093854819842585

Segura, A., Pereda, N., Guilera, G., & Hamby, S. (2017). Resilience and psychopathology among victimized youth in residential care. *Child Abuse and Neglect*, *72*(September), 301–311. https://doi.org/10.1016/j.chiabu.2017.08.019

Sharma, S., Mustanski, B., Dick, D., Bolland, J., & Kertes, D. A. (2019). Protective Factors Buffer Life Stress and Behavioral Health Outcomes among High-Risk Youth. *Journal of Abnormal Child Psychology*, *47*(8), 1289–1301. https://doi.org/10.1007/s10802-019-00515-8

Shorey, R. C., Elmquist, J. A., Anderson, S., & Stuart, G. L. (2016). The Relationship Between Spirituality and Aggression in a Sample of Men in Residential Substance Use Treatment. *International Journal of Mental Health and Addiction*, *14*(1), 23–30. https://doi.org/10.1007/s11469-015-9565-y

Shorey, R. C., Elmquist, J. A., Zucosky, H., Febres, J., Brasfield, H., & Stuart, G. L. (2014). Experiential avoidance and male dating violence perpetration: An initial investigation. *Journal of Contextual Behavioral Science*, *3*(2), 117–123. https://doi.org/10.1016/j.jcbs.2014.02.003

Showalter, K., Maguire-Jack, K., & Barnhart, S. (2017). Investigating the Potentially Protective Effects of Neighborhood Processes in Intimate Partner Violence. *Journal of Aggression, Maltreatment and Trauma*, *26*(10), 1090–1103. https://doi.org/10.1080/10926771.2017.1320345

Smokowski, P. R., Cotter, K. L., Robertson, C. I. B., & Guo, S. (2013). Anxiety and aggression in rural youth: Baseline results from the rural adaptation project. *Child Psychiatry and Human Development*, *44*(4), 479–492. https://doi.org/10.1007/s10578-012-0342-x

So, S., Gaylord-Harden, N. K., Voisin, D. R., & Scott, D. (2018). Future Orientation as a Protective Factor for African American Adolescents Exposed to Community Violence. *Youth and Society*, *50*(6), 734–757. https://doi.org/10.1177/0044118X15605108

Soderstrom, M. F., Childs, K. K., & Frick, P. J. (2020). The role of protective factors in the predictive accuracy of the structured assessment of violence risk in youth (SAVRY). *Youth Violence and Juvenile Justice, 18*(1), 78-95. <https://doi.org/10.1177/1541204019837329>

Spano, R., Vazsonyi, A. T., & Bolland, J. (2009). Does parenting mediate the effects of exposure to violence on violent behavior? An ecological-transactional model of community violence. *Journal of Adolescence*, *32*(5), 1321–1341. https://doi.org/10.1016/j.adolescence.2008.12.003

Sparks, L. A., Trentacosta, C. J., Hicks, M. R., Kernsmith, P., & Smith-Darden, J. (2021). Hope as a protective factor: Relations to adverse childhood experiences, delinquency, and posttraumatic stress symptoms. *Journal of Child and Family Studies, 30*, 3005-3015. https://doi.org/10.1007/s10826-021-02119-7

Spice, A., Viljoen, J. L., Gretton, H. M., & Roesch, R. (2010). Psychological assessment for adult sentencing of juvenile offenders: An evaluation of the RSTI and the SAVRY. *International Journal of Forensic Mental Health*, *9*(2), 124–137. https://doi.org/10.1080/14999013.2010.501846

Steiner, R. J., Sheremenko, G., Lesesne, C., DIttus, P. J., Sieving, R. E., & Ethier, K. A. (2019). Adolescent connectedness and adult health outcomes. *Pediatrics*, *144*(1). https://doi.org/10.1542/peds.2018-3766

Stewart, C., & Rapp, L. (2017). The Relationship of Spirituality and Family Functioning to Recidivism: An Investigation with Incarcerated Adolescent Males. *Residential Treatment for Children and Youth*, *34*(3–4), 292–310. https://doi.org/10.1080/0886571X.2017.1370409

Stoddard, S. A., Heinze, J. E., Choe, D. E., & Zimmerman, M. A. (2015). Predicting violent behavior: The role of violence exposure and future educational aspirations during adolescence. *Journal of Adolescence*, *44*, 191–203. https://doi.org/10.1016/j.adolescence.2015.07.017

Stoddard, S. A., McMorris, B. J., & Sieving, R. E. (2011). Do Social Connections and Hope Matter in Predicting Early Adolescent Violence? *American Journal of Community Psychology*, *48*(3–4), 247–256. https://doi.org/10.1007/s10464-010-9387-9

Stoeckel, A. H. (2011). *Evaluating the role of perceived access to support and school safety in adolescent exposure to trauma: A mixed methods study* [Doctoral dissertation, University of Northern Colorado]. <https://digscholarship.unco.edu/dissertations/258/>

Stouthamer-Loeber, M., Wei, E., Loeber, R., & Masten, A. S. (2004). Desistance from persistent serious delinquency in the transition to adulthood. *Development and Psychopathology*, *16*(4), 897–918. https://doi.org/10.1017/S0954579404040064

Stuewig, J., Tangney, J. P., Heigel, C., Harty, L., & McCloskey, L. (2010). Shaming, blaming, and maiming: Functional links among the moral emotions, externalization of blame, and aggression. *Journal of Research in Personality*, *44*(1), 91–102. https://doi.org/10.1016/j.jrp.2009.12.005

Svetaz, M. V., Ireland, M., & Blum, R. (2000). Adolescents with learning disabilities: Risk and protective factors associated with emotional well-being: findings from the National Longitudinal Study of Adolescent Health. *Journal of Adolescent Health*, *27*(5), 340–348. https://doi.org/10.1016/S1054-139X(00)00170-1

Szwedo, D. E., Hessel, E. T., & Allen, J. P. (2017). Supportive Romantic Relationships as Predictors of Resilience Against Early Adolescent Maternal Negativity. *Journal of Youth and Adolescence*, *46*(2), 454–465. https://doi.org/10.1007/s10964-016-0507-1

Talley, L. M. (2019). *Exploring diverse profiles of identity, risk taking, and health risk in urban Black emerging adult men* [Doctoral dissertation, University of Pennsylvania]*.* https://repository.upenn.edu/dissertations/AAI10979846/

Taubner, S., White, L. O., Zimmermann, J., Fonagy, P., & Nolte, T. (2013). Attachment-related mentalization moderates the relationship between psychopathic traits and proactive aggression in adolescence. *Journal of Abnormal Child Psychology*, *41*(6), 929–938. https://doi.org/10.1007/s10802-013-9736-x

Taubner, S., Zimmermann, L., Ramberg, A., & Schröder, P. (2016). Mentalization Mediates the Relationship between Early Maltreatment and Potential for Violence in Adolescence. *Psychopathology*, *49*(4), 236–246. https://doi.org/10.1159/000448053

Taussig, H. N. (2002). Risk behaviors in maltreated youth placed in foster care: A longitudinal study of protective and vulnerability factors. *Child Abuse and Neglect*, *26*(11), 1179–1199. https://doi.org/10.1016/S0145-2134(02)00391-5

Taylor, R. D. (2010). Risk and resilience in low-income african american families: Moderating effects of kinship social support. *Cultural Diversity and Ethnic Minority Psychology*, *16*(3), 344–351. https://doi.org/10.1037/a0018675

Thomas, A. (2013). *Bad boys of bad odds? - race, context, and social influence: An investigation of youth violence in African-American boys* [Doctoral dissertation, The University of Michigan]. <https://deepblue.lib.umich.edu/bitstream/handle/2027.42/98054/althoma_1.pdf?sequence=1&isAllowed=y>

Thomas, A., & Hope, E. C. (2016). Walking Away Hurt, Walking Around Scared: A Cluster Analysis of Violence Exposure Among Young Black Males. *Journal of Black Psychology*, *42*(5), 453–476. https://doi.org/10.1177/0095798415603539

Tiềt, Q. Q., & Huizinga, D. (2002). Dimensions of the construct of resilience and adaptation among inner-city youth. *Journal of Adolescent Research*, *17*(3), 260–276. https://doi.org/10.1177/0743558402173003

Tomeny, T. S., Barry, T. D., & Bader, S. H. (2012). Are typically-developing siblings of children with an autism spectrum disorder at risk for behavioral, emotional, and social maladjustment? *Research in Autism Spectrum Disorders*, *6*(1), 508–518. https://doi.org/10.1016/j.rasd.2011.07.012

Topitzes, J., Mersky, J. P., Dezen, K. A., & Reynolds, A. J. (2013). Adult resilience among maltreated children: A prospective investigation of main effect and mediating models. *Children and Youth Services Review*, *35*(6), 937–949. https://doi.org/10.1016/j.childyouth.2013.03.004

Tucker, C. J., Finkelhor, D., & Turner, H. (2020). Family and friend social support as mediators of adolescent sibling victimization and mental health, self-esteem, and delinquency. *American Journal of Orthopsychiatry, 90*(6), 703 –711. https://dx.doi.org/10.1037/ort0000502

Turanovic, J. J., & Pratt, T. C. (2017). Consequences of Violent Victimization for Native American Youth in Early Adulthood. *Journal of Youth and Adolescence*, *46*(6), 1333–1350. https://doi.org/10.1007/s10964-016-0587-y

van der Put, C. E. (2015). Female adolescent sexual and nonsexual violent offenders: A comparison of the prevalence and impact of risk and protective factors for general recidivism. *BMC Psychiatry*, *15*(1), 1–10.
<https://doi.org/10.1186/s12888-015-0615-6>

van der Put, C. E., & Asscher, J. J. (2015). Protective Factors in Male Adolescents With a History of Sexual and/or Violent Offending: A Comparison Between Three Subgroups. *Sexual Abuse: Journal of Research and Treatment*, *27*(1), 109–126. https://doi.org/10.1177/1079063214549259

van der Put, C. E., Asscher, J. J., & Stams, G. J. J. M. (2012). Differences Between Juvenile Offenders With and Without AD(H)D in Recidivism Rates and Risk and Protective Factors for Recidivism. *Journal of Attention Disorders*, *20*(5), 445–457. https://doi.org/10.1177/1087054712466140

van Loon, L. M. A., van de Ven, M. O. M., van Doesum, K. T. M., Hosman, C. M. H., & Witteman, C. L. M. (2015). Factors Promoting Mental Health of Adolescents Who Have a Parent with Mental Illness: A Longitudinal Study. *Child and Youth Care Forum*, *44*(6), 777–799. https://doi.org/10.1007/s10566-015-9304-3

Vanderbilt-Adriance, E. (2011). *Psychosocial, cognitive, and physiological protective factors and the absence of antisocial behavior in a longitudinal study of low income boys* [Doctoral dissertation, University of Pittsburgh*.* <http://d-scholarship-dev.library.pitt.edu/7974/>

Vargas, D. A., Roosa, M. W., Knight, G. P., & O’Donnell, M. (2013). Family and cultural processes linking family instability to Mexican American adolescent adjustment. *Journal of Family Psychology*, *27*(3), 387–397. https://doi.org/10.1037/a0032863

Vassallo, S., Edwards, B., Renda, J., & Olsson, C. A. (2014). Bullying in Early Adolescence and Antisocial Behavior and Depression Six Years Later: What Are the Protective Factors? *Journal of School Violence*, *13*(1), 100–124. https://doi.org/10.1080/15388220.2013.840643

Vazsonyi, A. T., Chen, P., Jenkins, D. D., Burcu, E., Torrente, G., & Sheu, C. J. (2010). Jessor’s problem behavior theory: Cross-national evidence from Hungary, the Netherlands, Slovenia, Spain, Switzerland, Taiwan, Turkey, and the United States. *Developmental Psychology*, *46*(6), 1779–1791. https://doi.org/10.1037/a0020682

Vazsonyi, A. T., Chen, P., Young, M., Jenkins, D., Browder, S., Kahumoku, E., Pagava, K., Phagava, H., Jeannin, A., & Michaud, P. A. (2008). A Test of Jessor’s Problem Behavior Theory in a Eurasian and a Western European Developmental Context. *Journal of Adolescent Health*, *43*(6), 555–564. https://doi.org/10.1016/j.jadohealth.2008.06.013

Venta, A., Bailey, C., Muñoz, C., Godinez, E., Colin, Y., Arreola, A., Abate, A., Camins, J., Rivas, M., & Lawlace, S. (2018). Contribution of Schools to Mental Health and Resilience in Recently Immigrated Youth. *School Psychology Quarterly*, *34*(2), 138–147. https://doi.org/10.1037/spq0000271

Véronneau, M. H., & Dishion, T. J. (2010). Predicting change in early adolescent problem behavior in the middle school years: a mesosystemic perspective on parenting and peer experiences. Journal of Abnormal Child Psychology, 38(8), 1125–1137. <https://doi.org/10.1007/s10802-010-9431-0>

Villanueva, L., Basto-Pereira, M., & Cuervo, K. (2020). How to Improve the YLS/CMI? Exploring a Particularly Predictive Combination of Items. *International Journal of Offender Therapy and Comparative Criminology*, *64*(9), 922–937. https://doi.org/10.1177/0306624X19881926

Voisin, D. R., Kim, D. H., Bassett, S. M., & Marotta, P. L. (2020). Pathways linking family stress to youth delinquency and substance use: Exploring the mediating roles of self-efficacy and future orientation. *Journal of Health Psychology, 25*(2), 139–151. https://doi.org/10.1177/1359105318763992

Wall, T. D., Sellbom, M., & Goodwin, B. E. (2013). Examination of intelligence as a compensatory factor in non-criminal psychopathy in a non-incarcerated sample. *Journal of Psychopathology and Behavioral Assessment*, *35*(4), 450–459. https://doi.org/10.1007/s10862-013-9358-1

Walters, G. D. (2018). Positive and negative social influences and crime acceleration during the transition from childhood to adolescence: The interplay of risk and protective factors. *Criminal Behaviour and Mental Health*, *28*(5), 414–423. https://doi.org/10.1002/cbm.2088

Walters, G. D. (2020). Prosocial Peers as Risk, Protective, and Promotive Factors for the Prevention of Delinquency and Drug Use. *Journal of Youth and Adolescence*, *49*(3), 618–630. https://doi.org/10.1007/s10964-019-01058-3

Ward, A. (2011). Personality and risk-taking behaviors in emerging adulthood. *Dissertation Abstracts International: Section B: The Sciences and Engineering*, *71*(12-B), 7774. http://search.ebscohost.com/login.aspx?direct=true&db=psyh&AN=2011-99120-048&site=ehost-live

Weeland, J., Laceulle, O. M., Nederhof, E., Overbeek, G., & Reijneveld, S. A. (2019). The greener the better? Does neighborhood greenness buffer the effects of stressful life events on externalizing behavior in late adolescence? *Health and Place*, *58*, 102163. https://doi.org/10.1016/j.healthplace.2019.102163

Weist, M. D., Albus, K. A., Bickham, N., Tashman, N. A., & Perez-Febles, A. (2000). A questionnaire to measure factors that protect youth against stressors of inner-city life. *Psychiatric Services*, *51*(8), 1042–1044. https://doi.org/10.1176/appi.ps.51.8.1042

Whitney, S. D., Renner, L. M., & Herrenkohl, T. I. (2010). Gender differences in risk and promotive classifications associated with adolescent delinquency. *Journal of Genetic Psychology*, *171*(2), 116–138. https://doi.org/10.1080/00221320903548092

Willemen, A. M., Schuengel, C., & Koot, H. M. (2011). Observed interactions indicate protective effects of relationships with parents for referred adolescents. *Journal of Research on Adolescence*, *21*(3), 569–575. https://doi.org/10.1111/j.1532-7795.2010.00703.x

Williams, J. L., Aiyer, S. M., Durkee, M. I., & Tolan, P. H. (2014). The Protective Role of Ethnic Identity for Urban Adolescent Males Facing Multiple Stressors. *Journal of Youth and Adolescence*, *43*(10), 1728–1741. https://doi.org/10.1007/s10964-013-0071-x

Wilson, D., Foster, J., Anderson, S., & Mance, G. (2009). Racial socialization’s moderating effect between poverty stress and psychological symptoms for African American youth. *Journal of Black Psychology*, *35*(1), 102–124. https://doi.org/10.1177/0095798408316368

Wilson, S. R., Chernichky, S. M., Wilkum, K., & Owlett, J. S. (2014). Do Family Communication Patterns Buffer Children from Difficulties Associated with a Parent’s Military Deployment? Examining Deployed and At-home Parents’ Perspectives. *Journal of Family Communication*, *14*(1), 32–52. https://doi.org/10.1080/15267431.2013.857325

Wojciak, A. S., Thompson, H. M., & Cooley, M. E. (2017). The Relationship Between Caregivers and Youth in Foster Care: Examining the Relationship for Mediation and Moderation Effects on Youth Behaviors. *Journal of Emotional and Behavioral Disorders*, *25*(2), 96–106. https://doi.org/10.1177/1063426616628816

Wolff, K. T., Baglivio, M. T., Intravia, J., & Piquero, A. R. (2015). The protective impact of immigrant concentration on juvenile recidivism: A statewide analysis of youth offenders. *Journal of Criminal Justice*, *43*(6), 522–531. https://doi.org/10.1016/j.jcrimjus.2015.05.004

Woodman, A. C., Mailick, M. R., & Greenberg, J. S. (2016). Trajectories of internalizing and externalizing symptoms among adults with autism spectrum disorders. *Development and psychopathology, 28*(2), 565–581. https://doi.org/10.1017/S095457941500108X.

Wright, K. A., Kim, B., Chassin, L., Losoya, S. H., & Piquero, A. R. (2014). Ecological Context, Concentrated Disadvantage, and Youth Reoffending: Identifying the Social Mechanisms in a Sample of Serious Adolescent Offenders. *Journal of Youth and Adolescence*, *43*(10), 1781–1799. https://doi.org/10.1007/s10964-014-0173-0

Xu, Y., Huang, H., & Cao, Y. (2020). Associations among Early Exposure to Neighborhood Disorder, Fathers’ Early Involvement, and Children’s Internalizing and Externalizing Problems. *Journal of Evidence-Based Social Work (United States)*, *17*(5), 558–575. https://doi.org/10.1080/26408066.2020.1782302

Yasui, M., Dorham, C. L. R., & Dishion, T. J. (2004). Ethnic identity and psychological adjustment: A validity analysis for European American and African American adolescents. *Journal of Adolescent Research*, *19*(6), 807–825. https://doi.org/10.1177/0743558403260098

Yeung, R., & Leadbeater, B. (2010). Adults make a difference: the protective effects of parent and teacher emotional support on emotional and behavioral problems of peer-victimized adolescents. *Journal of Community Psychology*, *38*(1), 80–98. https://doi.org/10.1002/JCOP.20353

Youngstrom, E., Weist, M. D., & Albus, K. E. (2003). Exploring violence exposure, stress, protective factors and behavioral problems among inner-city youth. *American Journal of Community Psychology*, *32*(1–2), 115–129. https://doi.org/10.1023/A:1025607226122

Yun, H. J., & Cui, M. (2020). The Effects of Parental Warmth on Adolescent Delinquency in the United States and South Korea: a Cross-Cultural Perspective. *Journal of Youth and Adolescence, 49*(1), 228–237. <https://doi.org/10.1007/s10964-019-01078-z>

Zaharakis, N. M., Mason, M. J., Brown, A., Moore, M., Garcia, C., Foster, R., & Richards, S. (2018). Resiliency Moderates the Influence of Somatization on Externalizing Problems. *Journal of Child and Family Studies*, *27*(9), 2978–2989. https://doi.org/10.1007/s10826-018-1141-1

Zapolski, T. C. B., Garcia, C. A., Jarjoura, G. R., Lau, K. S. L., & Aalsma, M. C. (2016). Examining the Influence of Ethnic/Racial Socialization on Aggressive Behaviors Among Juvenile Offenders. *Journal of Juvenile Justice*, *5*(1), 65–79. http://www.ncbi.nlm.nih.gov/pubmed/27453798%0Ahttp://www.pubmedcentral.nih.gov/articlerender.fcgi?artid=PMC4946249

Zimmerman, M. A., Ramirez-Valles, J., Zapert, K. M., & Maton, K. I. (2000). A longitudinal study of stress-buffering effects for urban African-American male adolescent problem behaviors and mental health. *Journal of Community Psychology*, *28*(1), 17–33. https://doi.org/10.1002/(SICI)1520-6629(200001)28:1<17::AID-JCOP4>3.0.CO;2-I

Zinn, M. E., Huntley, E. D., & Keating, D. P. (2020). Resilience in adolescence: Prospective Self moderates the association of early life adversity with externalizing problems. *Journal of Adolescence*, *81*(July 2019), 61–72. https://doi.org/10.1016/j.adolescence.2020.04.004

Appendix S3

Characteristics of Included Studies

| Authors, publication year | *N* | Mean age of sample | Mean proportion males in sample | #Protective factors | #Individual factors | #Family factors | #Non-family factors | #Community factors | #School factors |
| --- | --- | --- | --- | --- | --- | --- | --- | --- | --- |
| Aceves & Cookston, 2007 | 1848 | 15.50 | 0.50 | 4 | 0 | 4 | 0 | 0 | 0 |
| Al-Yagon, 2015 | 185 | 16.00 | 0.49 | 2 | 0 | 2 | 0 | 0 | 0 |
| Allbaugh, 2018 | 514 | 17.63 | 0.00 | 8 | 0 | 8 | 0 | 0 | 0 |
| Álvarez-García et al., 2019 | 3199 | 14.03 | 0.51 | 3 | 1 | 2 | 0 | 0 | 0 |
| Anderson et al., 2015 | 1072 | 16.03 | 0.49 | 9 | 3 | 3 | 3 | 0 | 0 |
| Anderson et al., 2017 | 2154 | 21.00 | 0.38 | 1 | 1 | 0 | 0 | 0 | 0 |
| Appleyard et al., 2007 | 184 | 15.50 | 0.55 | 3 | 0 | 1 | 2 | 0 | 0 |
| Apter et al., 1997 | 223 | 16.00 | 0.50 | 1 | 1 | 0 | 0 | 0 | 0 |
| Aquilar et al., 2018 | 314 | 15.33 | 0.56 | 12 | 12 | 0 | 0 | 0 | 0 |
| Aronowitz & Morrison-Beedy, 2004 | 443 | 14.00 | 0.00 | 6 | 0 | 6 | 0 | 0 | 0 |
| Aspy et al., 2004 | 1090 | 14.50 | 0.49 | 18 | 10 | 2 | 4 | 2 | 0 |
| Bacchini et al., 2011 | 489 | 18.00 | 0.59 | 1 | 0 | 1 | 0 | 0 | 0 |
| Bacon et al., 2014 | 48 | 20.00 | 1.00 | 1 | 1 | 0 | 0 | 0 | 0 |
| Baier | 3800 | 15.11 | 0.44 | 1 | 1 | 0 | 0 | 0 | 0 |
| Bakker et al., 2011 | 1816 | 16.27 | 0.48 | 2 | 2 | 0 | 0 | 0 | 0 |
| Banerjee, 2012 | 281 | 20.00 | 0.24 | 4 | 0 | 4 | 0 | 0 | 0 |
| Barger et al., 2017 | 100 | 17.10 | 0.35 | 7 | 6 | 0 | 1 | 0 | 0 |
| Barnes et al., 2007 | 606 | 17.00 | 0.45 | 4 | 1 | 2 | 0 | 0 | 1 |
| Barnes-Lee & Campbell, 2020 | 278 | 15,37 | 0.67 | 15 | 7 | 2 | 3 | 2 | 1 |
| Barry et al., 2015 | 251 | 17.00 | 1.00 | 2 | 2 | 0 | 0 | 0 | 0 |
| Barry et al., 2017 | 161 | 17.00 | 0.80 | 1 | 1 | 0 | 0 | 0 | 0 |
| Bates et al., 2017 | 198 | 24.00 | 0.50 | 12 | 12 | 0 | 0 | 0 | 0 |
| Beam et al., 2002 | 243 | 17.00 | 0.43 | 5 | 0 | 3 | 2 | 0 | 0 |
| Beaver et al., 2011 | 457 | 28.00 | 0.50 | 20 | 8 | 0 | 0 | 4 | 8 |
| Beckmann, 2021 | 7496 | 14.86 | 0.48 | 2 | 0 | 2 | 0 | 0 | 0 |
| Beckmann et al., 2017 | 2878 | 15.00 | 0.50 | 12 | 4 | 8 | 0 | 0 | 0 |
| Bernat et al., 2012 | 1132 | 16.50 | 0.43 | 24 | 6 | 4 | 6 | 2 | 6 |
| Blazei et al., 2008 | 606 | 17.00 | 0.46 | 5 | 0 | 5 | 0 | 0 | 0 |
| Blum et al., 2003 | 8612 | 14.50 | 0.40 | 5 | 0 | 2 | 0 | 0 | 3 |
| Boccio, 2018 | 6872 | 23.00 | 0.49 | 13 | 4 | 6 | 1 | 2 | 0 |
| Bondü & Elsner, 2015 | 1222 | 13.34 | 0.50 | 2 | 2 | 0 | 0 | 0 | 0 |
| Botcheva et al., 2002 | 104 | 14.00 | 0.73 | 4 | 0 | 2 | 1 | 0 | 1 |
| Boyas et al., 2017 | 2328 | 14.50 | 0.49 | 7 | 2 | 4 | 0 | 0 | 1 |
| Brieant et al., 2018 | 132 | 14.50 | 0.52 | 2 | 2 | 0 | 0 | 0 | 0 |
| Brody et al., 2013 | 489 | 19.00 | 0.47 | 1 | 1 | 0 | 0 | 0 | 0 |
| Brookmeyer et al., 2006 | 6397 | 16.04 | 0.46 | 4 | 0 | 2 | 0 | 0 | 2 |
| Browning & Erickson, 2012 | 983 | 15.00 | - | 2 | 0 | 1 | 0 | 0 | 1 |
| Bruett, 2015 | 627 | 16.00 | 0.73 | 12 | 4 | 8 | 0 | 0 | 0 |
| Buote, 2006 | 94 | 16.00 | 0.46 | 6 | 2 | 0 | 3 | 0 | 1 |
| Burnside, 2015 | 65 | 16.00 | 0.40 | 14 | 6 | 0 | 6 | 0 | 2 |
| Burt et al., 2012 | 306 | 18.50 | 1.00 | 1 | 0 | 1 | 0 | 0 | 0 |
| Caiozzo et al., 2016 | 591 | 19.00 | 0.50 | 2 | 2 | 0 | 0 | 0 | 0 |
| Caldwell & Smith, 2013 | 475 | 16.00 | 0.49 | 7 | 5 | 1 | 0 | 0 | 1 |
| Caldwell et al., 2004 | 325 | 20.00 | 0.45 | 3 | 3 | 0 | 0 | 0 | 0 |
| Calvete et al., 2017 | 818 | 16.50 | 0.47 | 2 | 2 | 0 | 0 | 0 | 0 |
| Calzada et al., 2020 | 547 | 13.00 | 0.45 | 3 | 0 | 3 | 0 | 0 | 0 |
| Campos et al., 2019 | 443 | 15.00 | 0.50 | 9 | 0 | 6 | 3 | 0 | 0 |
| Carlson et al., 2015 | 361 | 19.00 | 0.38 | 4 | 4 | 0 | 0 | 0 | 0 |
| Cavanaugh et al., 2018 | 174 | 12.50 | 0.49 | 8 | 6 | 2 | 0 | 0 | 0 |
| Champion et al., 2008 | 2090 | 16.00 | 0.51 | 8 | 0 | 1 | 0 | 5 | 2 |
| Chapman, 2018 | 697 | 15.00 | 0.38 | 2 | 0 | 1 | 0 | 0 | 1 |
| Chappel, 2013 | 425 | 16.00 | 0.40 | 6 | 0 | 2 | 0 | 0 | 4 |
| Chen et al., 2016 | 2980 | 12.48 | 0.41 | 4 | 1 | 1 | 0 | 1 | 1 |
| Chinchilla & Kosson, 2016 | 214 | 15.37 | 0.86 | 1 | 0 | 1 | 0 | 0 | 0 |
| Choi et al., 2005 | 2055 | 13.00 | 0.50 | 8 | 0 | 8 | 0 | 0 | 0 |
| Chuang et al., 2006 | 59 | 15.00 | 0.50 | 4 | 4 | 0 | 0 | 0 | 0 |
| Chung & Docherty, 2011 | 127 | 21.05 | 0.49 | 1 | 0 | 0 | 0 | 1 | 0 |
| Copeland-Linder et al., 2011 | 250 | 16.00 | 0.50 | 6 | 2 | 2 | 0 | 0 | 2 |
| Copeland-Linder et al., 2010 | 504 | 12.23 | 0.54 | 3 | 1 | 2 | 0 | 0 | 0 |
| Coyne et al., 2014 | 491 | 14.00 | 0.47 | 6 | 0 | 6 | 0 | 0 | 0 |
| Craig et al., 2017 | 28169 | 18.00 | 0.77 | 1 | 0 | 0 | 0 | 1 | 0 |
| Criss et al., 2017; Cropp et al., 2019 | 206 | 13.00 | 0.49 | 6 | 0 | 3 | 3 | 0 | 0 |
| Cropp et al., 2019 | 96 | 16.00 | 0.52 | 1 | 1 | 0 | 0 | 0 | 0 |
| Culyba et al., 2016 | 283 | 17.80 | 1.00 | 4 | 0 | 4 | 0 | 0 | 0 |
| Cunningham et al., 2018 | 141 | 15.00 | 0.50 | 6 | 6 | 0 | 0 | 0 | 0 |
| Curran & Chassin, 1996 | 278 | 13.50 | 0.55 | 6 | 0 | 6 | 0 | 0 | 0 |
| Cutrín et al., 2017 | 438 | 16.50 | 0.69 | 8 | 0 | 8 | 0 | 0 | 0 |
| Davis, 2017 | 549 | 20.30 | 0.30 | 3 | 2 | 0 | 0 | 1 | 0 |
| de la Barrera et al., 2019 | 840 | 13.00 | 0.49 | 4 | 4 | 0 | 0 | 0 | 0 |
| de la Rubia & Morales, 2012 | 193 | 15.70 | 0.57 | 2 | 1 | 1 | 0 | 0 | 0 |
| de Vries et al., 2016 | 102 | 15.52 | 0.71 | 15 | 3 | 12 | 0 | 0 | 0 |
| Deane et al., 2018 | 119 | 13.50 | 0.50 | 16 | 0 | 16 | 0 | 0 | 0 |
| DeGruy et al., 2012 | 200 | 16.10 | 1.00 | 2 | 2 | 0 | 0 | 0 | 0 |
| DeSantis King et al., 2006 | 974 | 15.00 | 0.35 | 6 | 0 | 1 | 1 | 1 | 3 |
| DuPont-Reyes et al., 2015 | 339 | 16.00 | 0.50 | 2 | 2 | 0 | 0 | 0 | 0 |
| El-Sheikh et al., 2020 | 272 | 17.27 | 0.51 | 4 | 3 | 1 | 0 | 0 | 0 |
| Ernst, 2000 | 167 | 29.00 | - | 18 | 0 | 18 | 0 | 0 | 0 |
| Estévez López et al., 2008 | 660 | 13.70 | 0.50 | 36 | 6 | 12 | 0 | 0 | 18 |
| Farruggia & Germo, 2015 | 188 | 18.00 | 0.50 | 12 | 0 | 8 | 4 | 0 | 0 |
| Fernández-González et al., 2018 | 543 | 16.50 | 0.48 | 12 | 12 | 0 | 0 | 0 | 0 |
| Fitzpatrick & Bussey, 2014 | 1218 | 14.00 | 0.50 | 2 | 0 | 0 | 2 | 0 | 0 |
| Flouri & Mavroveli, 2013 | 159 | 14.33 | 0.42 | 6 | 6 | 0 | 0 | 0 | 0 |
| Flouri et al., 2013 | 1599 | 15.50 | - | 2 | 2 | 0 | 0 | 0 | 0 |
| Folger & Wright, 2013 | 344 | 20.00 | 0.47 | 2 | 0 | 1 | 1 | 0 | 0 |
| Forster et al., 2017 | 94 | 22.20 | 0.39 | 2 | 2 | 0 | 0 | 0 | 0 |
| Froidevaux et al., 2020 | 137 | 15.20 | 0.34 | 2 | 0 | 2 | 0 | 0 | 0 |
| Fung & Lau, 2009 | 107 | 11.90 | 0.58 | 2 | 0 | 0 | 2 | 0 | 0 |
| Garaigordobil, 2015 | 286 | 14.72 | 0.52 | 1 | 1 | 0 | 0 | 0 | 0 |
| Gardner et al., 2008 | 803 | 18.00 | 0.51 | 2 | 2 | 0 | 0 | 0 | 0 |
| Garrido & Taussig, 2013 | 41 | 13.59 | 0.34 | 2 | 0 | 1 | 1 | 0 | 0 |
| Gartstein et al., 2014 | 714 | 17.00 | 0.54 | 1 | 0 | 1 | 0 | 0 | 0 |
| Gerard & Buehler, 2004 | 5070 | 14.50 | 0.51 | 14 | 4 | 2 | 2 | 4 | 2 |
| Germãn et al., 2009 | 598 | 12.30 | 0.49 | 12 | 0 | 12 | 0 | 0 | 0 |
| Gillis & El-Sheikh, 2019 | 235 | 15.78 | 0.47 | 2 | 2 | 0 | 0 | 0 | 0 |
| Gómez-Leal et al., 2020 | 282 | 22.02 | - | 2 | 2 | 0 | 0 | 0 | 0 |
| Goodrum et al., 2015 | 81 | 15.68 | - | 4 | 0 | 2 | 1 | 0 | 1 |
| Governale, 2019 | 278 | 18.13 | 0.46 | 20 | 20 | 0 | 0 | 0 | 0 |
| Gresham et al., 2021 | 84 | 13.36 | 0.50 | 30 | 30 | 0 | 0 | 0 | 0 |
| Grych et al., 2004 | 338 | 16.29 | 0.40 | 4 | 0 | 4 | 0 | 0 | 0 |
| Guerrero et al., 2006 | 216 | 15.86 | 0.52 | 2 | 0 | 1 | 0 | 0 | 1 |
| Gutiérrez-Cobo et al., 2018 | 406 | 21.60 | 0.25 | 6 | 6 | 0 | 0 | 0 | 0 |
| Hall, 2006 | 84 | 21.00 | 0.19 | 6 | 6 | 0 | 0 | 0 | 0 |
| Hallfors et al., 2006 | 465 | 15.50 | 0.49 | 2 | 0 | 0 | 0 | 0 | 2 |
| Hanniball et al., 2021 | 334 | 13.10 | 0.44 | 4 | 4 | 0 | 0 | 0 | 0 |
| Hardaway et al., 2012 | 391 | 14.92 | 0.50 | 9 | 3 | 3 | 0 | 0 | 3 |
| Hardaway et al., 2016 | 312 | 16.49 | 0.50 | 2 | 0 | 2 | 0 | 0 | 0 |
| Hart et al., 2005 | 63 | 15.80 | 0.46 | 1 | 1 | 0 | 0 | 0 | 0 |
| Hemphill et al., 2009 | 3818 | 14.00 | 0.50 | 6 | 1 | 1 | 0 | 2 | 2 |
| Henrich et al., 2005 | 7033 | 16.50 | 0.48 | 8 | 0 | 4 | 0 | 0 | 4 |
| Henry et al., 1999 | 1037 | 21.00 | 0.52 | 2 | 0 | 0 | 0 | 0 | 2 |
| Henry et al., 2012 | 4432 | 13.50 | 0.49 | 3 | 0 | 1 | 0 | 0 | 2 |
| Henry et al., 2015 | 106 | 15.41 | 0.43 | 2 | 0 | 2 | 0 | 0 | 0 |
| Herrenkohl et al., 2003 | 154 | 18.00 | 0.51 | 11 | 2 | 3 | 1 | 2 | 3 |
| Herrenkohl et al., 2005 | 416 | 18.24 | 0.55 | 12 | 6 | 0 | 3 | 0 | 3 |
| Herrenkohl et al., 2009 | 1942 | 15.10 | 0.50 | 6 | 0 | 2 | 0 | 0 | 4 |
| Hesse et al., 2019 | 381 | 18.90 | - | 30 | 30 | 0 | 0 | 0 | 0 |
| Hill et al., 2018 | 970 | 20.00 | - | 2 | 1 | 1 | 0 | 0 | 0 |
| Hinnant & Forman-Alberti, 2019 | 991 | 15.00 | 0.47 | 15 | 15 | 0 | 0 | 0 | 0 |
| Hishinuma et al., 2005 | 2732 | 15.59 | 0.48 | 5 | 1 | 1 | 1 | 0 | 2 |
| Hishinuma et al., 2012 | 881 | 15.80 | 0.40 | 5 | 1 | 2 | 2 | 0 | 0 |
| Hofer et al., 2010 | 182 | 16.69 | 0.50 | 15 | 15 | 0 | 0 | 0 | 0 |
| Hope & Buhs, 2020 | 975 | 14.44 | 0.50 | 2 | 1 | 0 | 0 | 0 | 1 |
| Houtepen et al., 2019 | 866 | 13.84 | 0.51 | 4 | 2 | 2 | 0 | 0 | 0 |
| Huntington, 2014 | 27 | 13.10 | 1.00 | 6 | 6 | 0 | 0 | 0 | 0 |
| Ibabe et al., 2013 | 687 | 14.70 | 0.50 | 22 | 0 | 10 | 0 | 0 | 12 |
| Ibabe, 2016 | 584 | 14.55 | 0.48 | 4 | 0 | 4 | 0 | 0 | 0 |
| Ivanova & Israel, 2006 | 70 | 12.23 | 0.49 | 2 | 0 | 2 | 0 | 0 | 0 |
| Jäggi et al., 2020 | 310 | 16.98 | 0.91 | 20 | 0 | 0 | 0 | 0 | 20 |
| Janssen et al., 2017 | 603 | 15.30 | 0.52 | 4 | 0 | 4 | 0 | 0 | 0 |
| Jennings et al., 2010 | 383 | 14.00 | 0.42 | 10 | 0 | 5 | 0 | 0 | 5 |
| Jessor et al., 1995 | 1591 | 13.50 | 0.43 | 7 | 3 | 2 | 1 | 0 | 1 |
| Jiménez et al., 2021 | 2399 | 14.65 | 0.50 | 30 | 0 | 0 | 0 | 0 | 30 |
| Johns et al., 2021 | 509 | 15.45 | 0.44 | 2 | 1 | 0 | 0 | 0 | 1 |
| Johnson, 2005 | 4090 | 15.20 | 0.48 | 4 | 0 | 4 | 0 | 0 | 0 |
| Jolliffe et al., 2016 | 503 | 16.00 | 1.00 | 8 | 2 | 5 | 0 | 0 | 1 |
| Jones, 2011 | 1018 | 14.61 | 0.71 | 5 | 5 | 0 | 0 | 0 | 0 |
| Jung & Schröder-Abé, 2019 | 1663 | 14.15 | 0.50 | 2 | 2 | 0 | 0 | 0 | 0 |
| Kalaitzaki, 2019 | 807 | 20.89 | 0.28 | 4 | 0 | 4 | 0 | 0 | 0 |
| Kapetanovic & Skoog, 2021 | 1780 | 13.01 | 0.49 | 10 | 0 | 10 | 0 | 0 | 0 |
| Kaufmann et al., 2007 | 167 | 14.00 | 0.51 | 2 | 2 | 0 | 0 | 0 | 0 |
| Kaufmann, 2008 | 169 | 14.00 | 0.51 | 3 | 1 | 1 | 0 | 0 | 1 |
| Kauten et al., 2013 | 154 | 17.05 | 0.84 | 1 | 1 | 0 | 0 | 0 | 0 |
| Kendler et al., 2016 | 4751 | 21.00 | - | 15 | 15 | 0 | 0 | 0 | 0 |
| Khafi et al., 2014 | 143 | 14.89 | 0.48 | 4 | 0 | 4 | 0 | 0 | 0 |
| Khurana et al., 2019 | 1990 | 15.60 | 0.52 | 3 | 0 | 3 | 0 | 0 | 0 |
| Kim-Ju et al., 2017 | 184 | 21.14 | 0.59 | 2 | 1 | 1 | 0 | 0 | 0 |
| Kincaid, 2012 | 193 | 14.55 | 0.44 | 48 | 18 | 30 | 0 | 0 | 0 |
| Kingsbury et al., 2020 | 5913 | 12.50 | - | 3 | 0 | 0 | 0 | 3 | 0 |
| Kokko et al., 2006 | 1025 | 12.00 | 1.00 | 4 | 4 | 0 | 0 | 0 | 0 |
| Korol & Bevelander, 2021 | 365 | 13.93 | 0.54 | 24 | 4 | 20 | 0 | 0 | 0 |
| Kosterman et al., 2005 | 808 | 21.30 | 0.47 | 7 | 5 | 0 | 0 | 2 | 0 |
| Kramer-Kuhn & Farrell, 2016 | 537 | 15.00 | 0.65 | 7 | 0 | 7 | 0 | 0 | 0 |
| Kuhn, 2019 | 150 | 13.00 | 0.49 | 6 | 2 | 4 | 0 | 0 | 0 |
| Kuruczova et al., 2020 | 3261 | 16.50 | - | 2 | 2 | 0 | 0 | 0 | 0 |
| Lagasse et al., 2016 | 1388 | 13.00 | 0.52 | 2 | 2 | 0 | 0 | 0 | 0 |
| Laird et al., 2011 | 166 | 13.00 | 0.51 | 8 | 4 | 4 | 0 | 0 | 0 |
| Lansford et al., 2006 | 585 | 13.00 | 0.52 | 5 | 1 | 4 | 0 | 0 | 0 |
| Lantagne & Furman, 2019 | 137 | 22.07 | - | 6 | 0 | 0 | 6 | 0 | 0 |
| Le & Johansen, 2011 | 324 | 12.50 | - | 5 | 2 | 0 | 1 | 1 | 1 |
| Leadbeater et al., 2008 | 149 | 16.50 | 0.34 | 5 | 0 | 5 | 0 | 0 | 0 |
| Legault et al., 2006 | 220 | 15.30 | 0.50 | 5 | 2 | 2 | 1 | 0 | 0 |
| Linver et al., 2009 | 1711 | 13.80 | 0.50 | 4 | 3 | 0 | 0 | 0 | 1 |
| Liu & Miller, 2020 | 2087 | 15.29 | 0.53 | 6 | 0 | 4 | 0 | 0 | 2 |
| Liu et al., 2017 | 592 | 15.90 | 0.49 | 1 | 0 | 0 | 0 | 1 | 0 |
| Livingston et al., 2018 | 227 | 13.00 | - | 10 | 0 | 10 | 0 | 0 | 0 |
| Lloyd, 2021 | 1036 | 12.51 | 0.52 | 10 | 0 | 10 | 0 | 0 | 0 |
| Longobardi et al., 2016 | 122 | 13.50 | 0.45 | 3 | 1 | 0 | 0 | 0 | 2 |
| Loukas et al., 2008 | 449 | 12.63 | 0.48 | 16 | 0 | 16 | 0 | 0 | 0 |
| Low et al., 2013; Lyons et al., 2014 | 346 | 12.61 | 0.49 | 4 | 4 | 0 | 0 | 0 | 0 |
| Lyons et al., 2014 | 470 | 12.94 | 0.54 | 3 | 3 | 0 | 0 | 0 | 0 |
| Machell et al., 2016 | 1256 | 14.49 | 0.53 | 8 | 8 | 0 | 0 | 0 | 0 |
| Mahoney & Vest, 2012 | 1115 | 19.73 | - | 6 | 6 | 0 | 0 | 0 | 0 |
| Manzi et al., 2014 | 170 | 19.01 | 0.52 | 6 | 0 | 6 | 0 | 0 | 0 |
| Marsiglia et al., 2009 | 151 | 15.53 | 0.40 | 6 | 0 | 6 | 0 | 0 | 0 |
| Marston, 2011 | 184 | 18.50 | 0.48 | 18 | 0 | 0 | 18 | 0 | 0 |
| Mattson et al., 2020 | 1100 | 13.30 | 0.47 | 4 | 0 | 2 | 0 | 1 | 1 |
| Mauer et al., 2022 | 2173 | 14.10 | 0.47 | 1 | 0 | 0 | 1 | 0 | 0 |
| McGee, 2003 | 500 | 15.00 | - | 3 | 3 | 0 | 0 | 0 | 0 |
| McGee, 2015 | 453 | 15.00 | - | 3 | 3 | 0 | 0 | 0 | 0 |
| McKelvey et al., 2015 | 985 | 18.00 | 0.50 | 1 | 0 | 1 | 0 | 0 | 0 |
| McMahon et al., 2013 | 266 | 13.00 | - | 12 | 12 | 0 | 0 | 0 | 0 |
| Meltzer et al., 2011 | 2992 | 15.00 | 0.52 | 6 | 6 | 0 | 0 | 0 | 0 |
| Mihalec-Adkins & Cooley, 2020 | 235 | 14.24 | 0.46 | 6 | 4 | 0 | 0 | 0 | 2 |
| Mikami & Hinshaw, 2006 | 209 | 14.50 | 0.00 | 5 | 1 | 0 | 1 | 0 | 3 |
| Mirza, 2015 | 57 | 14.86 | 0.40 | 5 | 4 | 0 | 0 | 1 | 0 |
| Mitchell et al., 2016 | 51 | 15.00 | 0.49 | 4 | 0 | 4 | 0 | 0 | 0 |
| Mohammad et al., 2014 | 91 | 12.32 | 0.45 | 1 | 1 | 0 | 0 | 0 | 0 |
| Monahan et al., 2000 | 939 | 29.90 | 0.57 | 2 | 1 | 0 | 0 | 1 | 0 |
| Morimoto & Sharma, 2004 | 197 | 18.90 | 0.26 | 4 | 1 | 3 | 0 | 0 | 0 |
| Moyer, 2008 | 195 | 12.00 | 0.53 | 5 | 5 | 0 | 0 | 0 | 0 |
| Munir & Malik, 2020 | 706 | 17.01 | 0.45 | 5 | 3 | 1 | 1 | 0 | 0 |
| Neaverson et al., 2020 | 1144 | 14.33 | 0.51 | 6 | 6 | 0 | 0 | 0 | 0 |
| Okeem, 2018 | 201 | 22.90 | 0.42 | 3 | 3 | 0 | 0 | 0 | 0 |
| Oliva et al., 2009 | 112 | 14.62 | 0.43 | 3 | 0 | 3 | 0 | 0 | 0 |
| Oliver & Hodgins, 2013 | 742 | 16.00 | 0.00 | 8 | 2 | 6 | 0 | 0 | 0 |
| Oshri et al., 2013 | 400 | 17.50 | - | 2 | 2 | 0 | 0 | 0 | 0 |
| Oudekerk et al., 2012 | 141 | 16.73 | 0.00 | 2 | 0 | 0 | 0 | 0 | 2 |
| Pardini et al., 2012 | 503 | 16.00 | 1.00 | 8 | 2 | 2 | 0 | 0 | 4 |
| Pastor, 2020 | 610 | 13.38 | 0.52 | 10 | 4 | 1 | 3 | 0 | 2 |
| Paterson et al., 2022 | 806 | 28.20 | 0.00 | 6 | 6 | 0 | 0 | 0 | 0 |
| Paysnick & Burt, 2015 | 66 | 16.60 | 0.40 | 2 | 2 | 0 | 0 | 0 | 0 |
| Pearce et al., 2003 | 1703 | 12.50 | 0.47 | 10 | 8 | 2 | 0 | 0 | 0 |
| Perryman, 2016 | 161 | 20.50 | 0.35 | 2 | 1 | 1 | 0 | 0 | 0 |
| Petit et al., 2017 | 156 | 25.02 | - | 2 | 0 | 0 | 2 | 0 | 0 |
| Pierre et al., 2020 | 119 | 15.95 | 1.00 | 2 | 0 | 0 | 0 | 0 | 2 |
| Pierre, 2016 | 187 | 15.98 | 1.00 | 11 | 5 | 2 | 2 | 2 | 0 |
| Powell et al., 2021 | 374 | 20.00 | 0.00 | 2 | 0 | 2 | 0 | 0 | 0 |
| Preece, 2015 | 9625 | 13.50 | 0.51 | 6 | 0 | 0 | 0 | 0 | 6 |
| Prelow et al., 2007 | 316 | 16.36 | - | 5 | 2 | 1 | 0 | 0 | 2 |
| Prince et al., 2019 | 4991 | 19.00 | - | 1 | 0 | 0 | 1 | 0 | 0 |
| Puckett, 2011 | 256 | 15.00 | 0.83 | 4 | 0 | 2 | 2 | 0 | 0 |
| Rapp-Paglicci et al., 2011 | 108 | 16.80 | 0.51 | 4 | 0 | 0 | 0 | 0 | 4 |
| Reardon et al., 2016 | 106 | 16.01 | 0.44 | 6 | 6 | 0 | 0 | 0 | 0 |
| Reingle et al., 2013 | 4322 | 15.30 | 1.00 | 2 | 0 | 0 | 0 | 1 | 1 |
| Resnick et al., 1997 | 12118 | 15.00 | - | 4 | 0 | 2 | 0 | 0 | 2 |
| Richardson et al., 2019 | 83 | 14.96 | 0.28 | 1 | 0 | 0 | 1 | 0 | 0 |
| Rious & Cunningham, 2018 | 207 | 15.78 | 0.34 | 3 | 3 | 0 | 0 | 0 | 0 |
| Roberts et al., 2009 | 3134 | 15.00 | 0.51 | 6 | 4 | 2 | 0 | 0 | 0 |
| Roberts, 2017 | 374 | 14.00 | 0.39 | 10 | 0 | 6 | 4 | 0 | 0 |
| Romont, 2012 | 116 | 15.95 | 0.00 | 5 | 2 | 1 | 0 | 0 | 2 |
| Rosky, 2010 | 12616 | 22.40 | 0.48 | 8 | 5 | 3 | 0 | 0 | 0 |
| Ross et al., 2023 | 141 | 19.90 | 0.39 | 9 | 3 | 2 | 0 | 2 | 2 |
| Salas-Wright, et al., 2015a | 138 | 18.88 | 0.00 | 9 | 6 | 3 | 0 | 0 | 0 |
| Salas-Wright, et al., 2015b | 22033 | 21.91 | 0.51 | 4 | 3 | 0 | 1 | 0 | 0 |
| Salzinger et al., 2011 | 667 | 13.00 | 0.50 | 3 | 1 | 1 | 1 | 0 | 0 |
| Sasser et al., 2019 | 1354 | 16.32 | 0.48 | 5 | 1 | 2 | 1 | 1 | 0 |
| Scarpa & Haden, 2006 | 515 | 19.50 | 0.31 | 4 | 2 | 1 | 1 | 0 | 0 |
| Schmidt et al., 2012 | 4306 | 15.90 | 0.50 | 2 | 1 | 0 | 0 | 0 | 1 |
| Schnurr, 2009 | 765 | 17.81 | 0.47 | 2 | 0 | 0 | 0 | 2 | 0 |
| Schriber et al., 2018 | 229 | 17.16 | 0.51 | 7 | 0 | 7 | 0 | 0 | 0 |
| Scott et al., 2019 | 254 | 17.60 | 0.58 | 5 | 4 | 0 | 0 | 1 | 0 |
| Segura et al., 2017 | 127 | 14.60 | 0.49 | 5 | 5 | 0 | 0 | 0 | 0 |
| Sharma et al., 2019 | 576 | 16.00 | 0.47 | 3 | 1 | 1 | 0 | 1 | 0 |
| Shorey et al., 2014 | 109 | 18.44 | 1.00 | 1 | 0 | 0 | 1 | 0 | 0 |
| Shorey et al., 2016 | 398 | 41.51 | 1.00 | 4 | 4 | 0 | 0 | 0 | 0 |
| Showalter et al., 2017 | 2345 | 28.37 | - | 2 | 0 | 0 | 0 | 2 | 0 |
| Smokowski et al., 2013 | 4321 | 12.84 | 0.47 | 7 | 2 | 1 | 1 | 1 | 2 |
| So et al., 2018 | 572 | 15.85 | 0.46 | 8 | 8 | 0 | 0 | 0 | 0 |
| Soderstrom et al., 2020 | 460 | 14.79 | 1.00 | 12 | 6 | 0 | 2 | 2 | 2 |
| Spano et al., 2009 | 1544 | 17.00 | 0.51 | 20 | 0 | 20 | 0 | 0 | 0 |
| Sparks et al., 2021 | 1236 | 13.60 | - | 1 | 1 | 0 | 0 | 0 | 0 |
| Spice et al., 2010 | 74 | 16.74 | 0.85 | 8 | 8 | 0 | 0 | 0 | 0 |
| Steiner et al., 2019 | 14800 | 28.30 | 0.51 | 2 | 0 | 1 | 0 | 0 | 1 |
| Stewart & Rapp, 2017 | 138 | 15.20 | 1.00 | 4 | 2 | 2 | 0 | 0 | 0 |
| Stoddard et al., 2015 | 681 | 19.75 | 0.49 | 2 | 0 | 0 | 0 | 0 | 2 |
| Stoddard et al., 2011 | 164 | 12.10 | 0.48 | 3 | 1 | 1 | 0 | 0 | 1 |
| Stoeckel, 2011 | 78 | 12.50 | 0.54 | 2 | 0 | 0 | 0 | 1 | 1 |
| Stouthamer-Loeber et al., 2004 | 506 | 17.69 | 1.00 | 13 | 7 | 1 | 5 | 0 | 0 |
| Stuewig et al., 2010 | 309 | 20.15 | 0.45 | 12 | 12 | 0 | 0 | 0 | 0 |
| Svetaz et al., 2000 | 1603 | 15.50 | 0.67 | 7 | 1 | 4 | 0 | 0 | 2 |
| Szwedo et al., 2017 | 97 | 22.00 | 0.42 | 4 | 1 | 0 | 3 | 0 | 0 |
| Talley, 2019 | 597 | 16.00 | 1.00 | 6 | 6 | 0 | 0 | 0 | 0 |
| Taubner et al., 2013 | 104 | 16.40 | 0.57 | 2 | 2 | 0 | 0 | 0 | 0 |
| Taubner et al., 2016 | 161 | 16.50 | 0.47 | 6 | 4 | 0 | 2 | 0 | 0 |
| Taussig, 2002 | 110 | 15.00 | 0.40 | 10 | 5 | 1 | 1 | 0 | 3 |
| Taylor, 2010 | 204 | 16.00 | - | 1 | 0 | 0 | 0 | 1 | 0 |
| Thomas & Hope, 2016 | 287 | 18.90 | 1.00 | 4 | 0 | 2 | 0 | 2 | 0 |
| Thomas, 2013 | 1153 | 13.50 | 1.00 | 3 | 1 | 1 | 0 | 0 | 1 |
| Tiềt & Huizinga, 2002 | 877 | 13.50 | 0.53 | 2 | 1 | 0 | 0 | 0 | 1 |
| Tomeny et al., 2012 | 168 | 12.81 | 0.61 | 2 | 0 | 2 | 0 | 0 | 0 |
| Topitzes et al., 2013 | 1182 | 14.63 | - | 4 | 2 | 0 | 0 | 0 | 2 |
| Tucker et al., 2021 | 850 | 13.26 | 0.51 | 4 | 0 | 2 | 2 | 0 | 0 |
| Turanovic & Pratt, 2017 | 558 | 22.00 | 0.50 | 4 | 0 | 2 | 0 | 0 | 2 |
| van der Put, 2015 | 219 | 16.50 | 0.00 | 12 | 6 | 2 | 2 | 0 | 2 |
| van der Put & Asscher, 2015 | 635 | 16.50 | 1.00 | 21 | 12 | 3 | 3 | 0 | 3 |
| van der Put et al., 2012 | 1487 | 16.50 | 0.81 | 21 | 12 | 3 | 3 | 0 | 3 |
| van Loon et al., 2015 | 234 | 15.00 | 0.52 | 16 | 4 | 10 | 0 | 2 | 0 |
| Vanderbilt-Adriance, 2011 | 275 | 16.00 | 1.00 | 8 | 6 | 2 | 0 | 0 | 0 |
| Vargas et al., 2013 | 740 | 12.80 | 0.51 | 4 | 0 | 0 | 0 | 0 | 4 |
| Vassallo et al., 2014 | 1016 | 18.00 | 0.50 | 8 | 3 | 3 | 1 | 0 | 1 |
| Vazsonyi et al., 2008 | 8696 | 16.76 | 0.46 | 6 | 0 | 2 | 2 | 0 | 2 |
| Vazsonyi et al., 2010 | 10310 | 16.75 | 0.54 | 2 | 0 | 1 | 0 | 1 | 0 |
| Venta et al., 2018 | 78 | 19.00 | 0.60 | 5 | 0 | 2 | 1 | 0 | 2 |
| Véronneau & Dishion, 2010 | 1278 | 13.92 | 0.45 | 8 | 0 | 2 | 2 | 0 | 4 |
| Villanueva et al., 2020 | 345 | 16.08 | 0.82 | 4 | 3 | 0 | 1 | 0 | 0 |
| Voisin et al., 2020 | 578 | 15.84 | - | 2 | 2 | 0 | 0 | 0 | 0 |
| Wall et al., 2013 | 372 | 18.72 | 0.24 | 3 | 3 | 0 | 0 | 0 | 0 |
| Walters, 2018 | 857 | 16.00 | 0.49 | 2 | 0 | 0 | 0 | 2 | 0 |
| Walters, 2020 | 2905 | 12.57 | 0.48 | 7 | 0 | 4 | 3 | 0 | 0 |
| Ward, 2011 | 255 | 22.00 | 0.20 | 2 | 2 | 0 | 0 | 0 | 0 |
| Weeland et al., 2019 | 715 | 22.30 | 0.49 | 5 | 3 | 0 | 0 | 2 | 0 |
| Weist et al., 2000 | 256 | 15.90 | 0.41 | 2 | 2 | 0 | 0 | 0 | 0 |
| Whitney et al., 2010 | 208 | 18.20 | 0.50 | 10 | 6 | 3 | 1 | 0 | 0 |
| Willemen et al., 2011 | 101 | 13.40 | 0.65 | 1 | 0 | 1 | 0 | 0 | 0 |
| Williams et al., 2014 | 341 | 14.25 | 1.00 | 12 | 12 | 0 | 0 | 0 | 0 |
| Wilson et al., 2009 | 105 | 13.50 | 0.43 | 1 | 1 | 0 | 0 | 0 | 0 |
| Wilson et al., 2014 | 96 | 15.00 | - | 2 | 0 | 2 | 0 | 0 | 0 |
| Wojciak et al., 2017 | 131 | 13.00 | 0.50 | 2 | 0 | 2 | 0 | 0 | 0 |
| Wolff et al., 2015 | 105573 | 17.46 | 0.69 | 2 | 1 | 1 | 0 | 0 | 0 |
| Woodman et al., 2016 | 259 | 21.23 | 0.75 | 22 | 11 | 11 | 0 | 0 | 0 |
| Wright et al., 2014 | 945 | 22.00 | 0.82 | 1 | 0 | 0 | 0 | 1 | 0 |
| Xu et al., 2020 | 3664 | 15.00 | 0.52 | 1 | 0 | 1 | 0 | 0 | 0 |
| Yasui et al., 2004 | 159 | 12.30 | 0.42 | 12 | 12 | 0 | 0 | 0 | 0 |
| Yeung & Leadbeater, 2010 | 622 | 16.55 | 0.48 | 6 | 0 | 4 | 0 | 0 | 2 |
| Youngstrom et al., 2003 | 320 | 14.70 | 0.51 | 2 | 1 | 1 | 0 | 0 | 0 |
| Yun & Cui, 2020 | 5665 | 15.50 | - | 2 | 0 | 2 | 0 | 0 | 0 |
| Zaharakis et al., 2018 | 195 | 15.60 | 0.40 | 3 | 3 | 0 | 0 | 0 | 0 |
| Zapolski et al., 2016 | 95 | 16.30 | 0.72 | 1 | 0 | 0 | 0 | 1 | 0 |
| Zimmerman et al., 2000 | 173 | 17.05 | 1.00 | 4 | 0 | 2 | 2 | 0 | 0 |
| Zinn et al., 2020 | 2017 | 16.80 | 0.45 | 1 | 1 | 0 | 0 | 0 | 0 |

*Note.* *N* = total sample size; #Protective factors = total number of protective factors that were retrieved from the study; #Individual factors = number of individual factors that were retrieved from the study; #Family factors = number of family factors that were retrieved from the study; #Non-family factors = number of non-family factors that were retrieved from the study; #Community factors = number of community factors that were retrieved from the study; #School factors = number of school factors that were retrieved from the study. Dashes indicate that a statistic could not be retrieved from a study.
